# Synthesis and Luminescent Properties of *s*-Tetrazine Derivatives Conjugated with the 4*H*-1,2,4-Triazole Ring

**DOI:** 10.3390/molecules27113642

**Published:** 2022-06-06

**Authors:** Anna Maj, Agnieszka Kudelko, Marcin Świątkowski

**Affiliations:** 1 Department of Chemical Organic Technology and Petrochemistry, The Silesian University of Technology, Krzywoustego 4, PL-44100 Gliwice, Poland; anna.maj@polsl.pl; 2 Institute of General and Ecological Chemistry, Lodz University of Technology, Zeromskiego 116, PL-90924 Lodz, Poland; marcin.swiatkowski@p.lodz.pl

**Keywords:** *s*-tetrazine, 4*H*-1,2,4-triazole, pinner reaction

## Abstract

New derivatives obtained by the combination of unique 1,2,4,5-tetrazine and 4*H*-1,2,4-triazole rings have great application potential in many fields. Therefore, two synthetic few-step methodologies, which make use of commercially available 4-cyanobenzoic acid (method A) and ethyl diazoacetate (method B), were applied to produce two groups of the aforementioned heterocyclic conjugates. In both cases, the target compounds were obtained in various combinations, by introducing electron-donating or electron-withdrawing substituents into the terminal rings, together with aromatic or aliphatic substituents on the triazole nitrogen atom. Synthesis of such designed systems made it possible to analyze the influence of individual elements of the structure on the reaction course, as well as the absorption and emission properties. The structure of all products was confirmed by conventional spectroscopic methods, and their luminescent properties were also determined.

## 1. Introduction

**Scheme 1:**
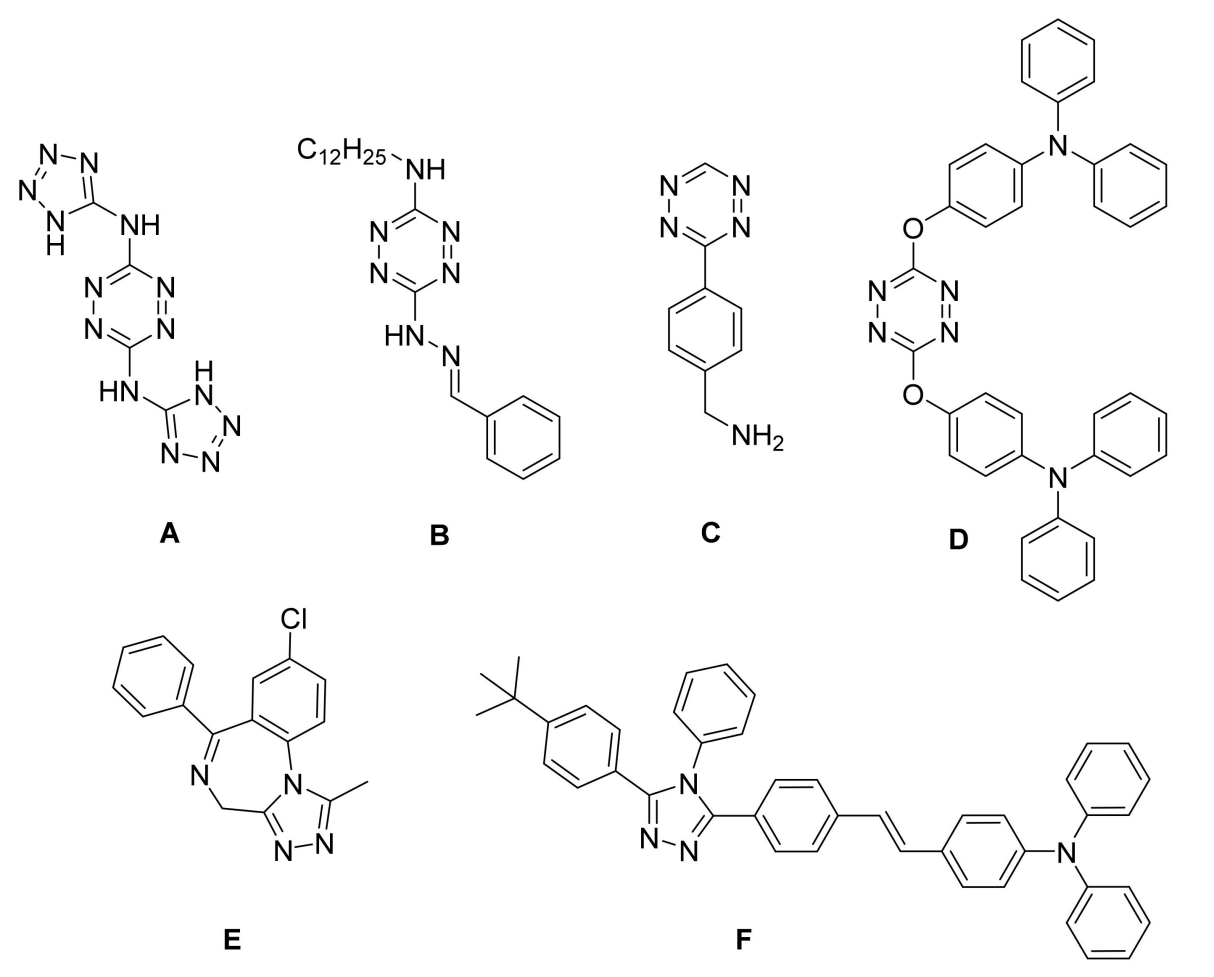
Derivatives of the title heterocycles with great application potential [3,4,7,12,13,17].

Over the years, scientists from around the world have been keen to study heterocyclic organic compounds, and nitrogen-rich systems have proven to be particularly valuable. One of the most interesting areas of this research is the synthesis and properties of 1,2,4,5-tetrazine derivatives (*s*-tetrazine). This unique ring contains four nitrogen atoms, which is the maximum content in a stable six-membered system. This specific structure has attracted scientists’ attention as an important candidate for high energy density materials (HEDMs, A, Scheme 1), as its thermal decomposition leads to ring opening and the release of a nitrogen molecule [1,2,3]. The high nitrogen content has also encouraged research into its biological activity (B, Scheme 1), which has resulted in compounds that have anti-tubercular, anti-cancer, or anti-malarial effects [4,5,6]. Moreover, its high reactivity in Diels–Alder reactions with inverse electron demand determines its application potential in bioorthogonal chemistry (C, Scheme 1) [7,8,9,10]. Important features of the *s*-tetrazine ring are its low-energy n→π electronic transitions, which are especially valuable from the point of view of optoelectronics (Scheme 1). It can be used in the production of organic light-emitting diodes (OLEDs), organic field-effect transistors (OFETs), and solar cells. Due to the high electronegativity of nitrogen, the ring in question is also characterized by a high electron deficit, and thus a high electron affinity. Consequently, it is also a promising building block in ambipolar and n-type materials [11,12].

The five-membered compound, which, like *s*-tetrazine, shows high nitrogen content, is 4*H*-1,2,4-triazole. In this case, too, the presence of nitrogen is associated with a high affinity toward biological macromolecules, which results in biological activity, such as the possession of antiviral, anti-migraine, antifungal, anti-cancer, or psychotropic properties, and various commercially available products incorporate 4*H*-1,2,4-triazole rings (E, Scheme 1) [13,14,15,16]. Another consequence of the nitrogen atoms is the aforementioned change in the electron density distribution, and the associated ability to transport electrons, making it an acceptor unit (F, Scheme 1). Therefore, 4*H*-1,2,4-triazole derivatives are often used in the production of blue OLEDs [17,18,19,20].

Many synthetic methods can be found in the literature for both *s*-tetrazine and 4*H*-1,2,4-triazole derivatives. The five-membered heterocycle is usually obtained from acyclic compounds such as *N*,*N’*-diacylhydrazines, *N*-cyanoguanidine, isothiocyanates, hydrazides, aminoethylidenehydrazones, aldehydes, and semicarbazides [21]. For the six-membered *s*-tetrazine system, the Pinner method is the most popular: cyclization, supported by an activating agent, occurs as a result of the reaction of carbonitriles with hydrazine hydrate. The product of this transformation is the corresponding dihydro derivative that requires oxidation to give the desired ring [22,23]. This approach is distinguished by a wide range of substrates, but also the ability to synthesize both symmetrical and unsymmetrical products. Our research to date proves that, among its other uses, it is perfect for the preparation of complex conjugated systems that contain additional five-membered rings. In recent years, we have successfully synthesized *s*-tetrazine conjugated via a 1,4-phenylene linker with a range of 1,3,4-oxadiazoles, 1,3,4-thiadiazoles and 4*H*-1,2,4-triazoles; however, in the latter case, we have so far only obtained symmetrical systems [24,25,26]. In a continuation of our research, we decided to use the Pinner method to prepare unsymmetrical ones. Moreover, encouraged by the improvement in the luminescent properties after the introduction of the 4*H*-1,2,4-triazole ring, we found that the directly connected heterocycles could be the basis of very promising products. Therefore, we focused on modifying the methodology used to prepare analogous compounds containing 1,3,4-oxadiazole and 1,3,4-thiadiazole, so as to introduce the triazole ring instead [27]. This study was planned to make it possible not only to obtain new, unknown compounds, but also to analyze the influence of their structure on their absorption and emission properties.

## 2. Results

### 2.1. Synthesis

**Scheme 2:**
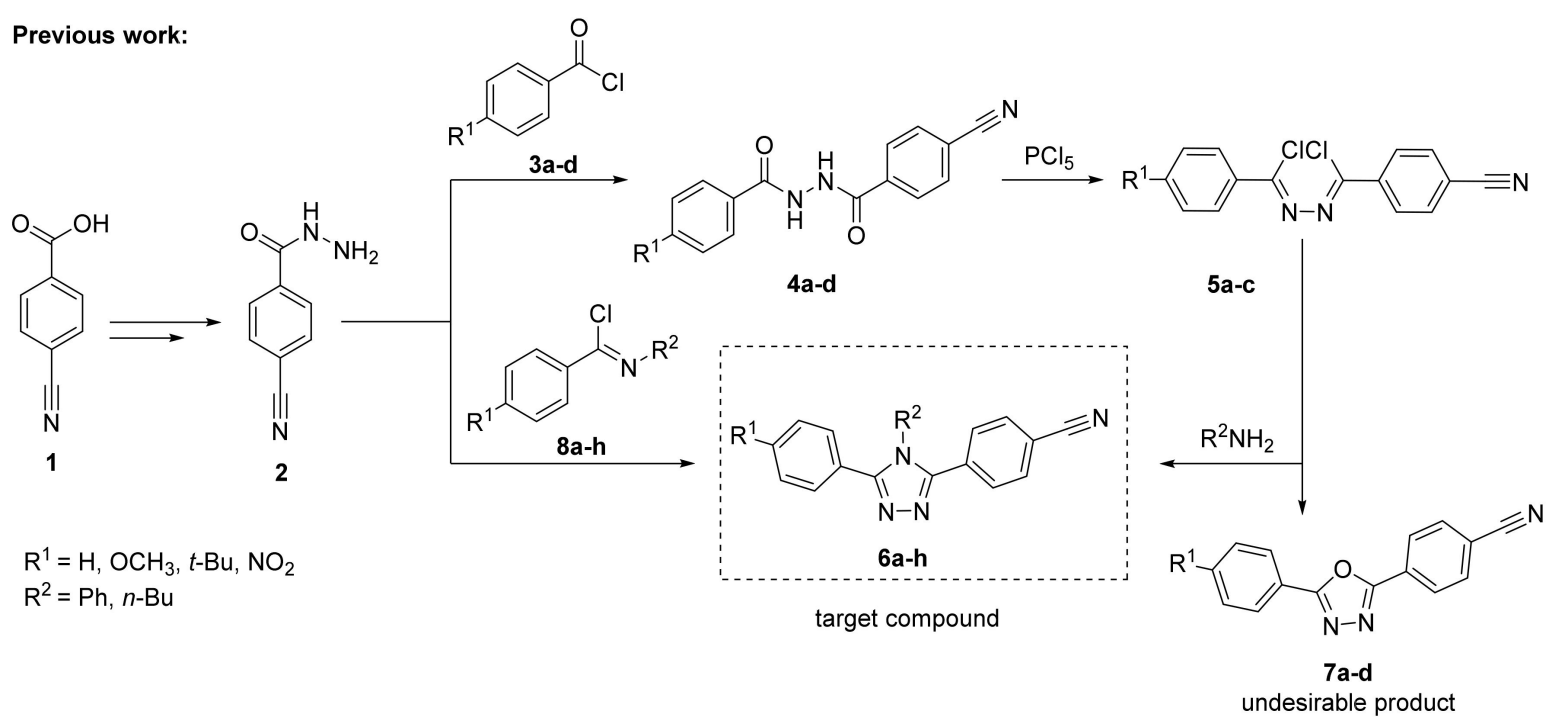
Synthesis of precursors containing a 4*H*-1,2,4-triazole ring (**6a**–**h**) [26].

As already mentioned in previous studies, we obtained a series of symmetric *s*-tetrazine derivatives conjugated via a 1,4-phenylene linker with a 4*H*-1,2,4-triazole ring. For this purpose, it was necessary to prepare appropriate precursors for the Pinner reaction, i.e., carbonitriles containing a five-membered ring (Scheme 2, **6a**–**h**). Initially, from the commercially available 4-cyanobenzoic acid (**1**), we obtained the hydrazide (**2**) in a two-step reaction sequence. The original assumption was to treat it with acid chlorides (**3a**–**d**) in order to obtain diacyl derivatives (**4a**–**d**), and then convert them into the corresponding imidoyl chlorides (**5a**–**d**), which, under the influence of amines, would be cyclized to the assumed products (**6a**–**h**). This approach, however, turned out to be very troublesome due to the formation of the undesirable products **7a**–**d**. This prompted us to change the reaction path by synthesizing other imidoyl chlorides (**8a**–**h**) from the corresponding amides. These intermediates were treated with hydrazide (**3**), resulting in the target precursors (**6a**–**h**) in satisfactory yields [26].

**Scheme 3:**
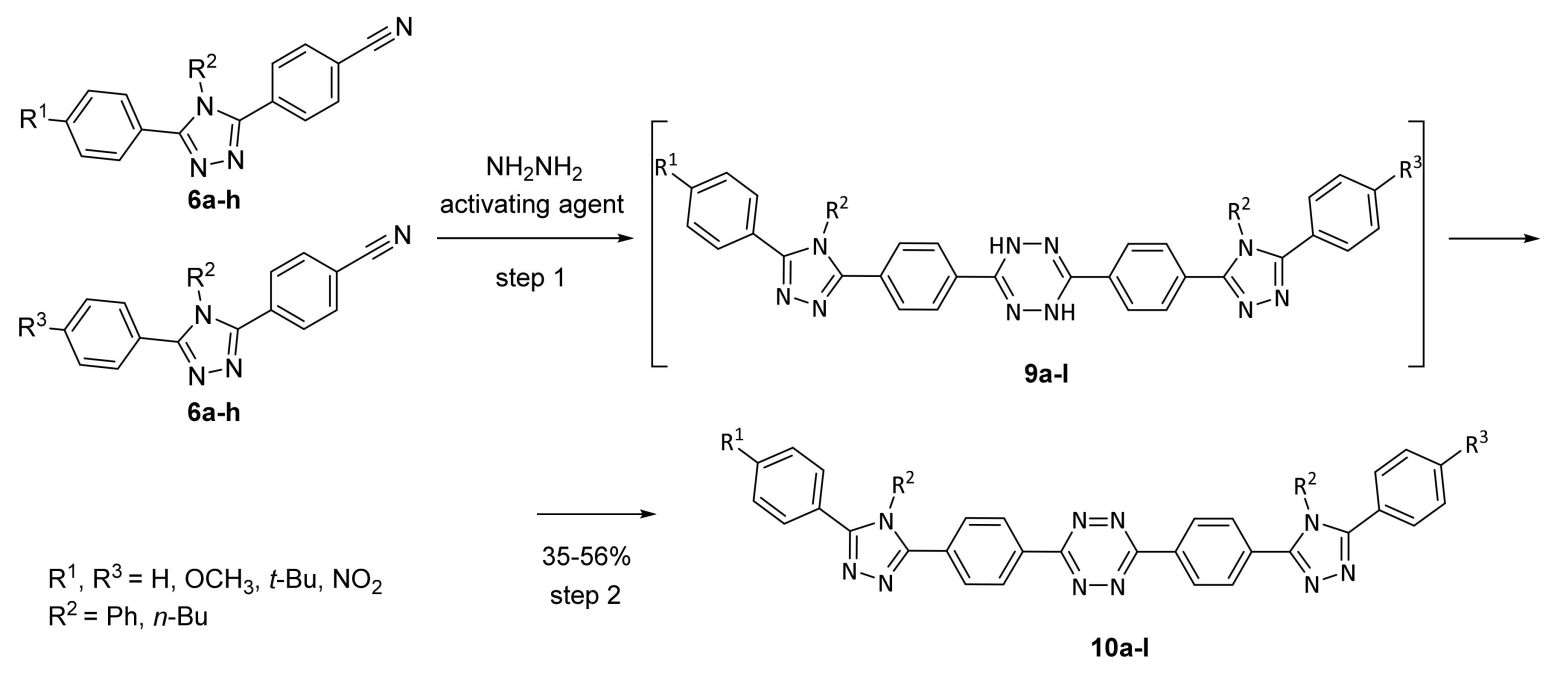
Synthesis of *s*-tetrazine derivatives conjugated via a 1,4-phenylene linker with a 4*H*-1,2,4-triazole ring (**10a**–**l**). Reaction conditions: step 1: two precursors (**6a**–**h**, 0.5 mmol of each compound), activating agent (zinc trifluoromethanesulfonate (0.009 g, 5 mol%) or sulfur (0.02 g, 125 mol%), ethanol (25 mL), hydrazine hydrate (hydrazine 64%,0.1 mL), reflux 12 h; step 2: methanol (10 mL), hydrogen peroxide (solution 34.5–36.5%,11 mL), rt, 24 h.

The presence of the carbonitrile moiety allows the formation of a second heterocycle, which is *s*-tetrazine. Under the conditions of the Pinner method, the treatment of the precursors **6a**–**h** with hydrazine hydrate, in the presence of an activating agent, leads to the formation of unoxidized derivatives of the assumed products **9a**–**l**. One of the popular activating agents is sulfur, with the help of which we have successfully obtained symmetrical *s*-tetrazine derivatives connected via a 1,4-phenylene linker with a 4*H*-1,2,4-triazole ring, and extended systems containing 1,3,4-oxadiazole and 1,3,4-thiadiazole cores [24,25,26]. Therefore, we also began to research the synthesis of unsymmetrical compounds with the use of this methodology, which allowed us to obtain the product 10a with a yield of 42% (Entry 1, Table 1). In connection with literature reports on the possibility of improving this yield with the use of zinc catalysts [28,29], we attempted to repeat the described transformation with its participation and, as a result, the yield increased to 56% (Entry 2, Table 1). An analogous test was performed for derivatives containing an aliphatic chain attached to the triazole nitrogen atom, instead of an aromatic ring (**10g**). Again, the yield improved from 35% to 50% (Entries 8 and 9, Table 1). These results were an important reason to modify the previously used procedure. Such a modified approach resulted in obtaining a series of unsymmetrical systems containing both electron-donating and electron-withdrawing substituents in the terminal ring. Traces of two symmetrical products were also detected. As in the previous studies, the oxidation was carried out with hydrogen peroxide (Scheme 3).

**Scheme 4:**
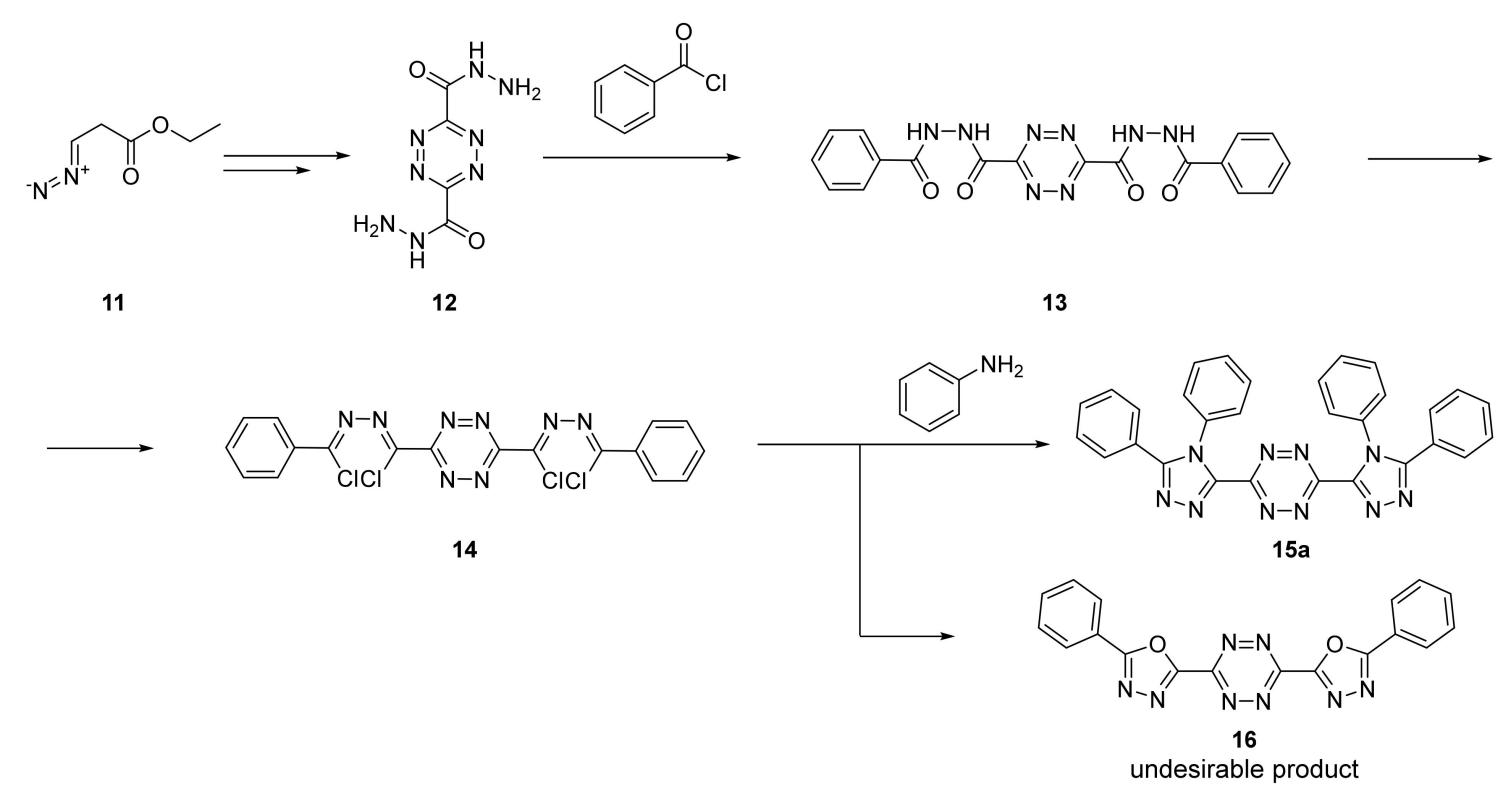
An attempt to synthesize *s*-tetrazine derivatives directly conjugated to the 4*H*-1,2,4-triazole ring.

The next step was the synthesis of products in which *s*-tetrazine is directly linked to the 4*H*-1,2,4-triazole ring. As part of our previous research, we had already obtained similar compounds containing 1,3,4-oxadiazole and 1,3,4-thiadiazole, but their synthesis required the use of microwave irradiation [27]. The methodology was based on the use of commercially available ethyl diazoacetate (**11**), which was transformed into a dihydrazide (**12**) in a sequence of several transformations (Scheme 4). The product was then treated with acid chlorides to prepare bisdiacyl derivatives (**13**). In this case, too, we intended to convert these compounds into imidoyl chlorides (**14**), which could then be cyclized to triazoles (**15a**) under the influence of amines. However, the high reactivity of such derivatives again caused serious difficulties. Despite the maximum shortening of the reaction times, which had a beneficial effect in previous studies, the observed undesirable derivatives of 1,3,4-oxadiazole (**16**) were predominantly formed. Additionally, isolation of the desired product from the reaction mixture was extremely problematic and, as a result, only traces of the target compound were obtained.

**Scheme 5:**
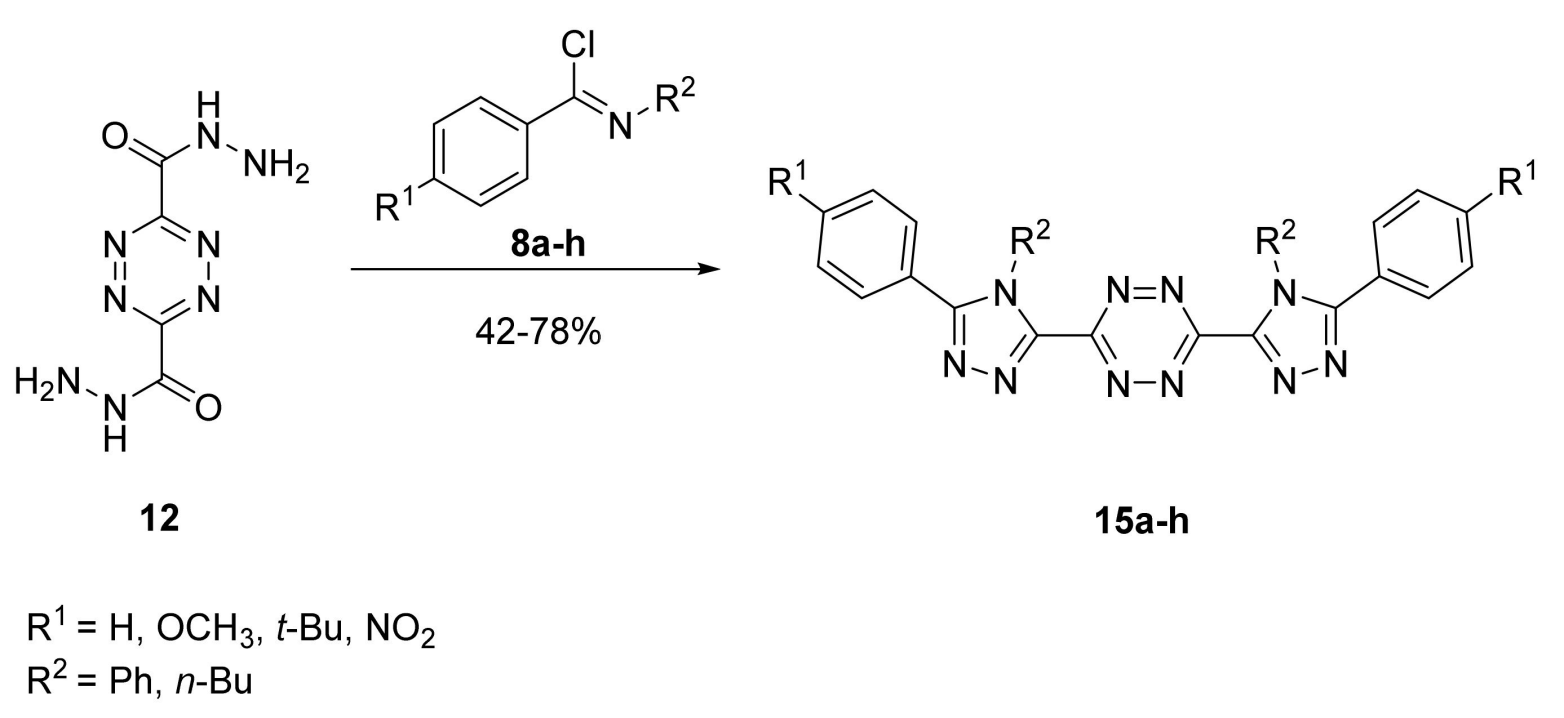
Synthesis of *s*-tetrazine derivatives directly conjugated to the 4*H*-1,2,4-triazole ring (**15a**–**h**). Reaction conditions: 1,2,4,5-tetrazine-3,6-dicarbohydrazide (**12**, 0.50 g, 2.5 mmol), imidoyl chloride (**8a**–**h**, 5.5 mmol), chloroform (20 mL), reflux, 24 h.

Based on the experience of obtaining triazole precursors for the Pinner reaction, where we encountered a similar problem, we decided to use an alternative methodology. For this purpose, the dihydrazide **12** was reacted with a range of imidoyl chlorides (**8a**–**h**) previously obtained from amides (Scheme 5). This approach was effective for both systems containing an aromatic ring (**15a**–**d**) and an aliphatic chain (**15e**–**h**) on the triazole nitrogen atom. In addition, derivatives containing both electron-donating and electron-withdrawing moieties attached to a terminal aromatic ring were obtained. Compared to the unsubstituted products, the electron-withdrawing nitro group showed a decreased yield (Entries 4 and 8, Table 2), while for the electron-donating groups (methoxy and *tert*-butyl) the yield was increased (Entries 2, 3, 6, 7, Table 2). The presence of an aliphatic chain also had a beneficial effect on the reaction yield (Entries 5–8, Table 2).

The structure of all the obtained intermediates and final products was confirmed by ^1^H- and ^13^C-NMR spectroscopy. Both in the case of systems containing a 1,4-phenylene linker, and with directly conjugated heterocycles, the ^13^C-NMR spectra were the most characteristic. The presence of the 4*H*-1,2,4-triazole ring was confirmed by signals above 140 ppm, and the presence of the *s*-tetrazine ring by signals above 160 ppm. The introduction of individual groups to the terminal aromatic ring conditioned the appearance of specific signals for the benzene carbon attached to them: above 160 ppm for the methoxy group, above 150 ppm for the *tert*-butyl group, and above 140 ppm for the nitro group. The lowest shifts corresponded to the carbon atoms of the aliphatic chain (13–45 ppm), the methoxy group (about 55 ppm), and the *tert*-butyl group (30–35 ppm). The ^1^H-NMR spectra mainly included aromatic signals. Additionally, the protons of the aliphatic chain (butyl) gave a series of signals in the range of 0.6–4.5 ppm, the methoxy group a peak around 3.8 ppm, and the *tert*-butyl group a peak around 1.3 ppm.

### 2.2. Luminescent Properties

UV-Vis and 3D fluorescence spectra were registered for compounds **10a**–**l** and **15a**–**h** (Figures S40–S64, Supplementary Materials). The fluorescence was completely quenched in the case of **15d** and **15h**, due to the presence of two NO_2_ groups in their structure. The rest of the compounds exhibited a maximum of one emission. The range of emission wavelengths is 375–412 nm for the **10a**–**l** series (Entries 1–12, Table 3) and 353–375 nm for the **15a**–**h** series (Entries 13–20, Table 3). It shows that the separation of fluorophore moieties by phenyl ring leads to a bathochromic shift of fluorescence. In the tetrazine and triazole derivatives, the n→π* transitions are a source of fluorescence [30,31,32,33]. The location of emission maximum (excitation wavelength—λ_ex_ and emission wavelength—λ_em_) is dependent on substituents R^1^, R^2^, and R^3^, which indicates that both tetrazine and triazole rings are involved in the orbitals from which the excitation occurs. The influence of substituents on λ_ex_ and λ_em_ is the same as in previously reported symmetrically substituted analogs of the **10a**–**l** series [26]. The R^2^ affects the λ_ex_, whereas R^1^ and R^3^ affect the λ_em_. The Ph substituent as R^2^ induces the bathochromic shift of λ_ex_ (Entries 1–6 and 13–16, Table 3) in comparison to *n*-Bu (Entries 7–12 and 17–20, Table 3, red color vs. blue color in Figure S65). In the case of the **15a**–**h** series, which consists of the symmetrically substituted compounds, the λ_em_ increases together with the rising electron-donating strength of R^1^ (H < *t*-Bu < OCH_3_), which is typical for tetrazine derivatives [34,35]. A partially similar relationship is observed in the unsymmetrically substituted **10a**–**l** series. Taking into account compounds with the same substituent as one of R^1^/R^3^, e.g., NO_2_, the λ_em_ shifts bathochromically in line with the electron-donating properties of the second R^1^/R^3^ substituent, i.e., H < *t*-Bu < OCH_3_. However, there are some exceptions to that rule in this series because, compared to compounds containing OCH_3_/*t*-Bu and OCH_3_/NO_2_ substituents (**10d** vs. **10e** and **10j** vs. **10k**, Entries 4, 5, 10 and 11, Table 3), those with NO_2_ (which is an electron-withdrawing group) unexpectedly possess a larger λ_em_. This shows that the changes in the electron density distribution induced by different substituents in unsymmetrically substituted compounds are difficult to predict, thus inferring their absorption-emission properties based only on a molecular structure can be misleading. The quantum yield (Φ) is directly related to the fluorescence intensity for the studied compounds (Figure S66). Generally, the compounds with Ph as R^2^ exhibit higher Φs than those with *n*-Bu, which is in agreement with previous findings [26]. However, most of the studied compounds are not efficient fluorescent materials, because their Φs do not exceed 0.3 (Table 3). The relatively favorable conjugation occurs only for three compounds, i.e., **10a**, **10b**, and **10d**. It shows that the direct coupling of tetrazine and triazole rings, as well as *n*-Bu as R^2^ and NO_2_ as R^1^/R^3^, decreases the population of fluorescent transitions. 

**Scheme 6:**
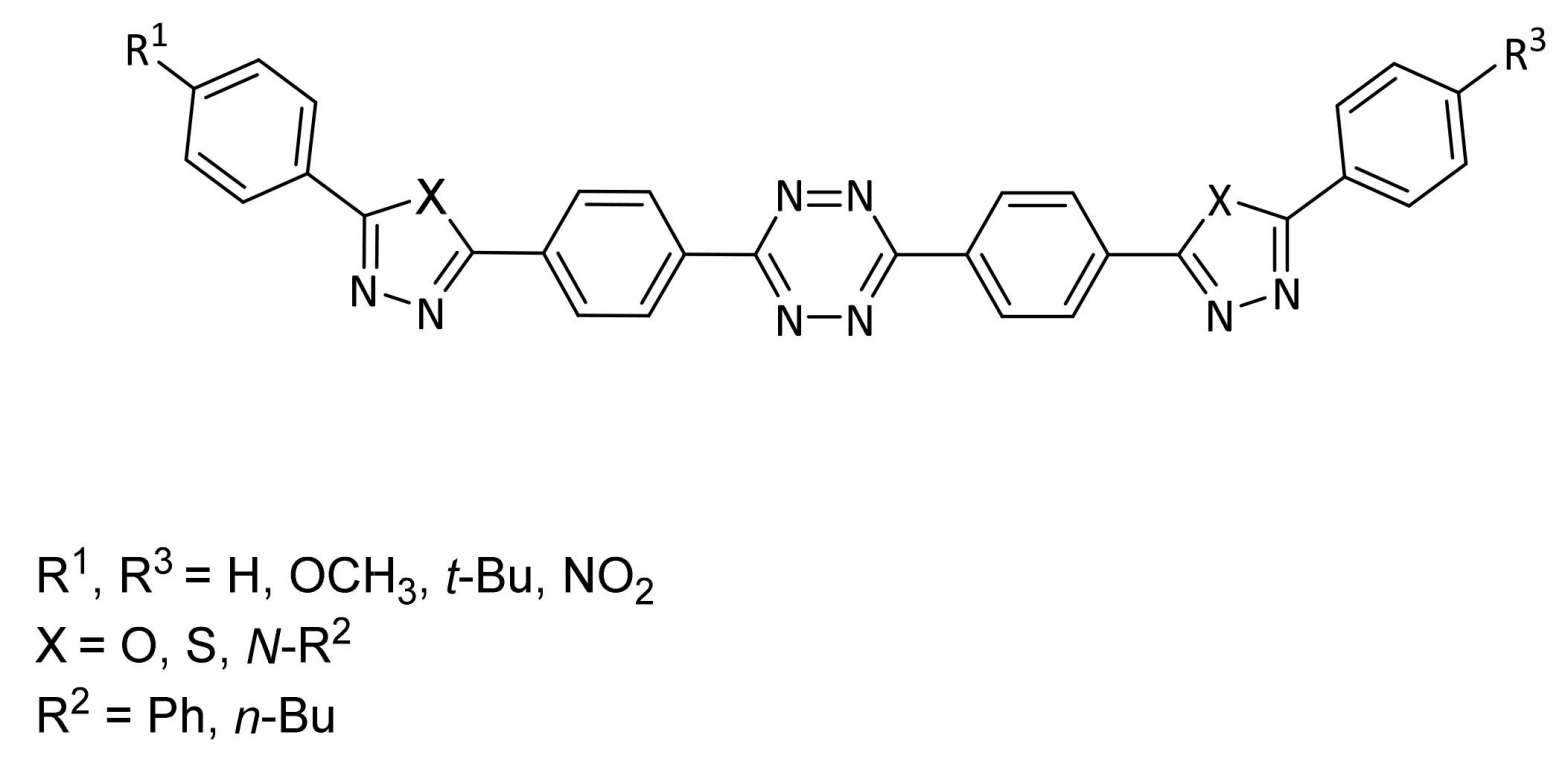
Structure of *s*-tetrazine derivatives conjugated via phenylene linkers with oxadiazole, thiadiazole, and triazole rings.

**Scheme 7:**
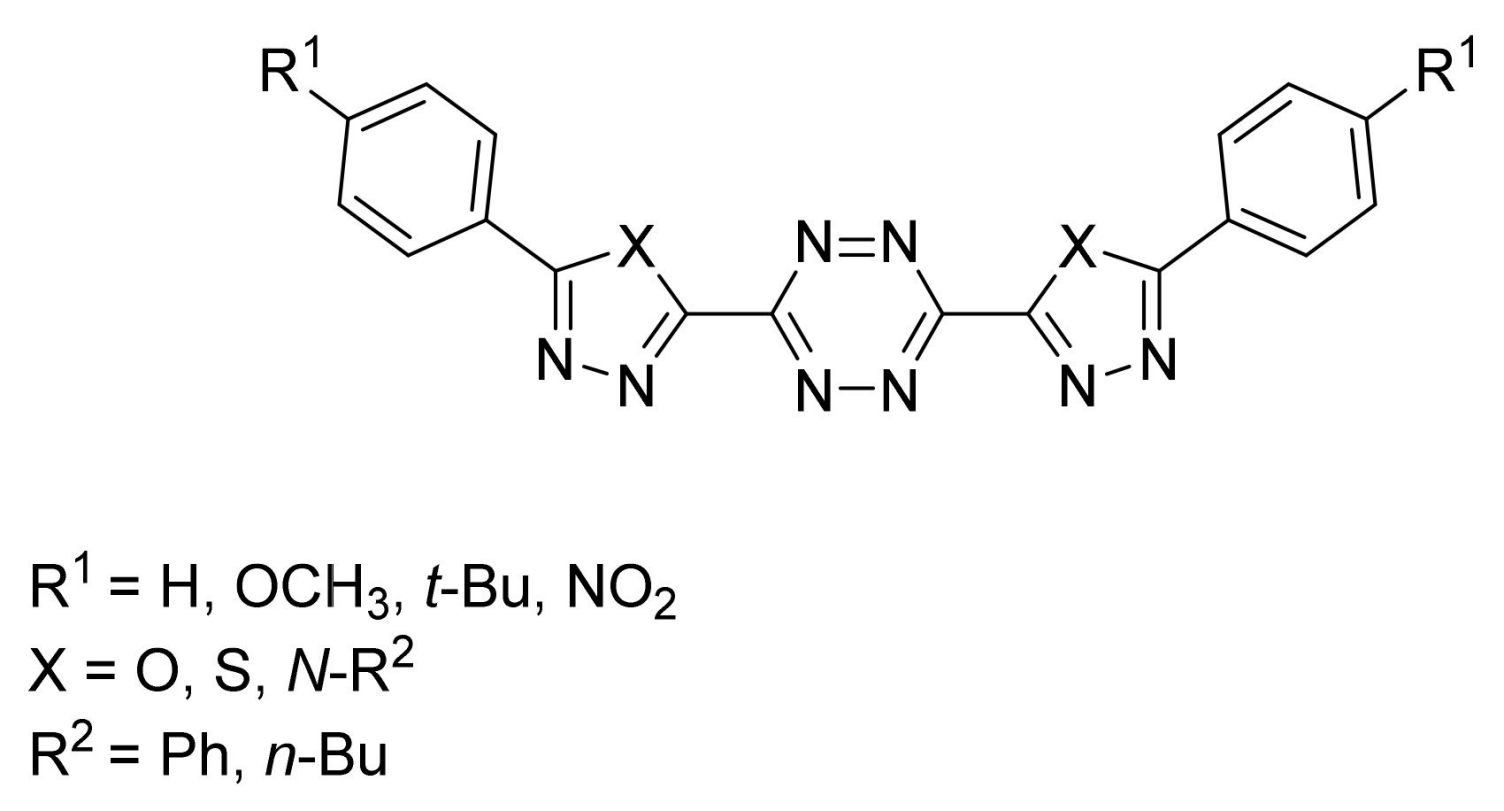
Structure of *s*-tetrazine derivatives directly conjugated with oxadiazole, thiadiazole, and triazole rings.

Summarizing the current and previous research on *s*-tetrazine derivatives in terms of their Φs, it can be stated that they are moderately efficient fluorescent materials. Most of the investigated tetrazine derivatives exhibit Φ no higher than 0.60, but there are some examples, which achieve Φ close to 1, which shows their great potential to use as functional materials, e.g., in optoelectronic applications. In the case of *s*-tetrazines conjugated via phenylene linkers with different 5-membered rings (Scheme 6, Table 4), the Φ changes approximately according to the following order, Triazole (R^2^ = *n*-Bu) < Oxadiazole ≤ Thiadiazole < Triazole (R^2^ = Ph). On the other hand, the analogical order for *s*-tetrazines directly conjugated with the same 5-membered rings is as follows, Triazole (R^2^ = *n*-Bu) < Triazole (R^2^ = Ph) < Oxadiazole < Thiadiazole (Scheme 7, Table 5). The greatest similarities are between oxadiazoles and thiadiazoles bearing *s*-tetrazine, due to small structural changes resulting from the replacement of oxygen with sulfur (atoms with similar electronic properties). Notably, the separation of tetrazine rings and triazole rings via phenylene linkers is more favorable for the fluorescence efficiency than the direct conjugation of them. This is in agreement with the study on the nature of the absorption–emission properties of tetrazine derivatives, which revealed that fluorescence is dependent on the character of HOMO and HOMO-1 orbitals [34]. Fluorescence occurs when the orbital involved in the excitation has a nonbonding n character, but if it is π orbital, the fluorescence is quenched. In this research, it was found that tetrazine derivatives directly conjugated with heteroatomic rings did not exhibit fluorescence, while diphenyl s-tetrazine was reported to be weakly fluorescent [34,39]. It showed that the conjugation with phenyl rings allows for the retention of the nonbonding n character of the excited orbitals, whereas the direct conjugation with heteroatomic rings changes its character to the π one.

## 3. Experimental Section

### 3.1. General Information

All reagents were purchased from commercial sources and used without further purification. Melting points were measured on a Stuart SMP3 melting point apparatus (Staffordshire, UK). NMR spectra were recorded at 25 °C on an Agilent 400-NMR spectrometer (Agilent Technologies, Waldbronn, Germany) at 400 MHz for ^1^H and 100 MHz for ^13^C, using CDCl_3_ or DMSO as the solvent and TMS as the internal standard. UV-Vis absorption and 3D fluorescence spectra were registered in dichloromethane solutions (c = 5 × 10^−6^ mol/dm^3^) with Jasco V-660 (Jasco Corporation, Tokyo, Japan) and Jasco F-6300 (Jasco Corporation, Tokyo, Japan) spectrometers, respectively. FT-IR spectra were measured between 4000 and 650 cm^−1^ on an FT-IR Nicolet 6700 apparatus (Thermo Fischer Scientific, Wesel, Germany) with a Smart iTR accessory. Elemental analyses were performed with a VarioELanalyser (Elementar UK Ltd., Stockport, UK). High-resolution mass spectra were obtained by means of a Waters ACQUITY UPLC/Xevo G2QT instrument (Waters Corporation, Milford, MA, USA). Thin-layer chromatography was performed on silica gel 60 F254 (Merck, Merck KGaA, Darmstadt, Germany) thin-layer chromatography plates using chloroform, chloroform/ethyl acetate (1:1 *v*/*v*), or chloroform/ethyl acetate (5:1 *v*/*v*) as the mobile phases.

### 3.2. Synthesis and Characterization

Compounds **6**, **8** and **12** were synthesized according to the literature [26,27].

#### 3.2.1. Synthesis of *s*-Tetrazine Derivatives Coupled via a 1,4-Phenylene Linkage with a 4*H*-1,2,4-Triazole Ring (**10a**–**l**)

Two of substrates (**6a**–**h**, 0.5 mmol of each compound) and zinc trifluoromethanesulfonate (0.009 g, 5 mol%) were suspended in ethanol (25 mL) and hydrazine hydrate (hydrazine 64%, 0.1 mL) was added dropwise. It was heated under reflux for 12 h, then filtered and evaporated on a rotary evaporator. The obtained crude intermediate (**9a**–**l**) was dissolved in methanol (10 mL), hydrogen peroxide was added (hydrogen peroxide solution 34.5−36.5%, 11 mL), and it was stirred at room temperature for 24 h. The resulting mixture was filtered and concentrated on a rotary evaporator. The crude product (**10a**–**l**) was purified by column chromatography using chloroform/ethyl acetate (1:1 *v*/*v*) as the mobile phases.

##### 3-(4-(4,5-Diphenyl-4*H*-1,2,4-triazol-3-yl)phenyl)-6-(4-(5-(4-methoxyphenyl)-4-phenyl-4*H*-1,2,4-triazol-3-yl)phenyl)-1,2,4,5-tetrazine (**10a**)

The product was obtained as yellow powder (0.20 g, 56%); m.p. 187–188 °C. UV (CH_2_Cl_2_) λ_max_ (log ε) 257 (4.76), 283 (4.64) nm; IR (ATR) ν_max_ 3064, 2947, 2232, 2187, 2141, 2129, 2098, 1696, 1683, 1609, 1565, 1533, 1494, 1472, 1445, 1256, 1179, 1077, 1019, 991, 972, 932, 848, 790, 772, 751, 730, 713, 699, 678 cm^–1^; ^1^H-NMR (400 MHz, CDCl_3_): δ 3.79 (s, 3H, OCH_3_), 6.81 (d, 2H, *J* = 8.0 Hz, Ar), 7.17–7.21 (m, 2H, Ar), 7.30–7.38 (m, 7H, Ar), 7.52–7.58 (m, 12H, Ar), 7.76 (d, 2H, *J* = 12.0 Hz, Ar), 8.11 (d, 2H, *J* = 8.0 Hz, Ar); ^13^C-NMR (100 MHz, CDCl_3_): δ 55.3, 113.4, 114.1, 117.6, 117.7, 118.1 126.2, 127.7, 127.7, 128.6, 128.8, 129.0, 129.9, 130.1, 130.2, 130.3, 130.3, 130.4, 130.4, 131.1, 132.2, 123.5, 134.2, 134.7, 152.7, 153.0, 155.3, 155.5, 161.0, 169.2, 171.1. Anal. calc. for C_43_H_30_N_10_O: C, 73.49; H, 4.30; N, 19.93. Found: C, 73. 46; H, 4.32; N, 19.91; HRMS (ESI): *m/z* calcd for C_43_H_30_N_10_O + H^+^: 703.2682; found: 703.2684.

##### 3-(4-(5-(4-(*tert*-Butyl)phenyl)-4-phenyl-4*H*-1,2,4-triazol-3-yl)phenyl)-6-(4-(4,5-diphenyl-4*H*-1,2,4-triazol-3-yl)phenyl)-1,2,4,5-tetrazine (**10b**)

The product was obtained as pink powder (0.19 g, 52%); m.p. 174–175 °C. UV (CH_2_Cl_2_) λ_max_ (log ε) 284 (4.64) nm; IR (ATR) ν_max_ 3062, 2964, 2868, 2232, 2167, 2155, 2028, 2007, 1966, 1695, 1610, 1527, 1494, 1473, 1435, 1362, 1305, 1269, 1201, 1181, 1156, 1108, 1078, 1019, 973, 932, 850, 837, 790, 773, 749, 730, 699 cm^–1^; ^1^H-NMR (400 MHz, CDCl_3_): δ 1.28 (s, 9H, C(CH_3_)_3_), 7.18 (t, 2H, *J* = 8.0 Hz, Ar), 7.29–7.39 (m, 9H, Ar), 7.48–7.57 (m, 14H, Ar), 7.78 (d, 2H, *J* = 8.0 Hz, Ar); ^13^C-NMR (100 MHz, CDCl_3_): δ 31.1, 34.8, 113.2, 113.3, 118.1, 118.1, 123.4, 125.5, 126.2, 127.6, 127.7, 128.3, 128.5, 128.8, 128.9, 129.0, 129.0, 130.0, 130.2, 130.2, 131.3, 132.2, 132.4, 134.7, 134.9, 152.9, 153.0, 153.3, 155.5, 155.5, 166.7, 167.6. Anal. calc. for C_46_H_36_N_10_: C, 75.80; H, 4.98; N, 19.22. Found: C, 75.81; H, 4.99; N, 19.20; HRMS (ESI): *m*/*z* calcd for C_46_H_36_N_10_ + H^+^: 729.3203; found: 729.3202.

##### 3-(4-(4,5-Diphenyl-4*H*-1,2,4-triazol-3-yl)phenyl)-6-(4-(5-(4-nitrophenyl)-4-phenyl-4*H*-1,2,4-triazol-3-yl)phenyl)-1,2,4,5-tetrazine (**10c**)

The product was obtained as yellow powder (0.18 g, 49%); m.p. 199–200 °C. UV (CH_2_Cl_2_) λ_max_ (log ε) 293 (4.71) nm; IR (ATR) ν_max_ 3053, 2232, 2172, 2142, 2129, 2003, 1965, 1698, 1608, 1550, 1515, 1494, 1468, 1446, 1428, 1406, 1337, 1317, 1277, 1202, 1181, 1152, 1108, 1078, 1018, 1002, 973, 933, 848, 790, 773, 760, 739, 713, 698, 685 cm^–1^; ^1^H-NMR (400 MHz, CDCl_3_): δ 7.22 (d, 2H, *J* = 8.0 Hz, Ar), 7.54–7.63 (m, 21H, Ar), 7.88 (d, 2H, *J* = 8.0 Hz, Ar), 8.16 (d, 2H, *J*= 8.0 Hz, Ar); ^13^C-NMR (100 MHz, CDCl_3_): δ 113.3, 113.8, 117.9, 118.1, 123.8, 126.4, 127.5, 127.7, 128.1, 128.5, 128.8, 129.0 129.0, 129.4, 130.1, 130.2, 130.4, 130.8, 131.3, 132.2, 132.3, 134.2, 134.8, 148.4, 153.0, 153.5, 153.8, 155.5, 163.6, 164.0. Anal. calc. for C_42_H_27_N_11_O_2_: C, 70.28; H, 3.79; N, 21.47. Found: C, 70.25; H, 3.77; N, 21.45; HRMS (ESI): *m*/*z* calcd for C_42_H_27_N_11_O_2_ + H^+^: 718.2427; found: 718.2425.

##### 3-(4-(5-(4-(*tert*-Butyl)phenyl)-4-phenyl-4*H*-1,2,4-triazol-3-yl)phenyl)-6-(4-(5-(4-methoxyphenyl)-4-phenyl-4*H*-1,2,4-triazol-3-yl)phenyl)-1,2,4,5-tetrazine (**10d**)

The product was obtained as pink powder (0.21 g, 56%); m.p. 159–160 °C. UV (CH_2_Cl_2_) λ_max_ (log ε) 238 (4.56), 287 (4,62) nm; IR (ATR) ν_max_ 3060, 2966, 2268, 2232, 2172, 2140, 2032, 2003, 1972, 1948, 1911, 1690, 1609, 1565,1531, 1496, 1475, 1459, 1434, 1362, 1305, 1254, 1200, 1175, 1156, 1099, 1076, 1020, 992, 972, 920, 851, 837, 789, 774, 749, 737, 714, 699 cm^–1^; ^1^H-NMR (400 MHz, CDCl_3_): δ 1.28 (s, 9H, C(CH_3_)_3_), 3.79 (s, 3H, OCH_3_), 6.80 (d, 2H, *J*= 8.0 Hz, Ar), 7.17 (m, 4H, Ar), 7.29–735 (m, 6H, Ar), 7.49–7.58 (m, 14H, Ar); ^13^C-NMR (100 MHz, CDCl_3_): δ 31.1, 34.8, 55.3, 113.2, 114.0, 118.1, 118.6, 120.2, 123.3, 125.5, 125.7, 127.7, 127.7, 128.3, 129.0, 129.9, 130.2, 130.2, 130.4, 131.3, 132.2, 132.4, 132.5, 132.9, 134.9, 152.9, 153.3, 154.6, 155.4, 155.5, 160.9, 164.0, 154.8. Anal. calc. for C_47_H_38_N_10_O: C, 74.39; H, 5.05; N, 18.46. Found: C, 74.38; H, 5.07; N, 18.44; HRMS (ESI): *m*/*z* calcd for C_47_H_38_N_10_O + H^+^: 759.3308; found: 759.3309.

##### 3-(4-(5-(4-Methoxyphenyl)-4-phenyl-4*H*-1,2,4-triazol-3-yl)phenyl)-6-(4-(5-(4-nitrophenyl)-4-phenyl-4*H*-1,2,4-triazol-3-yl)phenyl)-1,2,4,5-tetrazine (**10e**)

The product was obtained as orange powder (0.20 g, 54%); m.p. 189–190 °C. UV (CH_2_Cl_2_) λ_max_ (log ε) 303 (4.68) nm; IR (ATR) ν_max_ 3073, 2957, 2228, 2175, 2138, 2030, 2014, 1978, 1960, 1697, 1684, 1607, 1577, 1515, 1493, 1472, 1434, 1407, 1337, 1316, 1288, 1253, 1178, 1108, 1068, 1021, 992, 972, 848, 834, 784, 771, 752, 741, 698 cm^–1^; ^1^H-NMR (400 MHz, DMSO-d_6_): δ 3.75 (s, 3H, OCH_3_), 6.92 (d, 2H, *J* = 8.0 Hz, Ar), 7.33 (d, 2H, *J* = 12.0 Hz, Ar), 7.47–7.59 (m, 10H, Ar), 7.67 (d, 4H, *J* = 8.0 Hz, Ar), 7.88 (d, 4H, *J* = 8.0 Hz, Ar), 8.23 (d, 4H, *J* = 8.0 Hz, Ar); ^13^C-NMR (100 MHz, DMSO-d_6_): δ 55.2, 112.4, 113.95, 116.5, 118.1, 118.8, 123.7, 127.6, 128.1, 128.9, 129.1, 129.5, 129.7, 129.9, 130.0, 130.2, 130.4, 131.0, 131.5, 132.4, 132.5, 133.9, 134.6, 147.9, 152.6, 153.2, 153.6, 154.7, 160.3, 161.2, 163.3. Anal. calc. for C_43_H_29_N_11_O_3_: C, 69.07; H, 3.91; N, 20.60. Found: C, 69.09; H, 3.94; N, 20.58; HRMS (ESI): *m*/*z* calcd for C_43_H_29_N_11_O_3_ + H^+^: 748.2533; found: 748.2531.

##### 3-(4-(5-(4-(*tert*-Butyl)phenyl)-4-phenyl-4*H*-1,2,4-triazol-3-yl)phenyl)-6-(4-(5-(4-nitrophenyl)-4-phenyl-4*H*-1,2,4-triazol-3-yl)phenyl)-1,2,4,5-tetrazine (**10f**)

The product was obtained as orange powder (0.20 g, 52%); m.p. 124–125 °C. UV (CH_2_Cl_2_) λ_max_ (log ε) 292 (4.65) nm; IR (ATR) ν_max_ 3062, 2962, 2229, 2159, 2136, 2127, 2099, 2028, 1989, 1974, 1966, 1700, 1608, 1523, 1498, 1476, 1433, 1407, 1338, 1268, 1200, 1156, 1109, 1075, 1019, 972, 842, 787, 771, 752, 739, 729, 698 cm^–1^; ^1^H-NMR (400 MHz, CDCl_3_): δ 1.28 (s, 9H, C(CH_3_)_3_), 7.23 (d, 4H, *J* = 8.0 Hz, Ar), 7.32 (d, 2H, *J* = 8.0 Hz, Ar), 7.37 (d, 2H, *J* = 8.0 Hz, Ar), 7.48–7.64 (m, 16H, Ar), 8.16 (d, 2H, *J* = 8.0 Hz, Ar); ^13^C-NMR (100 MHz, CDCl_3_): δ 31.1, 34.9, 113.6, 113.8, 117.9, 118.0, 122.2, 123.8, 125.7, 127.5, 127.8, 128.6, 129.1, 129.2, 129.4, 130.5, 130.5, 130.6, 130.8, 130.9, 132.2, 132.3, 134.2, 134.3, 148.5, 152.9, 153.5, 153.8, 154.0, 155.2, 164.6, 166.0. Anal. calc. for C_46_H_35_N_11_O_2_: C, 71.40; H, 4.56; N, 19.91. Found: C, 71.42; H, 4.54; N, 19.90; HRMS (ESI): *m*/*z* calcd for C_46_H_35_N_11_O_2_ + H^+^: 774.3054; found: 774.3056.

##### 3-(4-(4-Butyl-5-(4-methoxyphenyl)-4*H*-1,2,4-triazol-3-yl)phenyl)-6-(4-(4-butyl-5-phenyl-4*H*-1,2,4-triazol-3-yl)phenyl)-1,2,4,5-tetrazine (**10g**)

The product was obtained as orange powder (0.16 g, 52%); m.p. 173–174 °C. UV (CH_2_Cl_2_) λ_max_ (log ε) 242 (4.52) nm; IR (ATR) ν_max_ 3103, 3075, 3053, 2329, 2231, 2175, 2138, 1945, 1695, 1682, 1607, 1566, 1504, 1403, 1317, 1294, 1243, 1176, 1130, 1112, 1052, 1024, 990, 869, 856, 844, 769, 751, 676 cm^–1^; ^1^H-NMR (400 MHz, CDCl_3_): δ 0.63–0.67 (m, 6H, CH_3_), 0.91–0.93 (m, 4H CH_2_), 1.36–1.43 (m, 4H, CH_2_), 3.87 (s, 3H, OCH_3_), 3.93–3.96 (m, 4H, CH_2_), 7.13 (d, 2H, *J* = 8.0 Hz, Ar), 7.73–7.78 (m, 9H, Ar), 7.90 (d, 2H, *J* = 8.0 Hz, Ar), 7.97–8.01 (m, 4H, Ar); ^13^C-NMR (100 MHz, CDCl_3_): δ 14.2, 14.3, 22.7, 22.8, 29.9, 30.4, 47.6, 47.6, 55.7, 113.7, 114.1, 117.7, 117.8, 128.2, 129.5, 129.7, 130.0, 130.2, 130.4, 131.7, 131.9, 132.3, 133.1, 134.3, 151.9, 152.1, 154.6, 155.4, 162.2, 164.1, 165.1. Anal. calc. for C_39_H_38_N_10_O: C, 70.67; H, 5.78; N, 21.13. Found: C, 70.69; H, 5.75; N, 21.11; HRMS (ESI): *m*/*z* calcd for C_39_H_38_N_10_O + H^+^: 663.3308; found: 663.3309.

##### 3-(4-(4-Butyl-5-(4-(*tert*-butyl)phenyl)-4*H*-1,2,4-triazol-3-yl)phenyl)-6-(4-(4-butyl-5-phenyl-4*H*-1,2,4-triazol-3-yl)phenyl)-1,2,4,5-tetrazine (**10h**)

The product was obtained as pink powder (0.16 g, 47%); m.p. 90–91 °C. UV (CH_2_Cl_2_) λ_max_ (log ε) 232 (4.51) nm; IR (ATR) ν_max_ 3316, 3067, 2958, 2932, 2867, 2231, 2193, 2170, 2134, 2034, 1978, 1959, 1721, 1637, 1578, 1541, 1490, 1465, 1395, 1364, 1308, 1275, 1249, 1221, 1178, 1154, 1109, 1074, 1018, 993, 946, 845, 803, 772, 694 cm^–1^; ^1^H-NMR (400 MHz, CDCl_3_): δ 0.63–0.67 (m, 6H, CH_3_), 0.92–0.95 (m, 4H CH_2_), 1.36–1.41 (m, 13H, CH_2_, C(CH_3_)_3_), 3.42–3.46 (m, 4H, CH_2_), 7.45–7.50 (m, 3H, Ar), 7.53 (d, 2H, *J* = 8.0 Hz, Ar), 7.60 (d, 2H, *J* = 8.0 Hz, Ar), 7.68–7.71 (m, 2H, Ar), 7.74–7.77 (m, 4H, Ar), 7.84 (d, 4H, *J* = 4.0 Hz, Ar); ^13^C-NMR (100 MHz, CDCl_3_): δ 13.1, 13.8, 19.3, 20.2, 31.2, 31.2, 21.7, 34.9, 44.8, 44.9, 113.9, 114.7, 118.0, 118.1, 124.0, 126.9, 127.8, 128.5, 129.4, 129.5, 129.9, 130.1, 131.3, 132.3, 132.5, 153.7, 153.8, 154.8, 155.3, 156.4, 167.5, 167.6. Anal. calc. for C_42_H_44_N_10_: C, 73.23; H, 6.44; N, 20.33. Found: C, 73.21; H, 6.46; N, 20.32; HRMS (ESI): *m*/*z* calcd for C_42_H_44_N_10_ + H^+^: 689.3829; found: 689.3827.

##### 3-(4-(4-Butyl-5-(4-nitrophenyl)-4*H*-1,2,4-triazol-3-yl)phenyl)-6-(4-(4-butyl-5-phenyl-4*H*-1,2,4-triazol-3-yl)phenyl)-1,2,4,5-tetrazine (**10i**)

The product was obtained as orange powder (0.15 g, 45%); m.p. 183–184 °C. UV (CH_2_Cl_2_) λ_max_ (log ε) 236 (4.34) nm; IR (ATR) ν_max_ 3307, 3067, 2958, 2928, 2872, 2231, 2173, 2136, 1697, 1637, 1602, 1578, 1526, 1490, 1466, 1346, 1307, 1248, 1178, 1108, 1074, 1016, 995, 853, 803, 771, 753, 694 cm^–1^; ^1^H-NMR (400 MHz, CDCl_3_): δ 0.94–0.97 (m, 6H, CH_3_), 1.36–1.46 (m, 4H CH_2_), 1.57–1.64 (m, 4H, CH_2_), 3.43–3.48 (m, 4H, CH_2_), 7.42 (t, 3H, *J* = 8.0 Hz, Ar), 7.47–7.49 (m, 2H, Ar), 7.74–7.76, m, 8H, Ar), 7.94 (d, 2H, *J* = 12.0 Hz, Ar), 8.24 (d, 2H, *J* = 8.0 Hz, Ar); ^13^C-NMR (100 MHz, CDCl_3_): δ 13.1, 13.8, 19.3, 20.2, 31.6, 31.7, 39.9, 40.2, 114.4, 114.8, 117.9, 119.0, 123.7, 126.8, 127.7, 128.2, 128.6, 129.5, 129.9, 130.4, 131.4, 132.5, 132.9, 149.5, 151.8, 152.0, 154.8, 155.1, 165.6, 167.7. Anal. calc. for C_38_H_35_N_11_O_2_: C, 67.34; H, 5.21; N, 22.73. Found: C, 67.33; H, 5.23; N, 22.74; HRMS (ESI): *m*/*z* calcd for C_38_H_35_N_11_O_2_ + H^+^: 678.3054; found: 678.3053.

##### 3-(4-(4-Butyl-5-(4-(*tert*-butyl)phenyl)-4*H*-1,2,4-triazol-3-yl)phenyl)-6-(4-(4-butyl-5-(4-methoxyphenyl)-4*H*-1,2,4-triazol-3-yl)phenyl)-1,2,4,5-tetrazine (**10j**)

The product was obtained as pink powder (0.18 g, 51%); m.p. 95–96 °C. UV (CH_2_Cl_2_) λ_max_ (log ε) 253 (4.58) nm; IR (ATR) ν_max_ 3265, 2957, 2871, 2229, 1632, 1607, 1544, 1504, 1464, 1396, 1365, 1307, 1253, 1222, 1176, 1113, 1031, 978, 918, 841, 772 cm^–1^; ^1^H-NMR (400 MHz, CDCl_3_): δ 0.65–0.67 (m, 6H, CH_3_), 0.92–0.96 (m, 4H CH_2_), 1.37–1.41 (m, 13H, CH_2_, C(CH_3_)_3_), 3.84 (s, 3H, OCH_3_), 3.86–3.89 (m, 4H, CH_2_), 7.04 (d, 2H, *J* = 8.0 Hz, Ar), 7.54 (d, 2H, *J* = 8.0 Hz, Ar), 7.61 (d, 2H, *J* = 4.0 Hz, Ar), 7.66–7.74 (m, 8H, Ar), 7.92 (d, 2H, *J* = 8.0 Hz, Ar); ^13^C-NMR (100 MHz, CDCl_3_): δ 13.2, 13.5, 19.2, 19.7, 31.2, 31.6, 31.8, 34.9, 44.8, 44.9, 55.4, 113.7, 114.6, 117.4, 118.2, 125.4, 126.0, 127.8, 128.6, 128.7, 129.4, 130.5, 131.5, 132.3, 133.0, 153.8, 154.7, 154.8, 155.2, 155.8, 162.0, 167.1, 167.5. Anal. calc. for C_43_H_46_N_10_O: C, 71.84; H, 6.45; N, 19.48. Found: C, 71.86; H, 6.44; N, 19.45; HRMS (ESI): *m*/*z* calcd for C_43_H_46_N_10_O + H^+^: 719.3934; found: 719.3935.

##### 3-(4-(4-Butyl-5-(4-methoxyphenyl)-4*H*-1,2,4-triazol-3-yl)phenyl)-6-(4-(4-butyl-5-(4-nitrophenyl)-4*H*-1,2,4-triazol-3-yl)phenyl)-1,2,4,5-tetrazine (**10k**)

The product was obtained as orange powder (0.17 g, 48%); m.p. 194–195 °C. UV (CH_2_Cl_2_) λ_max_ (log ε) 257 (4.51) nm; IR (ATR) ν_max_ 2964, 2842, 2228, 2128, 1601, 1578, 1519, 1437, 1308, 1256, 1171, 1105, 1050, 1033, 1021, 919, 837, 762, 747, 727, 686 cm^–1^; ^1^H-NMR (400 MHz, CDCl_3_): δ 0.65–0.69 (m, 6H, CH_3_), 0.94–0.99 (m, 4H CH_2_), 1.38–1.46 (m, 4H, CH_2_), 3.88–3.95 (m, 7H, CH_2_, OCH_3_), 6.92 (d, 2H, *J* = 8.0 Hz, Ar), 7.60 (d, 2H, *J* = 12.0 Hz, Ar), 7.72 (d, 2H, *J* = 8.0 Hz, Ar), 7.81–7.84 (m, 4H, Ar), 7.90–7.93 (m, 4H, Ar), 8.27–8.29 (m, 2H, Ar); ^13^C-NMR (100 MHz, CDCl_3_): δ 13.8, 13.8, 20.1, 20.2, 31.8, 31.9, 39.8, 40.2, 55.5, 113.7, 114.7, 117.5, 118.2, 124.1, 128.0, 128.6, 129.0, 129.4, 129.6, 130.3, 130.4, 130.7, 133.0, 148.0, 150.8, 150.8, 155.4, 156.2, 160.6, 165.1, 165.8. Anal. calc. for C_39_H_37_N_11_O_3_: C, 66.18; H, 5.27; N, 21.77. Found: C, 66.15; H, 5.29; N, 21.76; HRMS (ESI): *m*/*z* calcd for C_39_H_37_N_11_O_3_ + H^+^: 708.3159; found: 707.3157.

##### 3-(4-(4-Butyl-5-(4-(*tert*-butyl)phenyl)-4*H*-1,2,4-triazol-3-yl)phenyl)-6-(4-(4-butyl-5-(4-nitrophenyl)-4*H*-1,2,4-triazol-3-yl)phenyl)-1,2,4,5-tetrazine (**10l**)

The product was obtained as orange powder (0.17 g, 47%); m.p. 99–100 °C. UV (CH_2_Cl_2_) λ_max_ (log ε) 239 (4.57) nm; IR (ATR) ν_max_ 3265, 3067, 2958, 2933, 2867, 2230, 2149, 2132, 1636, 1611, 1526, 1501, 1477, 1464, 1395, 1364, 1346, 1304, 1286, 1269, 1200, 1154, 1111, 1016, 977, 841, 773, 751, 710, 693 cm^–1^; ^1^H-NMR (400 MHz, CDCl_3_): δ 0.64–0.69 (m, 6H, CH_3_), 1.02–1.05 (m, 4H CH_2_), 1.38–1.44 (m, 13H, CH_2_, C(CH_3_)_3_), 4.12–4.20 (m, 4H, CH_2_), 7.55 (d, 2H, *J* = 8.0 Hz, Ar), 7.61 (d, 2H, *J* = 8.0 Hz, Ar), 7.69–7.71 (m, 4H, Ar), 7.84–7.90 (m, 4H, Ar), 7.96 (d, 2H, *J* = 12.0 Hz, Ar), 8.25 (d, 2H, *J* = 12.0 Hz, Ar); ^13^C-NMR (100 MHz, CDCl_3_): δ 13.1, 13.8, 19.3, 20.2, 31.2, 31.6, 31.8, 35.0, 39.7, 40.2, 114.8, 114.9, 117.9, 118.1, 123.7, 125.5, 126.1, 126.6, 127.7, 128.2, 128.7, 129.3, 132.0, 132.4, 149.4, 151.2, 151.4, 154.8, 155.2, 155.7, 165.7, 167.5. Anal. calc. for C_42_H_43_N_11_O_2_: C, 68.74; H, 5.91; N, 20.99. Found: C, 68.75; H, 5.94; N, 20.97; HRMS (ESI): *m*/*z* calcd for C_42_H_43_N_11_O_2_ + H^+^: 734.3679; found: 734.3678.

#### 3.2.2. Synthesis of *s*-Tetrazine Derivatives Directly Conjugated with a 4*H*-1,2,4-Triazole Ring (**15a**–**h**)

The crude imidoyl chloride (**8a**–**h**, 5.5 mmol) and 1,2,4,5-tetrazine-3,6-dicarbohydrazide (12, 0.50 g, 2.5 mmol) were dissolved in chloroform (20 mL) and heated under reflux for 24 h. The mixture was then cooled to room temperature, filtered, and evaporated on a rotary evaporator. For systems containing an aromatic ring attached to a triazole nitrogen atom (**15a**–**d**) and compound **15h**, residue was washed with a small amount of cold ethanol to produce a pure product. For systems with an aliphatic chain, except compound **15h** (**15e**–**g**), a small amount of ethanol (5 mL) was added, filtered, and the filtrate was evaporated again to give the product as an oil.

##### 3,6-Bis(4,5-diphenyl-4*H*-1,2,4-triazol-3-yl)-1,2,4,5-tetrazine (**15a**)

The product was obtained as brown powder (0.59 g, 45%); m.p. 208–209 °C. UV (CH_2_Cl_2_) λ_max_ (log ε) 278 (4.48) nm; IR (ATR) ν_max_ 3352, 3061, 1967, 1685, 1596, 1541, 1497, 1466, 1444, 1385, 1317, 1261, 1188, 1074, 1017, 1000, 973, 931, 803, 781, 769, 730, 715, 692 cm^–1^; ^1^H-NMR (400 MHz, DMSO-d_6_): δ 7.39–7.45 (m, 12H, Ar), 7.50–7.56 (m, 8H, Ar); ^13^C-NMR (100 MHz, DMSO-d_6_): δ 120.3, 123.6, 127.6, 128.3, 128.4, 128.5, 128.6, 134.2, 154.5, 155.3, 164.1. Anal. calc. for C_30_H_20_N_10_: C, 69.22; H, 3.87; N, 26.91. Found: C, 69.25; H, 3.89; N, 26.90; HRMS (ESI): *m*/*z* calcd for C_30_H_20_N_10_ + H^+^: 521.1951; found: 521.1952.

##### 3,6-Bis(5-(4-methoxyphenyl)-4-phenyl-4*H*-1,2,4-triazol-3-yl)-1,2,4,5-tetrazine (**15b**)

The product was obtained as yellow powder (0.99 g, 68%); m.p. 217–218 °C. UV (CH_2_Cl_2_) λ_max_ (log ε) 256 (4.56) nm; IR (ATR) ν_max_ 3308, 3212, 2938, 2840, 2038, 1712, 1697, 1686, 1604, 1578, 1551, 1535, 1512, 1458, 1432, 1363, 1318, 1307, 1276, 1252, 1172, 1105, 1073, 1020, 916, 887, 851, 832, 795, 771, 741, 697 cm^–1^; ^1^H-NMR (400 MHz, DMSO-d_6_): δ 3.83 (s, 6H, OCH_3_), 7.04 (d, 4H, *J* = 8.0 Hz, Ar), 7.27–7.51 (m, 10H, Ar), 7.92 (d, 4H, *J* = 12.0 Hz, Ar); ^13^C-NMR (100 MHz, DMSO-d_6_): δ 55.5, 113.9, 120.3, 122.1, 127.7, 129.3, 130.1, 131.1, 153.8, 155.4, 163.0, 165.4. Anal. calc. for C_32_H_24_N_10_O_2_: C, 66.20; H, 4.17; N, 24.12. Found: C, 66.21; H, 4.19; N, 24.11; HRMS (ESI): *m/z* calcd for C_32_H_24_N_10_O_2_ + H^+^: 581.2162; found: 581.2160.

##### 3,6-Bis(5-(4-(*tert*-butyl)phenyl)-4-phenyl-4*H*-1,2,4-triazol-3-yl)-1,2,4,5-tetrazine(**15c**)

The product was obtained as orange powder (0.93 g, 59%); m.p. 198–199 °C. UV (CH_2_Cl_2_) λ_max_ (log ε) 276 (4.51) nm; IR (ATR) ν_max_ 3058, 2957, 2866, 2238, 2184, 2174, 2019, 1982, 1958, 1697, 1596, 1541, 1495, 1466, 1439, 1394, 1363, 1316, 1269, 1201, 1112, 1076, 1017, 963, 915, 841, 751, 731, 711, 692 cm^–1^; ^1^H-NMR (400 MHz, DMSO-d_6_): δ 1.22 (s, 18H, C(CH_3_)_3_), 7.14–7.19 (m, 4H, Ar), 7.24(d, 4H, *J* = 8.0 Hz, Ar), 7.47–7.53 (m, 10H, Ar); ^13^C-NMR (100 MHz, DMSO-d_6_): δ 29.8, 33.5, 119.2, 124.4, 126.8, 127.0, 127.2, 127.8, 132.9, 151.8, 153.0, 153.3, 163.1. Anal. calc. for C_38_H_36_N_10_: C, 72.13; H, 5.73; N, 22.14. Found: C, 72.11; H, 5.76; N, 22.12; HRMS (ESI): *m/z* calcd for C_38_H_36_N_10_ + H^+^: 633.3203; found: 633.3204.

##### 3,6-Bis(5-(4-nitrophenyl)-4-phenyl-4*H*-1,2,4-triazol-3-yl)-1,2,4,5-tetrazine (**15d**)

The product was obtained as orange powder (0.61 g, 40%); m.p. 172–173 °C. UV (CH_2_Cl_2_) λ_max_ (log ε) 298 (4.50) nm; IR (ATR) ν_max_ 3064, 2851, 2206, 2166, 2030, 1983, 1948, 1698, 1653, 1598, 1576, 1520, 1494, 1441, 1343, 1205, 1178, 1108, 1075, 1014, 965, 919, 853, 756, 708, 692 cm^–1^; ^1^H-NMR (400 MHz, DMSO-d_6_): δ 7.61–7.69 (m, 6H, Ar), 7.80(d, 4H, *J* = 8.0 Hz, Ar), 8.21 (d, 4H, *J* = 8.0 Hz, Ar), 8.37 (d, 4H, *J* = 8.0 Hz, Ar); ^13^C-NMR (100 MHz, DMSO-d_6_): δ 120.5, 123.5, 123.7, 124.1, 128.7, 129.2, 138.7, 149.1, 153.2, 153.4, 163.8. Anal. calc. for C_30_H_18_N_12_O_4_: C, 59.02; H, 2.97; N, 27.53. Found: C, 59.03; H, 2.99; N, 27.51; HRMS (ESI): *m*/*z* calcd for C_30_H_18_N_12_O_4_ + H^+^: 611.1652; found: 611.1650.

##### 3,6-Bis(4-butyl-5-phenyl-4*H*-1,2,4-triazol-3-yl)-1,2,4,5-tetrazine (**15e**)

The product was obtained as brown oil (0.67 g, 56%). UV (CH_2_Cl_2_) λ_max_ (log ε) 257 (4.45) nm; IR (ATR) ν_max_ 3264, 2957, 2932, 2873, 2212, 2165, 1636, 1541, 1491, 1449, 1378, 1308, 1220, 1157, 1113, 1074, 1026, 930, 802, 772, 694 cm^–1^; ^1^H-NMR (400 MHz, CDCl_3_): δ 0.92 (t, 6H, *J* = 8.0 Hz, CH_3_), 1.37 (sextet, 4H, *J* = 8.0 Hz, CH_2_), 1.63 (quintet, 4H, *J* = 8.0 Hz, CH_2_), 3.48 (t, 4H, *J* = 8.0 Hz, CH_2_), 7.39 (t, 4H, *J* = 8.0 Hz, Ar), 7.51 (t, 2H, *J* = 8.0 Hz, Ar), 7.85 (d, 4H, *J* = 8.0 Hz, Ar); ^13^C-NMR (100 MHz, CDCl_3_): δ 13.7, 20.1, 31.0, 41.3, 128.1, 128.6, 130.9, 132.8, 149.3, 153.8, 169.8. Anal. calc. for C_26_H_28_N_10_: C, 64.98; H, 5.87; N, 29.15. Found: C, 64.96; H, 5.88; N, 29.17; HRMS (ESI): *m*/*z* calcd for C_26_H_28_N_10_ + H^+^: 481.2577; found: 481.2578.

##### 3,6-Bis(4-butyl-5-(4-methoxyphenyl)-4*H*-1,2,4-triazol-3-yl)-1,2,4,5-tetrazine (**15f**)

The product was obtained as brown oil (1.05 g, 78%). UV (CH_2_Cl_2_) λ_max_ (log ε) 253 (4.57) nm; IR (ATR) ν_max_ 3299, 2957, 2932, 2872, 2213, 2151, 1697, 1608, 1577, 1541, 1506, 1464, 1440, 1365, 1295, 1251, 1176, 1112, 1027, 971, 837, 801, 770 cm^–1^; ^1^H-NMR (400 MHz, CDCl_3_): δ 0.95 (t, 6H, *J* = 8.0 Hz, CH_3_), 1.40 (sextet, 4H, *J* = 8.0 Hz, CH_2_), 1.59 (quintet, 4H, *J* = 8.0 Hz, CH_2_), 4.44 (t, 4H, *J* = 8.0 Hz, CH_2_), 3.84 (s, 6H, OCH_3_), 6.90 (d, 4H, *J* = 8.0 Hz, Ar), 7.74 (d, 4H, *J* = 12.0 Hz, Ar); ^13^C-NMR (100 MHz, CDCl_3_): δ 13.9, 20.3, 31.9, 39.8, 55.5, 113.8, 114.6, 128.7, 144.6, 155.0, 161.3, 167.1. Anal. calc. for C_28_H_32_N_10_O_2_: C, 62.21; H, 5.97; N, 25.91. Found: C, 62.24; H, 5.99; N, 25.90; HRMS (ESI): *m*/*z* calcd for C_28_H_32_N_10_O_2_ + H^+^: 541.2788; found: 541.2789.

##### 3,6-Bis(4-butyl-5-(4-(*tert*-butyl)phenyl)-4*H*-1,2,4-triazol-3-yl)-1,2,4,5-tetrazine (**15g**)

The product was obtained as brown oil (1.08 g, 73%). UV (CH_2_Cl_2_) λ_max_ (log ε) 259 (4.18) nm; IR (ATR) ν_max_ 3265, 2958, 2867, 2240, 2212, 2170, 2049, 1978, 1958, 1698, 1612, 1541, 1504, 1464, 1363, 1302, 1254, 1219, 1177, 1114, 1024, 924, 839, 771, 751 cm^–1^; ^1^H-NMR (400 MHz, CDCl_3_): δ 0.94 (t, 6H, *J* = 8.0 Hz, CH_3_), 1,35–1,38 (m, 22H, CH_2_, C(CH_3_)_3_), 1.58 (quintet, 4H, *J* = 8.0 Hz, CH_2_), 3.72 (t, 4H, *J* = 8.0 Hz, CH_2_), 7.42 (d, 4H, *J* = 8.0 Hz, Ar), 7.69 (d, 4H, *J* = 8.0 Hz, Ar); ^13^C-NMR (100 MHz, CDCl_3_): δ 13.9, 20.3, 31.3, 31.9, 35.1, 39.8, 125.5, 126.2, 126.8, 145.4, 154,8, 155.0, 167.5. Anal. calc. for C_34_H_44_N_10_: C, 68.89; H, 7.48; N, 23.63. Found: C, 68.87; H, 7.49; N, 23.65; HRMS (ESI): *m*/*z* calcd for C_34_H_44_N_10_ + H^+^: 593.3829; found: 593.3827.

##### 3,6-Bis(4-butyl-5-(4-nitrophenyl)-4*H*-1,2,4-triazol-3-yl)-1,2,4,5-tetrazine (**15h**)

The product was obtained as yellow powder (0.60 g, 42%); m.p. 103–104 °C. UV (CH_2_Cl_2_) λ_max_ (log ε) 269 (4.11) nm; IR (ATR) ν_max_ 3303, 3110, 2938, 2864, 2167, 2142, 2038, 2029, 2004, 1949, 1635, 1599, 1518, 1481, 1466, 1422, 1343, 1317, 1294, 1255, 1181, 1153, 1132, 1108, 1011, 973, 938, 868, 855, 841, 762, 723, 710, 691 cm^–1^; ^1^H-NMR (400 MHz, CDCl_3_): δ 0.97 (t, 6H, *J* = 8.0 Hz, CH_3_), 1.43 (sextet, 4H, *J* = 8.0 Hz, CH_2_), 1.63 (quintet, 4H, *J* = 8.0 Hz, CH_2_), 3.49 (t, 4H, *J* = 8.0 Hz, CH_2_), 7.93 (d, 4H, *J* = 12.0 Hz, Ar), 8.28 (d, 4H, *J* = 12.0 Hz, Ar); ^13^C-NMR (100 MHz, CDCl_3_): δ 13.9, 20.3, 31.7, 40.3, 123.9, 128.2, 130.2, 140.6, 145.1, 149.6, 165.6. Anal. calc. for C_26_H_26_N_12_O_4_: C, 54.73; H, 4.59; N, 29.46. Found: C, 54.71; H, 4.58; N, 29.47; HRMS (ESI): *m*/*z* calcd for C_26_H_26_N_12_O_4_ + H^+^: 571.2278; found: 571.2277.

## 4. Conclusions

Two effective methodologies for the synthesis of extended systems containing 1,2,4,5-tetrazine and 4*H*-1,2,4-triazole have been presented. The first methodology, comprising the Pinner reaction of carbonitriles bearing a 4*H*-1,2,4-triazole scaffold, is useful for obtaining unsymmetrical derivatives with heterocycles connected via a 1,4-phenylene linker. The second procedure, which makes use of imidoyl chloride and *s*-tetrazine-3,6-dicarbohydrazide, has proven to be successful for symmetrical systems with directly conjugated rings. In both cases, the approach leads to the desired products in satisfactory yields, regardless of the nature of the substituents attached to the terminal rings, as well as the type of groups on the triazole nitrogen atom. The obtained compounds exhibit mainly violet luminescence in CH_2_Cl_2_ solution. Their absorption–emission properties are directly related to the compound structure. The spectroscopic investigation revealed the dependency between the electron-donating strength of substituents and the emission wavelength, as well as the relationship between the quantum yield and the separation or direct conjunction of fluorophore moieties (tetrazine and triazole rings).

## Figures and Tables

**Table 1:** The yield of the reaction for the preparation of *s*-tetrazine derivatives conjugated via a 1,4-phenylene linker with a 4*H*-1,2,4-triazole ring (**10a–l**).

Entry	Product	R^1^	R^3^	R^2^	Activating Agent	Yield [%]
1	**10a**	H	OCH_3_	Ph	S	42
2					Zn(CF_3_SO_3_)_2_	56
3	**10b**	H	*t*-Bu	Ph	Zn(CF_3_SO_3_)_2_	52
4	**10c**	H	NO_2_	Ph	Zn(CF_3_SO_3_)_2_	49
5	**10d**	OCH_3_	*t*-Bu	Ph	Zn(CF_3_SO_3_)_2_	56
6	**10e**	OCH_3_	NO_2_	Ph	Zn(CF_3_SO_3_)_2_	54
7	**10f**	*t*-Bu	NO_2_	Ph	Zn(CF_3_SO_3_)_2_	52
8	**10g**	H	OCH_3_	*n*-Bu	S	35
9					Zn(CF_3_SO_3_)_2_	50
10	**10h**	H	*t*-Bu	*n*-Bu	Zn(CF_3_SO_3_)_2_	47
11	**10i**	H	NO_2_	*n*-Bu	Zn(CF_3_SO_3_)_2_	45
12	**10j**	OCH_3_	*t*-Bu	*n*-Bu	Zn(CF_3_SO_3_)_2_	51
13	**10k**	OCH_3_	NO_2_	*n*-Bu	Zn(CF_3_SO_3_)_2_	48
14	**10l**	*t*-Bu	NO_2_	*n*-Bu	Zn(CF_3_SO_3_)_2_	47

**Table 2:** The yield of the reaction for the preparation of *s*-tetrazine derivatives directly conjugated to the 4*H*-1,2,4-triazole ring (**15a**–**h**).

Entry	Product	R^1^	R^2^	Yield [%]
1	**15a**	H	Ph	45
2	**15b**	OCH_3_	Ph	68
3	**15c**	*t*-Bu	Ph	59
4	**15d**	NO_2_	Ph	40
5	**15e**	H	*n*-Bu	56
6	**15f**	OCH_3_	*n*-Bu	78
7	**15g**	*t*-Bu	*n*-Bu	73
8	**15h**	NO_2_	*n*-Bu	42

**Table 3:** Spectroscopic data for the studied *s*-tetrazine derivatives. λ_abs_—wavelength of absorption maximum directly preceding λ_em_. λ_ex_ and λ_em_—excitation and emission wavelength at global fluorescence maximum. Stokes shift was calculated as λ_em_ − λ_abs_. UV-Vis absorption and 3D fluorescence spectra were registered in dichloromethane solutions (c = 5 × 10^−6^ mol/dm^3^). The quantum yields Φ were determined according to the method described by Brouwer [36] by comparison with two standards: quinine sulphate (qn-SO_4_^2−^) [37] and *trans*,*trans*-1,4-diphenyl-1,3-butadiene (dpb) [38].

Entry	Compound	λ_abs_ (nm)	ε	λ_ex_ (nm)	λ_em_ (nm)	Stokes Shift (nm)	Φ	
			(mol^−1^ dm^3^ cm^−1^)				qn-SO_4_^2−^	dpb
1	**10a**	283	43,774	295	386	103	0.50	0.49
2	**10b**	284	43,560	300	382	98	0.70	0.69
3	**10c**	293	50,920	294	375	82	0.24	0.24
4	**10d**	287	41,880	302	391	104	0.67	0.66
5	**10e**	303	48,280	303	409	106	0.14	0.14
6	**10f**	292	44,760	299	384	92	0.29	0.28
7	**10g**	242	32,860	288	399	157	0.19	0.19
8	**10h**	232	32,680	291	378	146	0.20	0.20
9	**10i**	236	21,760	291	375	139	0.04	0.04
10	**10j**	253	38,120	288	396	143	0.05	0.05
11	**10k**	257	32,260	304	412	155	0.03	0.03
12	**10l**	239	36,940	296	386	147	0.22	0.21
13	**15a**	278	30,180	297	354	76	0.26	0.26
14	**15b**	256	36,160	309	375	119	0.26	0.25
15	**15c**	276	32,600	298	362	86	0.30	0.29
16	**15d**	298	31,300	-	-	-	-	-
17	**15e**	257	27,900	270	353	96	0.07	0.07
18	**15f**	253	37,100	284	373	120	0.21	0.20
19	**15g**	259	15,100	283	361	102	0.11	0.10
20	**15h**	269	12,800	-	-	-	-	-

**Table 4:** Comparison of the quantum yields of *s*-tetrazine derivatives conjugated via phenylene linkers with oxadiazole [24], thiadiazole [25], and triazole rings (symmetrically substituted from [26], and unsymmetrically substituted from current work).

Entry	R^1^	R^3^	Oxadiazole	Thiadiazole	Triazole R^2^ = Ph	Triazole R^2^ = *n*-Bu
1	H	H	0.09	0.46	0.69	0.59
2	OCH_3_	OCH_3_	0.39	0.60	>0.98	0.49
3	*t*-Bu	*t*-Bu	0.43	0.58	0.33	0.51
4	NO_2_	NO_2_	0.09	0.14	0.02	0.02
5	H	OCH_3_	0.41	0.44	0.50	0.19
6	H	*t*-Bu	0.51	0.40	0.70	0.20
7	H	NO_2_	0.57	0.26	0.24	0.04
8	OCH_3_	*t*-Bu	0.54	0.53	0.67	0.05
9	OCH_3_	NO_2_	0.39	0.38	0.14	0.03
10	*t*-Bu	NO_2_	0.05	0.26	0.29	0.22

**Table 5:** Comparison of the quantum yields of *s*-tetrazine derivatives directly conjugated with oxadiazole [27], thiadiazole [27], and triazole rings (current work).

Entry	R1	Oxadiazole	Thiadiazole	Triazole R^2^ = Ph	Triazole R^2^ = *n*-Bu
1	H	0.10	0.74	0.26	0.07
2	OCH_3_	>0.98	>0.98	0.26	0.21
3	*t*-Bu	*	*	0.30	0.11
4	NO_2_	0.08	0.50	-	-

* compound was not synthesized.

## Data Availability

Not applicable.

## Supplementary Materials

The following are available online at https://www.mdpi.com/article/10.3390/molecules27113642/s1. Copies of the ^1^H-NMR, ^13^C-NMR, UV-Vis and fluorescent spectra of the title compounds are available in the online Supplementary Materials.

## Sample Availability

Samples of the compounds **10a**–**l**, **15a**–**h** are available from the authors.
