# Supplementary material for: Synthesis and Luminescent Properties of s-Tetrazine Derivatives Conjugated with the 4H-1,2,4-Triazole Ring
Source: Molecules. 2022 Jun 6;27(11):3642. doi: 10.3390/molecules27113642 (PMC9181980; doi:10.3390/molecules27113642)
Supplement: Supplementary file 1 [file molecules-27-03642-s001.zip › molecules-1743952-supplementary.pdf]

Supplementary Materials

Synthesis and Luminescent Properties of *s*-Tetrazine Derivatives Conjugated with the 4*H*-1,2,4-Triazole Ring

Anna Maj [1], Agnieszka Kudelko [1,*] and Marcin Świątkowski [2]

[1] Department of Chemical Organic Technology and Petrochemistry, The Silesian University of Technology, Krzywoustego 4, PL-44100 Gliwice, Poland; anna.maj@polsl.pl
[2] Institute of General and Ecological Chemistry, Lodz University of Technology, Zeromskiego 116, PL-90924 Lodz, Poland; marcin.swiatkowski@p.lodz.pl
* Correspondence: agnieszka.kudelko@polsl.pl; Tel.: +48-32-237-1729

Table of contents

1. ^{1}H and ^{13}C NMR spectra 3

2. UV-Vis absorption spectra 23

3. 3D fluorescence spectra 25

4. Emission data scatter plots 35

5. High-resolution mass spectra (HRMS (ESI)) results 36

1. ^{1}H and ^{13}C NMR spectra

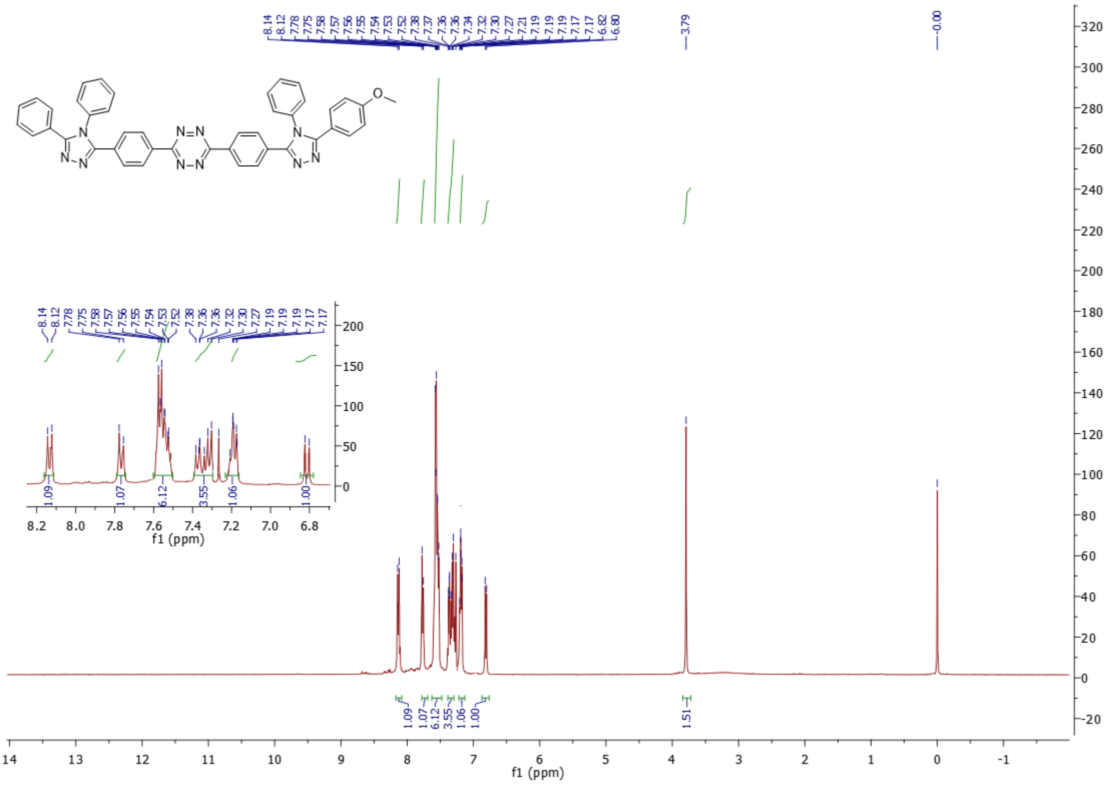

Figure S1. ^{1}H NMR spectra (400 MHz, $CDCl_3$)of3-(4-(4,5-diphenyl-4*H*-1,2,4-triazol-3-yl)phenyl)-6-(4-(5-(4-methoxyphenyl)-4-phenyl-4*H*-1,2,4-triazol-3-yl)phenyl)-1,2,4,5-tetrazine (**10a**)

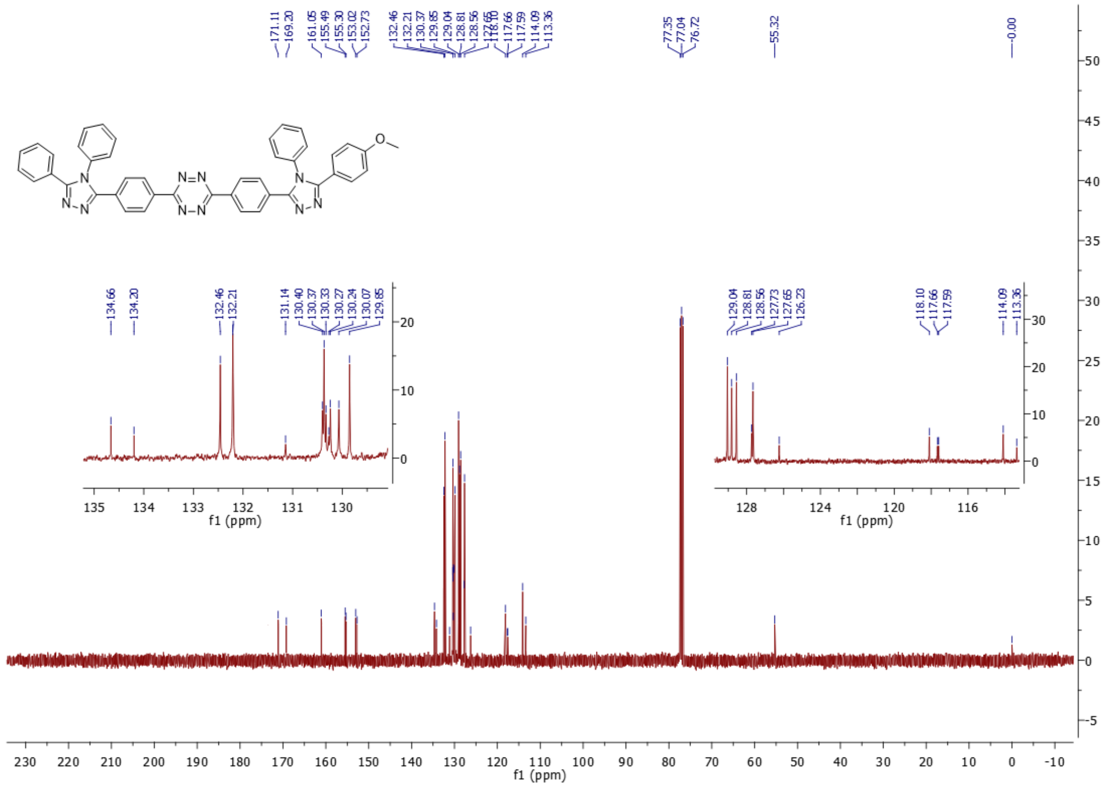

Figure S2. ^{13}C NMR spectra(100 MHz, $CDCl_3$)of3-(4-(4,5-diphenyl-4*H*-1,2,4-triazol-3-yl)phenyl)-6-(4-(5-(4-methoxyphenyl)-4-phenyl-4*H*-1,2,4-triazol-3-yl)phenyl)-1,2,4,5-tetrazine (**10a**)

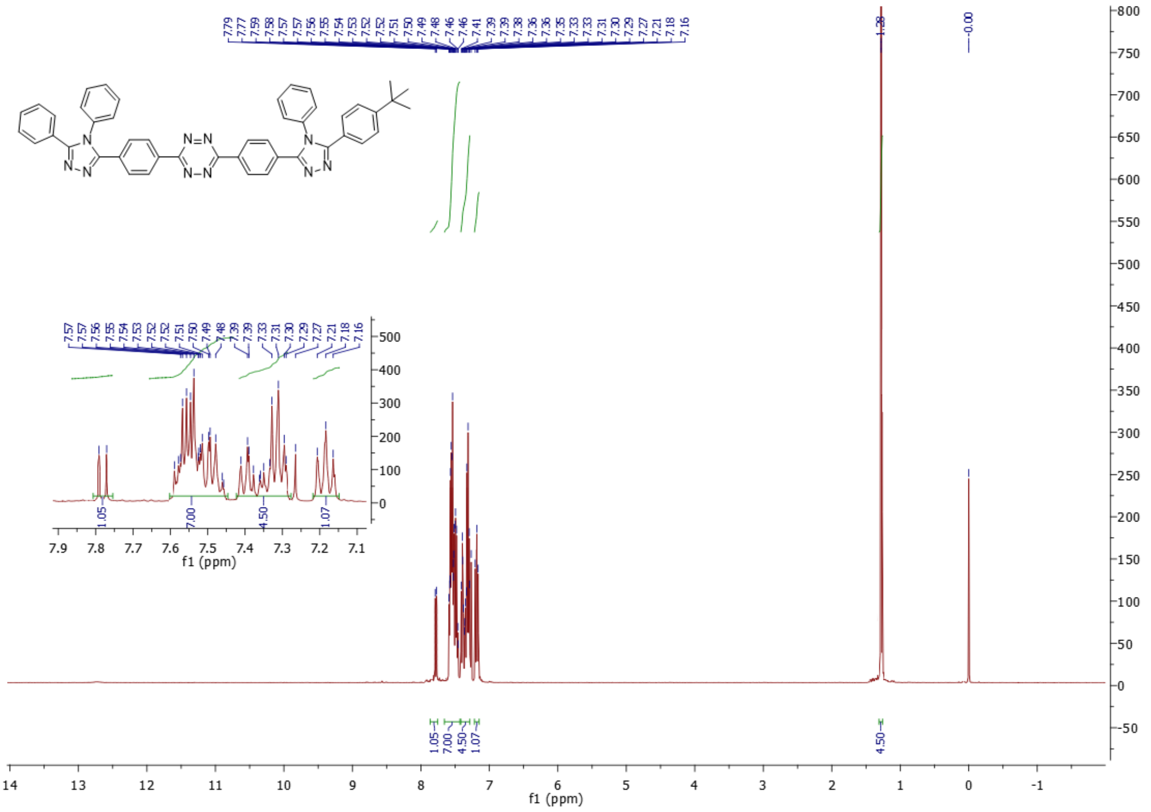

Figure S3. ^{1}H NMR spectra (400 MHz, $CDCl_3$)of3-(4-(5-(4-(*tert*-butyl)phenyl)-4-phenyl-4*H*-1,2,4-triazol-3-yl)phenyl)-6-(4-(4,5-diphenyl-4*H*-1,2,4-triazol-3-yl)phenyl)-1,2,4,5-tetrazine (**10b**)

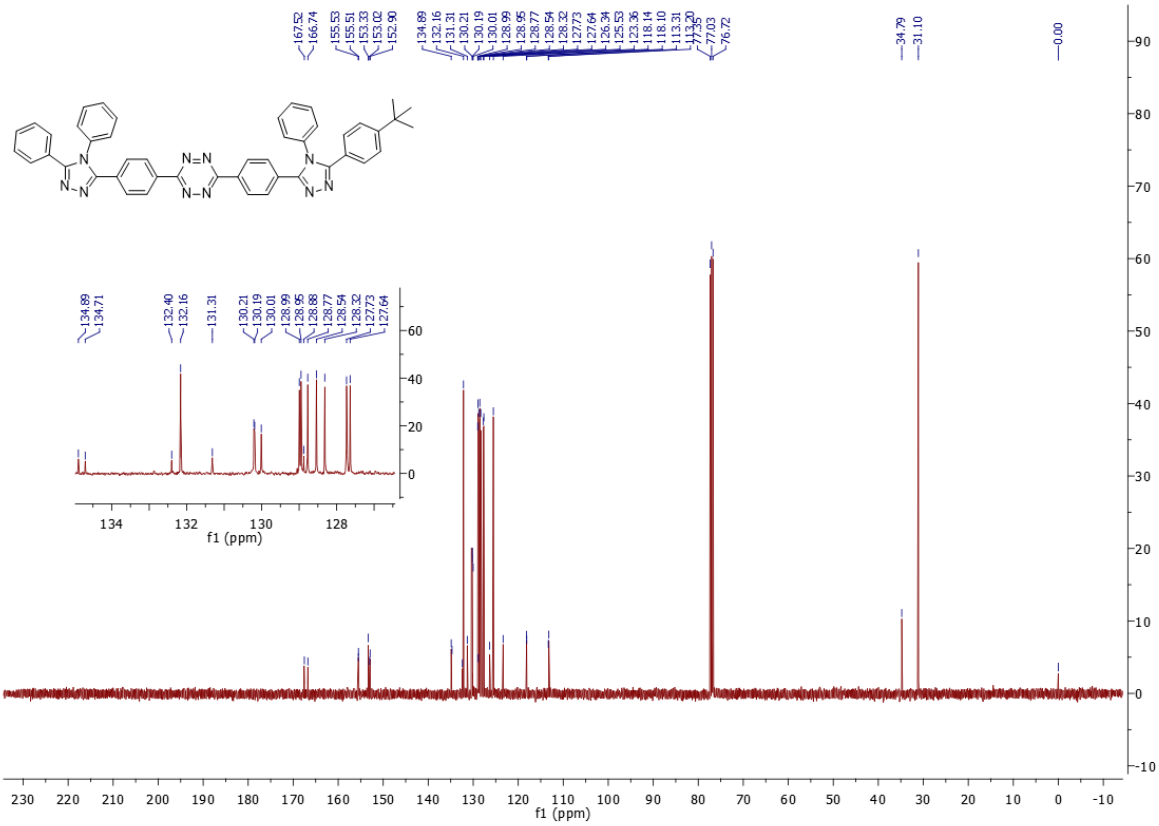

Figure S4. ^{13}C NMR spectra(100 MHz, $CDCl_3$)of3-(4-(5-(4-(*tert*-butyl)phenyl)-4-phenyl-4*H*-1,2,4-triazol-3-yl)phenyl)-6-(4-(4,5-diphenyl-4*H*-1,2,4-triazol-3-yl)phenyl)-1,2,4,5-tetrazine (**10b**)

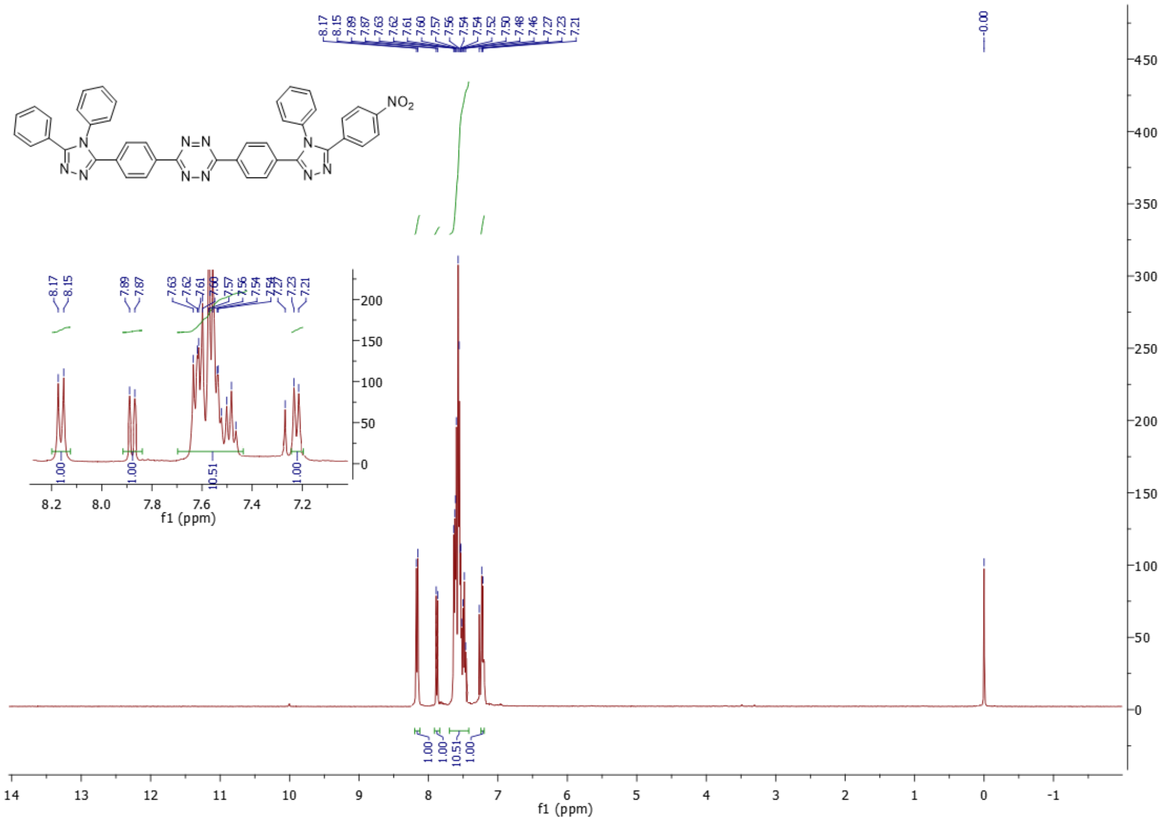

Figure S5. ^{1}H NMR spectra (400 MHz, $CDCl_3$) of 3-(4-(4,5-diphenyl-4*H*-1,2,4-triazol-3-yl)phenyl)-6-(4-(5-(4-nitrophenyl)-4-phenyl-4*H*-1,2,4-triazol-3-yl)phenyl)-1,2,4,5-tetrazine (**10c**)

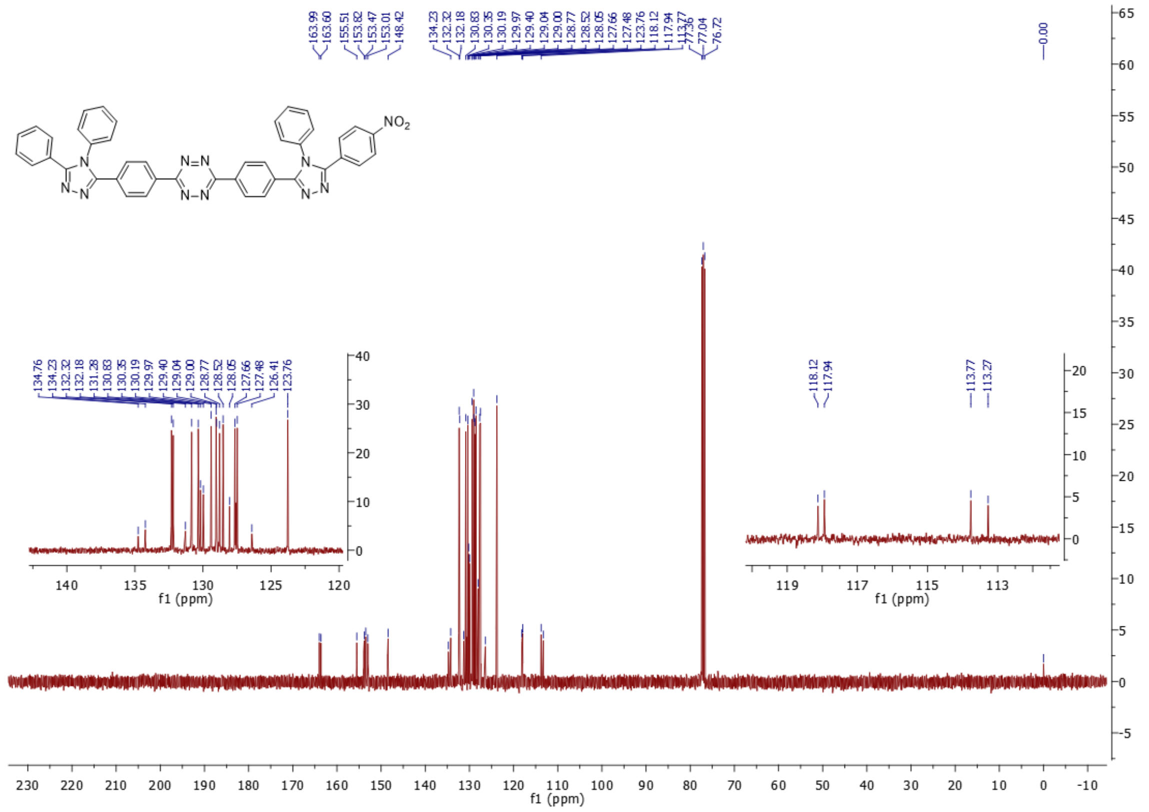

Figure S6. ^{13}C NMR spectra(100 MHz, $CDCl_3$)of 3-(4-(4,5-diphenyl-4*H*-1,2,4-triazol-3-yl)phenyl)-6-(4-(5-(4-nitrophenyl)-4-phenyl-4*H*-1,2,4-triazol-3-yl)phenyl)-1,2,4,5-tetrazine (**10c**)

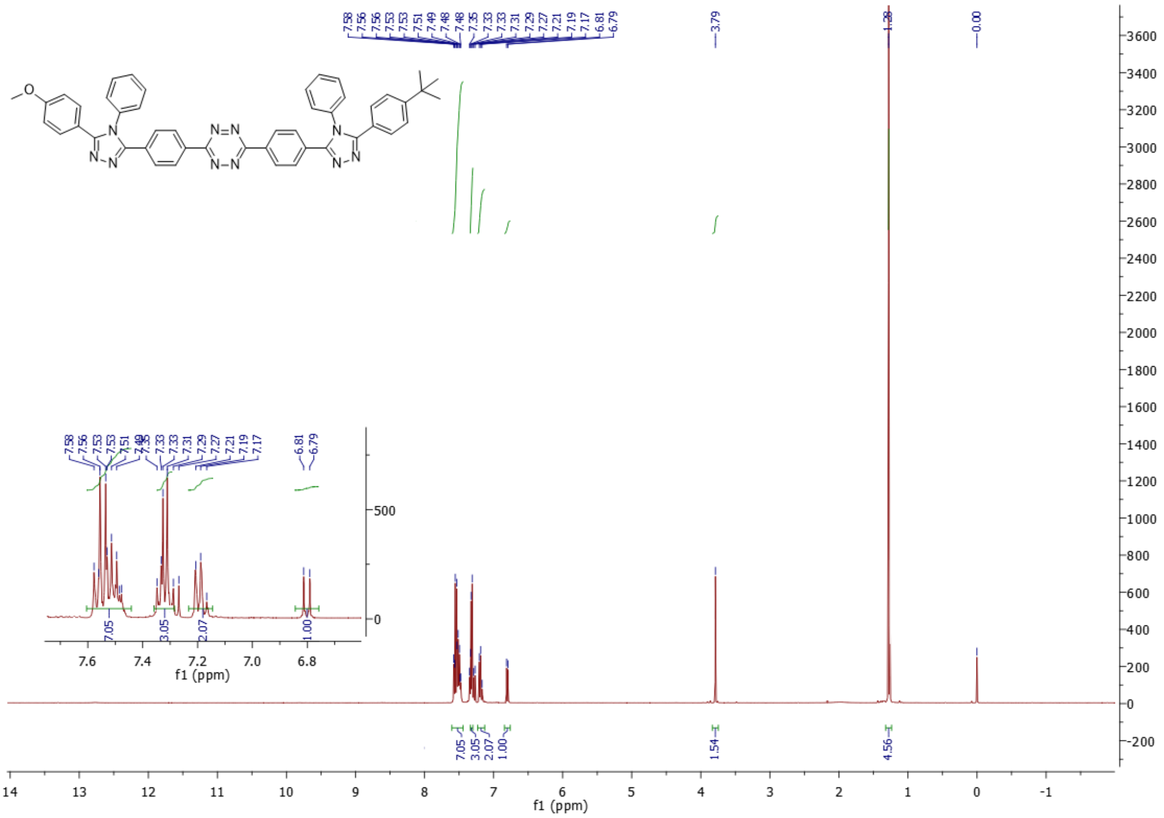

Figure S7. ^{1}H NMR spectra (400 MHz, $CDCl_3$) of 3-(4-(5-(4-(*tert*-butyl)phenyl)-4-phenyl-4*H*-1,2,4-triazol-3-yl)phenyl)-6-(4-(5-(4-methoxyphenyl)-4-phenyl-4*H*-1,2,4-triazol-3-yl)phenyl)-1,2,4,5-tetrazine (**10d**)

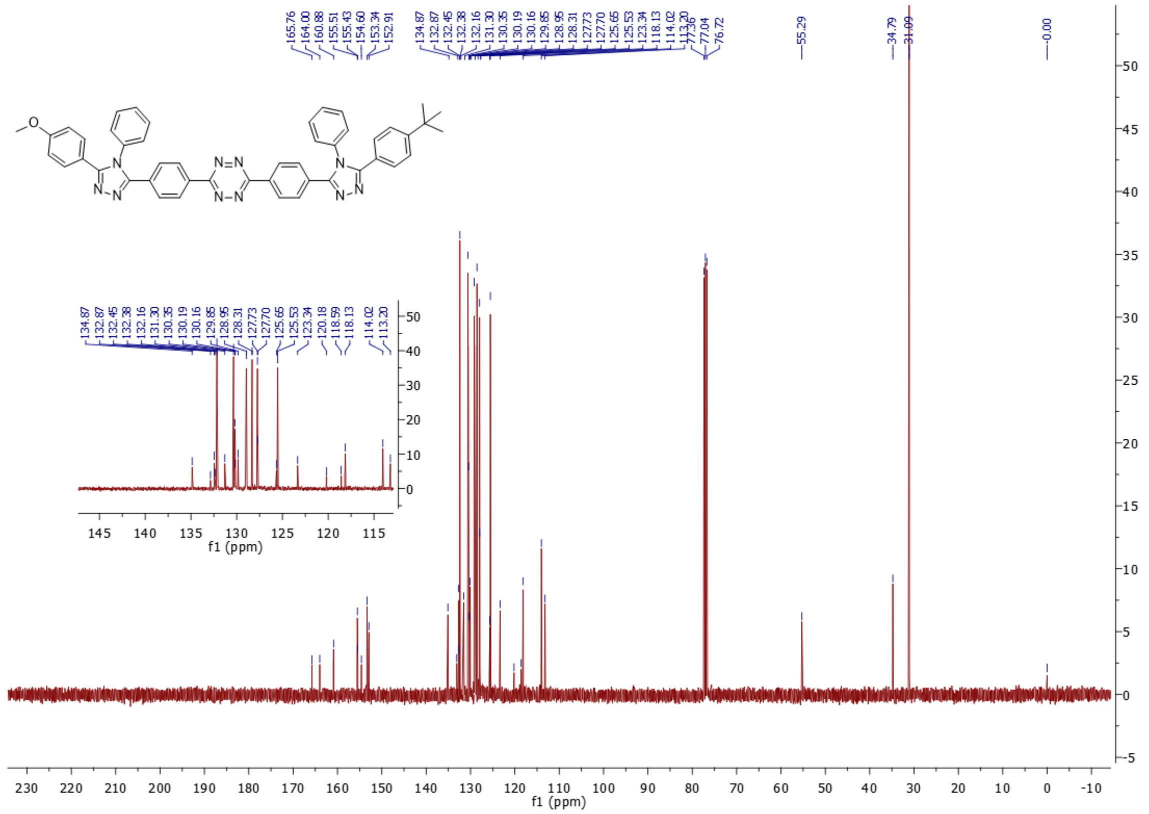

Figure S8. ^{13}C NMR spectra(100 MHz, $CDCl_3$)of 3-(4-(5-(4-(*tert*-butyl)phenyl)-4-phenyl-4*H*-1,2,4-triazol-3-yl)phenyl)-6-(4-(5-(4-methoxyphenyl)-4-phenyl-4*H*-1,2,4-triazol-3-yl)phenyl)-1,2,4,5-tetrazine (**10d**)

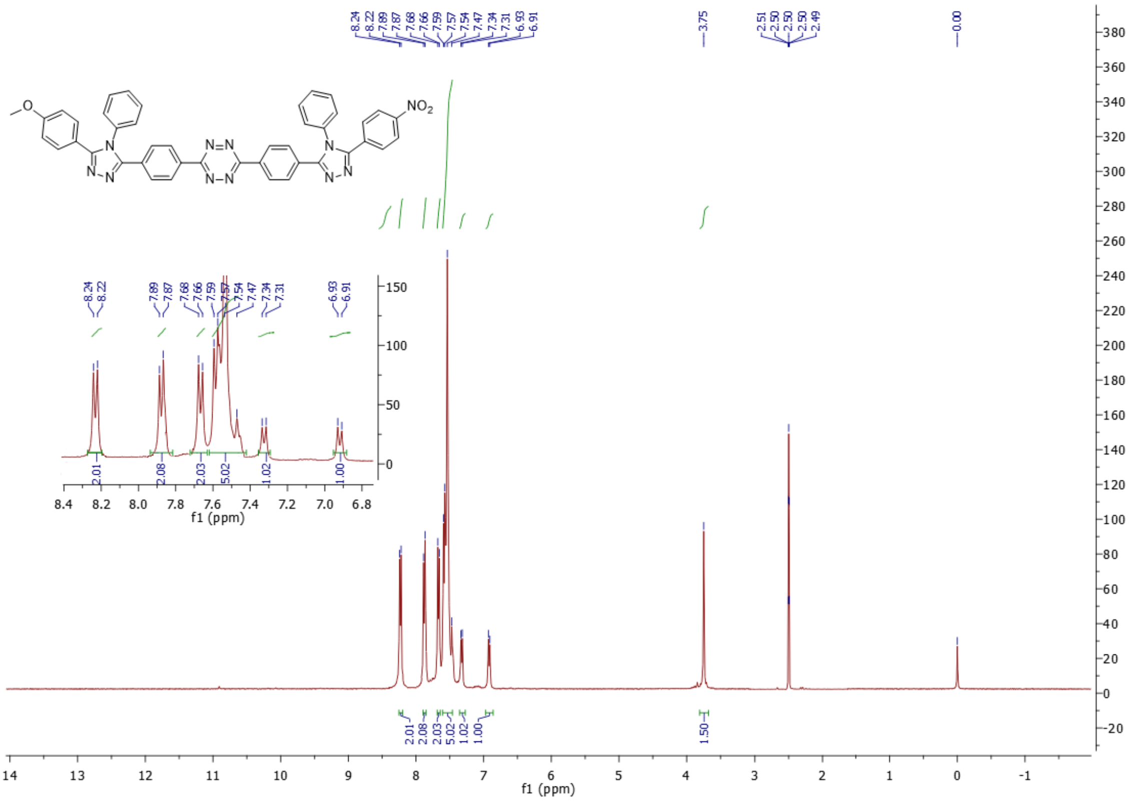

Figure S9. ^{1}H NMR spectra (400 MHz, DMSO-d_6) of 3-(4-(5-(4-methoxyphenyl)-4-phenyl-4*H*-1,2,4-triazol-3-yl)phenyl)-6-(4-(5-(4-nitrophenyl)-4-phenyl-4*H*-1,2,4-triazol-3-yl)phenyl)-1,2,4,5-tetrazine (**10e**)

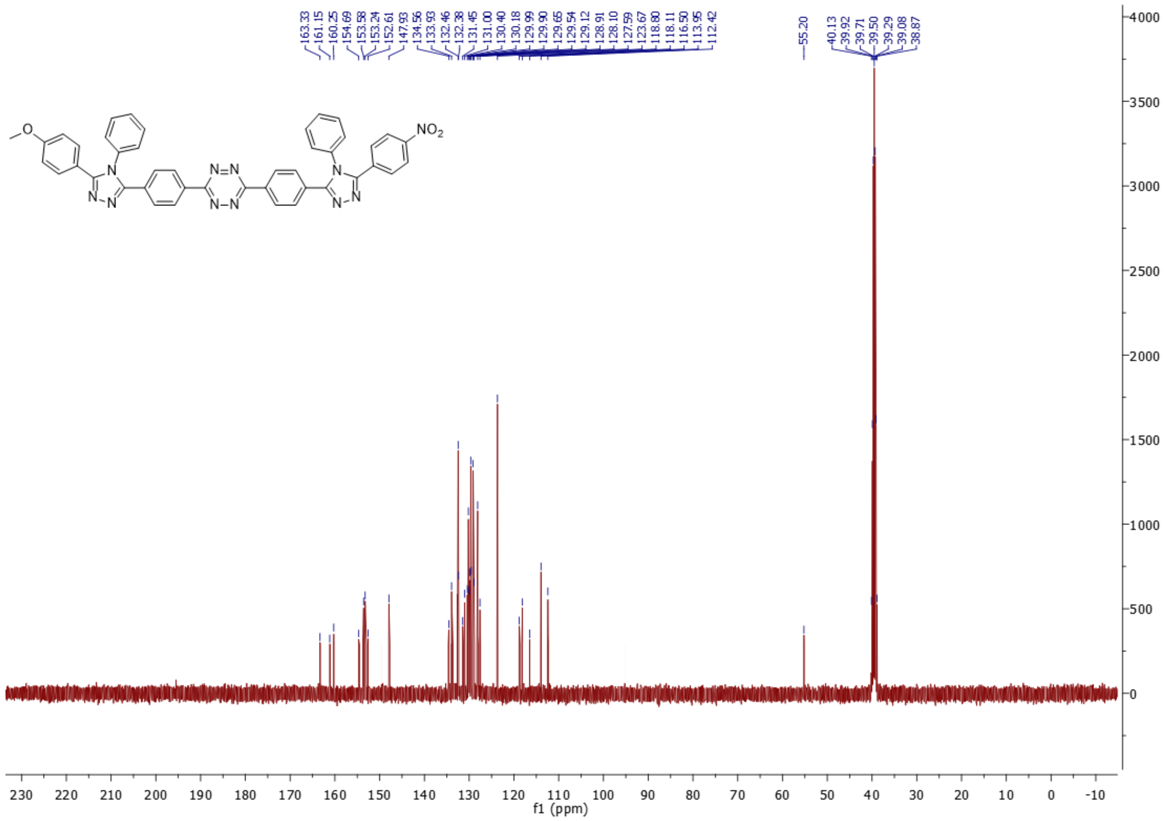

Figure S10. ^{13}C NMR spectra(100 MHz, DMSO-d_6) of 3-(4-(5-(4-methoxyphenyl)-4-phenyl-4*H*-1,2,4-triazol-3-yl)phenyl)-6-(4-(5-(4-nitrophenyl)-4-phenyl-4*H*-1,2,4-triazol-3-yl)phenyl)-1,2,4,5-tetrazine (**10e**)

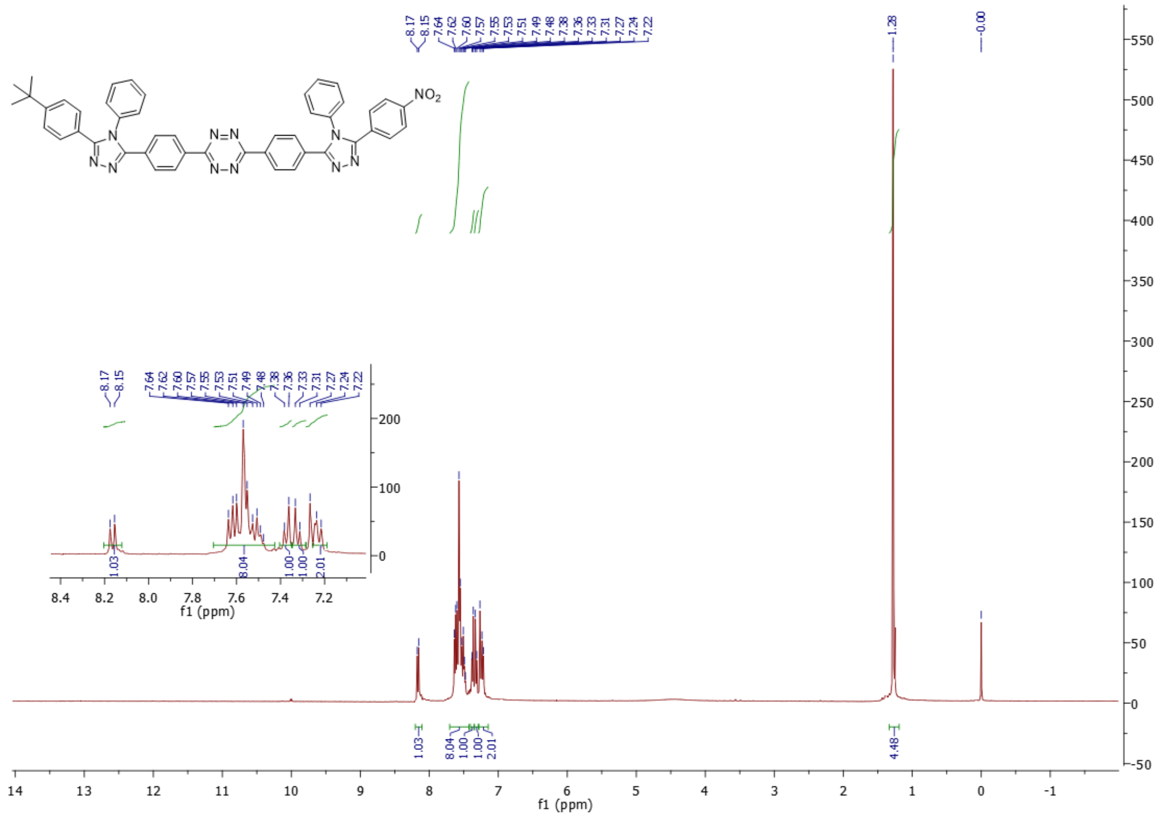

Figure S11. ^{1}H NMR spectra (400 MHz, $CDCl_3$) of 3-(4-(5-(4-(*tert*-butyl)phenyl)-4-phenyl-4*H*-1,2,4-triazol-3-yl)phenyl)-6-(4-(5-(4-nitrophenyl)-4-phenyl-4*H*-1,2,4-triazol-3-yl)phenyl)-1,2,4,5-tetrazine (**10f**)

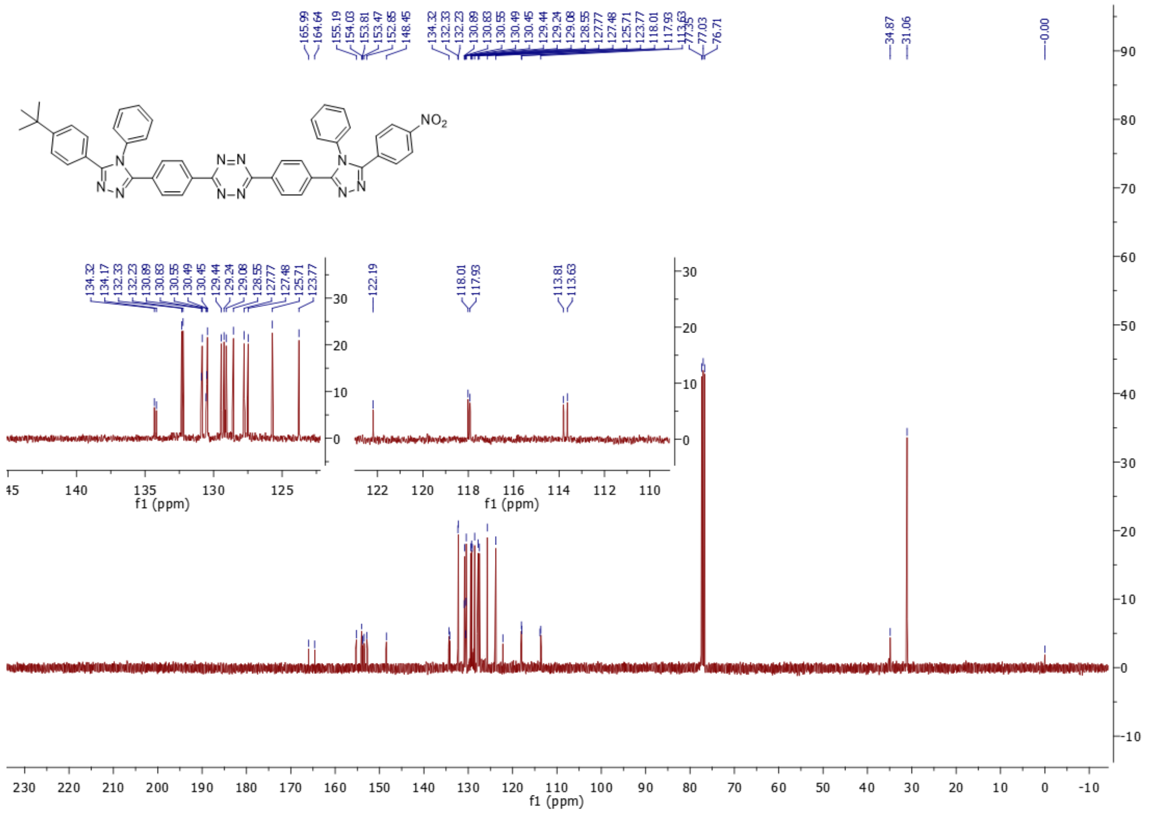

Figure S12. ^{13}C NMR spectra(100 MHz, $CDCl_3$)of 3-(4-(5-(4-(*tert*-butyl)phenyl)-4-phenyl-4*H*-1,2,4-triazol-3-yl)phenyl)-6-(4-(5-(4-nitrophenyl)-4-phenyl-4*H*-1,2,4-triazol-3-yl)phenyl)-1,2,4,5-tetrazine (**10f**)

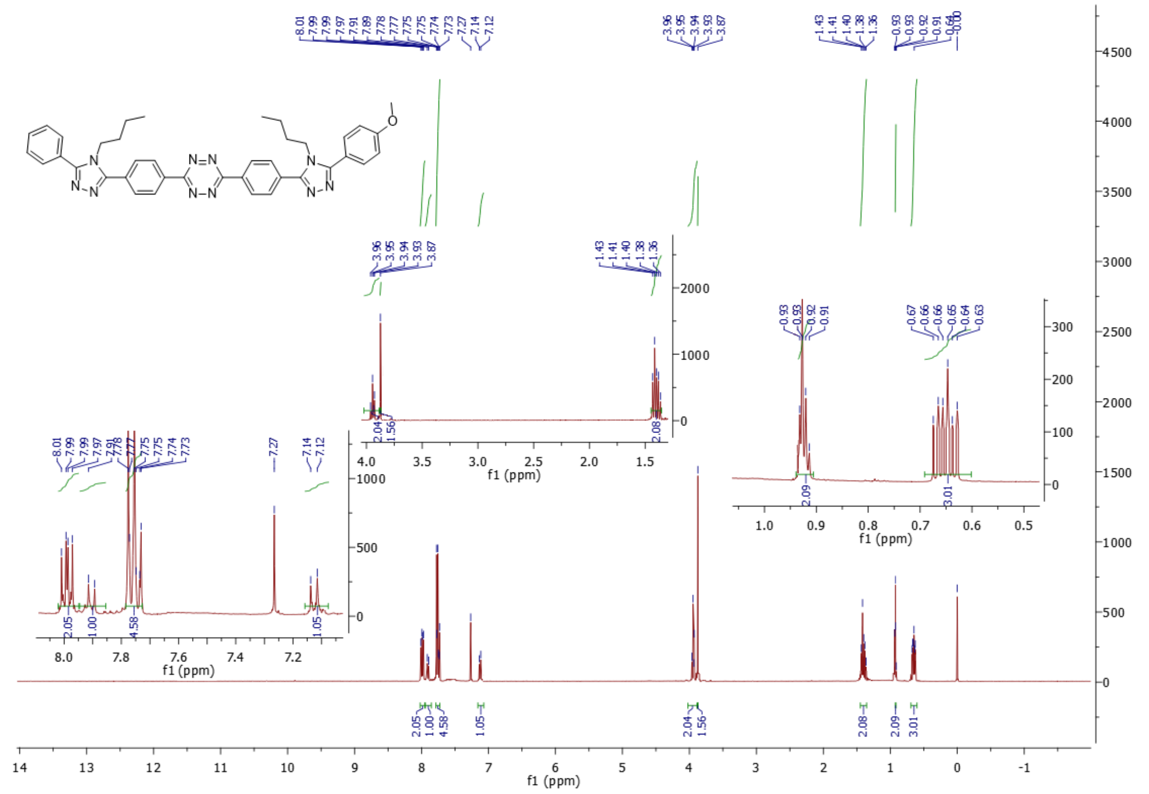

Figure S13. ^{1}H NMR spectra (400 MHz, $CDCl_3$) of 3-(4-(4-butyl-5-(4-methoxyphenyl)-4*H*-1,2,4-triazol-3-yl)phenyl)-6-(4-(4-butyl-5-phenyl-4*H*-1,2,4-triazol-3-yl)phenyl)-1,2,4,5-tetrazine (**10g**)

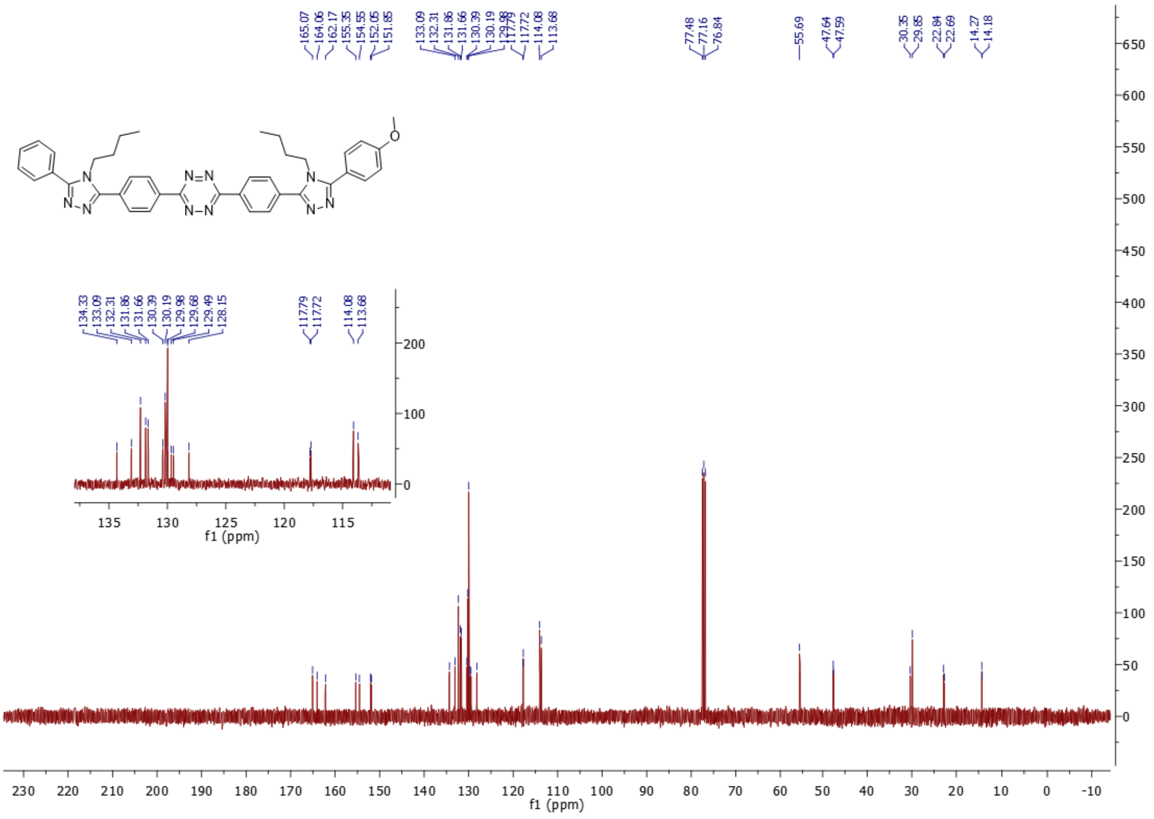

Figure S14. ^{13}C NMR spectra(100 MHz, $CDCl_3$) of 3-(4-(4-butyl-5-(4-methoxyphenyl)-4*H*-1,2,4-triazol-3-yl)phenyl)-6-(4-(4-butyl-5-phenyl-4*H*-1,2,4-triazol-3-yl)phenyl)-1,2,4,5-tetrazine (**10g**)

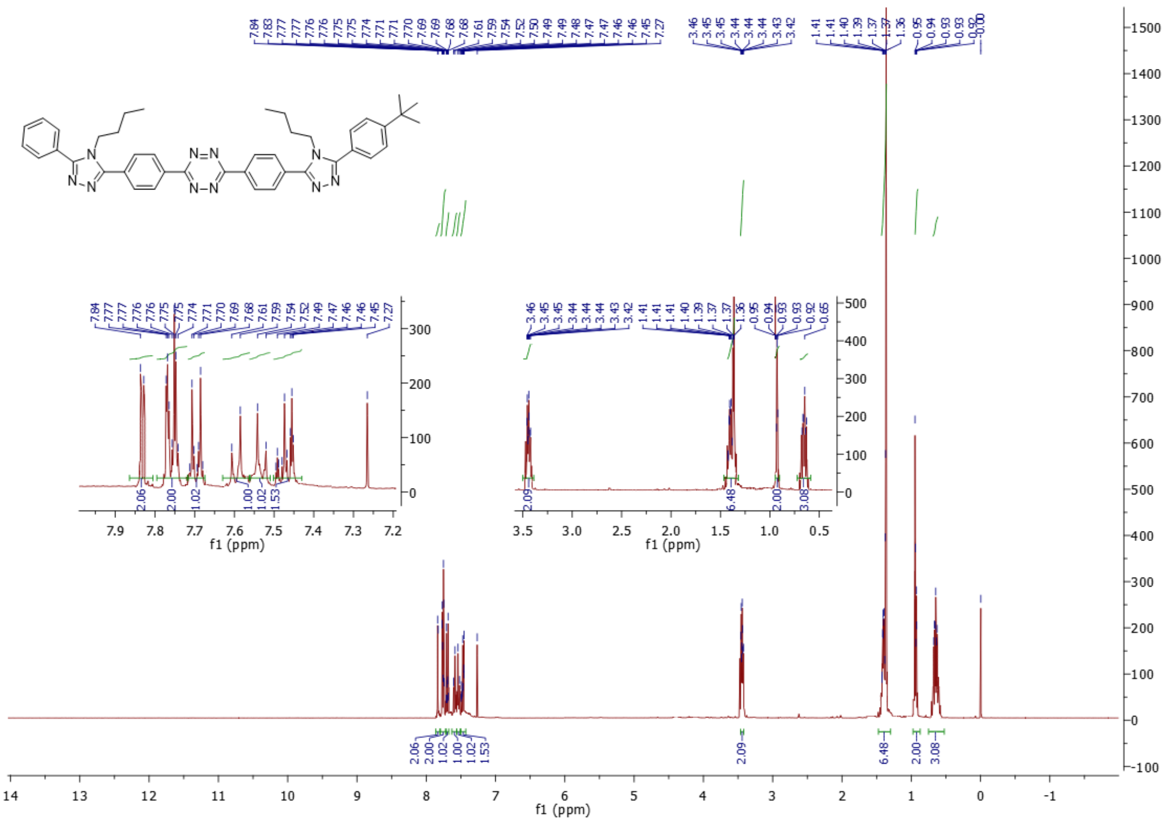

Figure S15. ^{1}H NMR spectra (400 MHz, $CDCl_3$) of 3-(4-(4-butyl-5-(4-(*tert*-butyl)phenyl)-4*H*-1,2,4-triazol-3-yl)phenyl)-6-(4-(4-butyl-5-phenyl-4*H*-1,2,4-triazol-3-yl)phenyl)-1,2,4,5-tetrazine (**10h**)

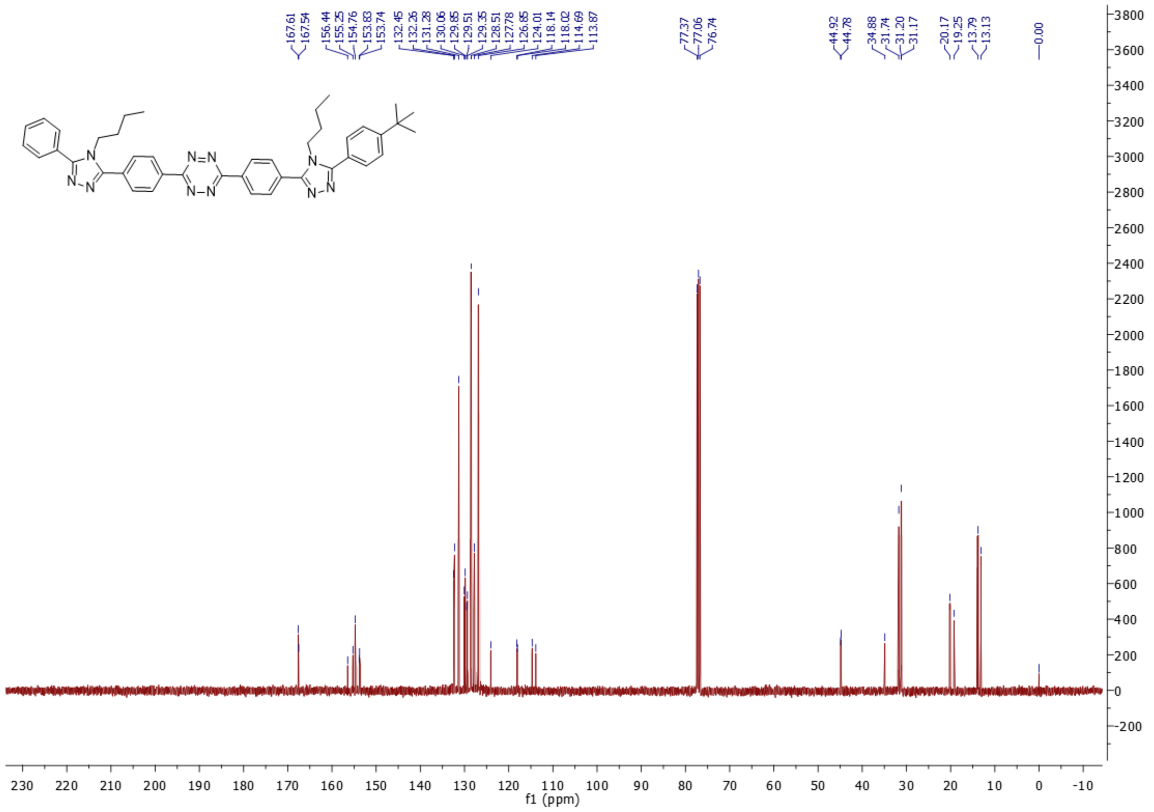

Figure S16. ^{13}C NMR spectra(100 MHz, $CDCl_3$)of 3-(4-(4-butyl-5-(4-(*tert*-butyl)phenyl)-4*H*-1,2,4-triazol-3-yl)phenyl)-6-(4-(4-butyl-5-phenyl-4*H*-1,2,4-triazol-3-yl)phenyl)-1,2,4,5-tetrazine (**10h**)

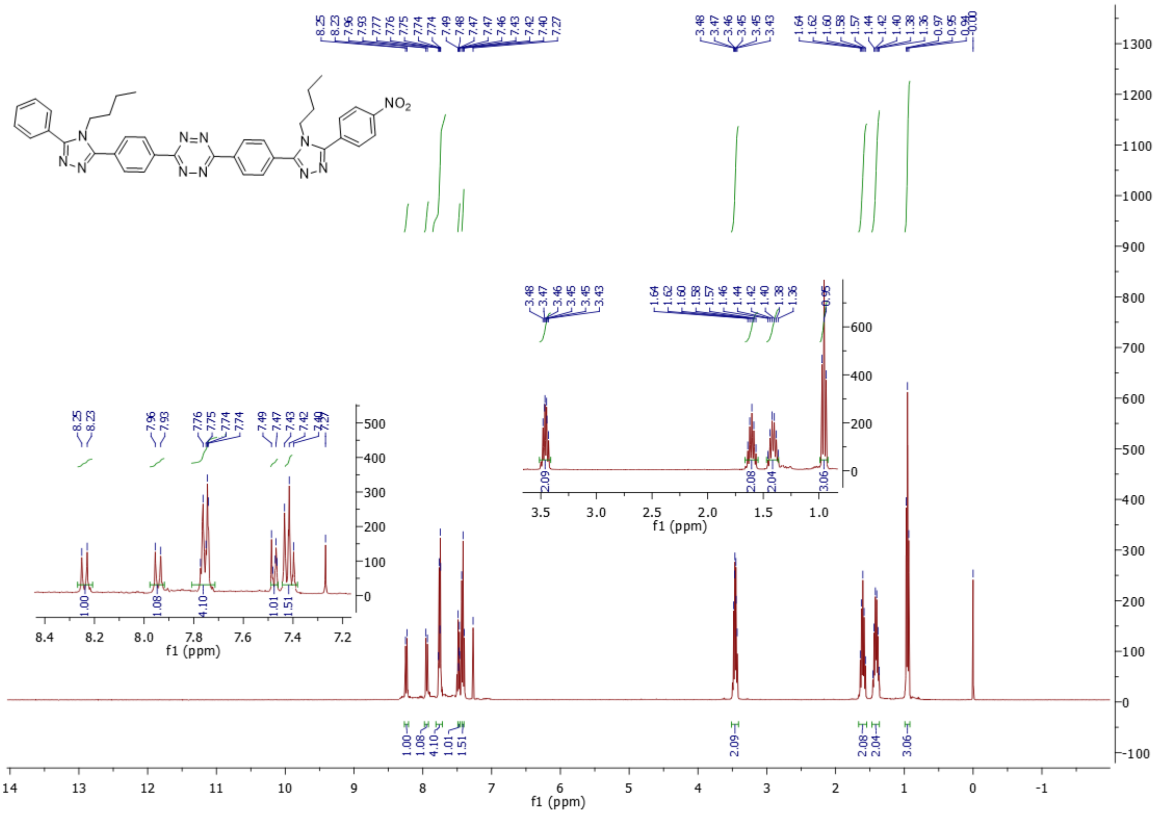

Figure S17. ^{1}H NMR spectra (400 MHz, $CDCl_3$) of 3-(4-(4-butyl-5-(4-nitrophenyl)-4*H*-1,2,4-triazol-3-yl)phenyl)-6-(4-(4-butyl-5-phenyl-4*H*-1,2,4-triazol-3-yl)phenyl)-1,2,4,5-tetrazine (**10i**)

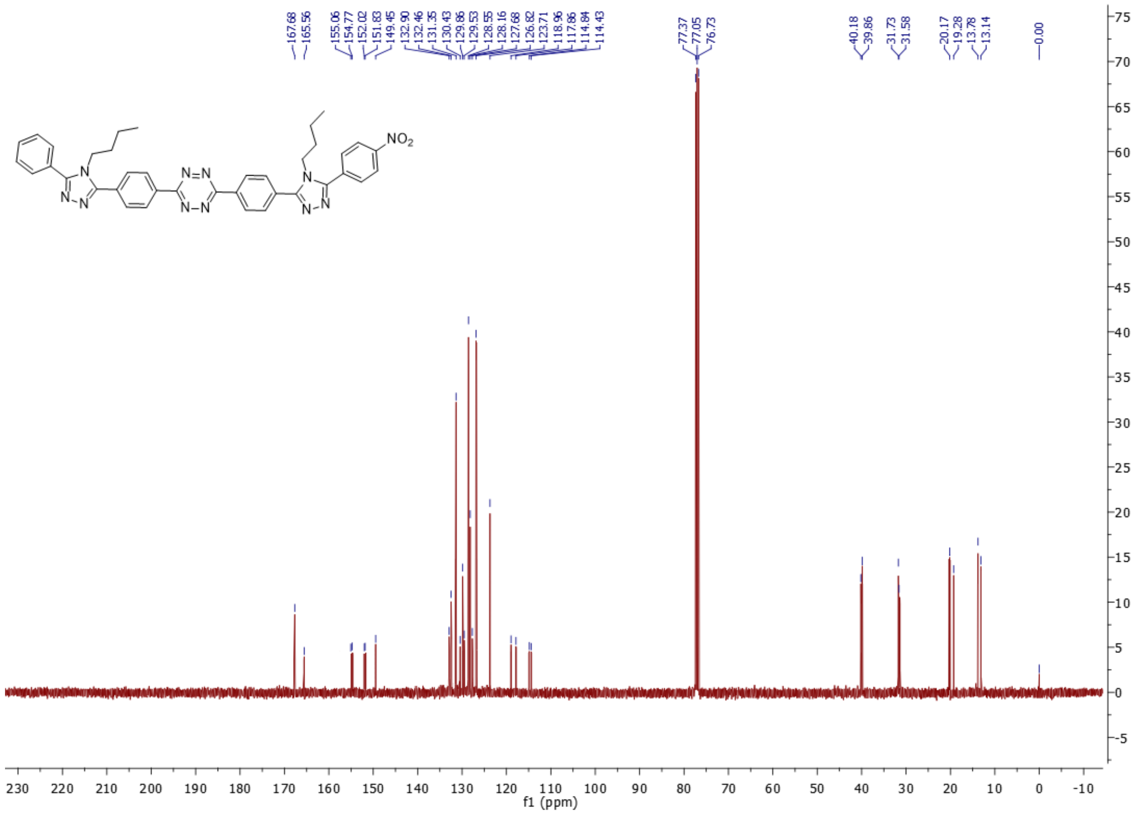

Figure S18. ^{13}C NMR spectra(100 MHz, $CDCl_3$)of 3-(4-(4-butyl-5-(4-nitrophenyl)-4*H*-1,2,4-triazol-3-yl)phenyl)-6-(4-(4-butyl-5-phenyl-4*H*-1,2,4-triazol-3-yl)phenyl)-1,2,4,5-tetrazine (**10i**)

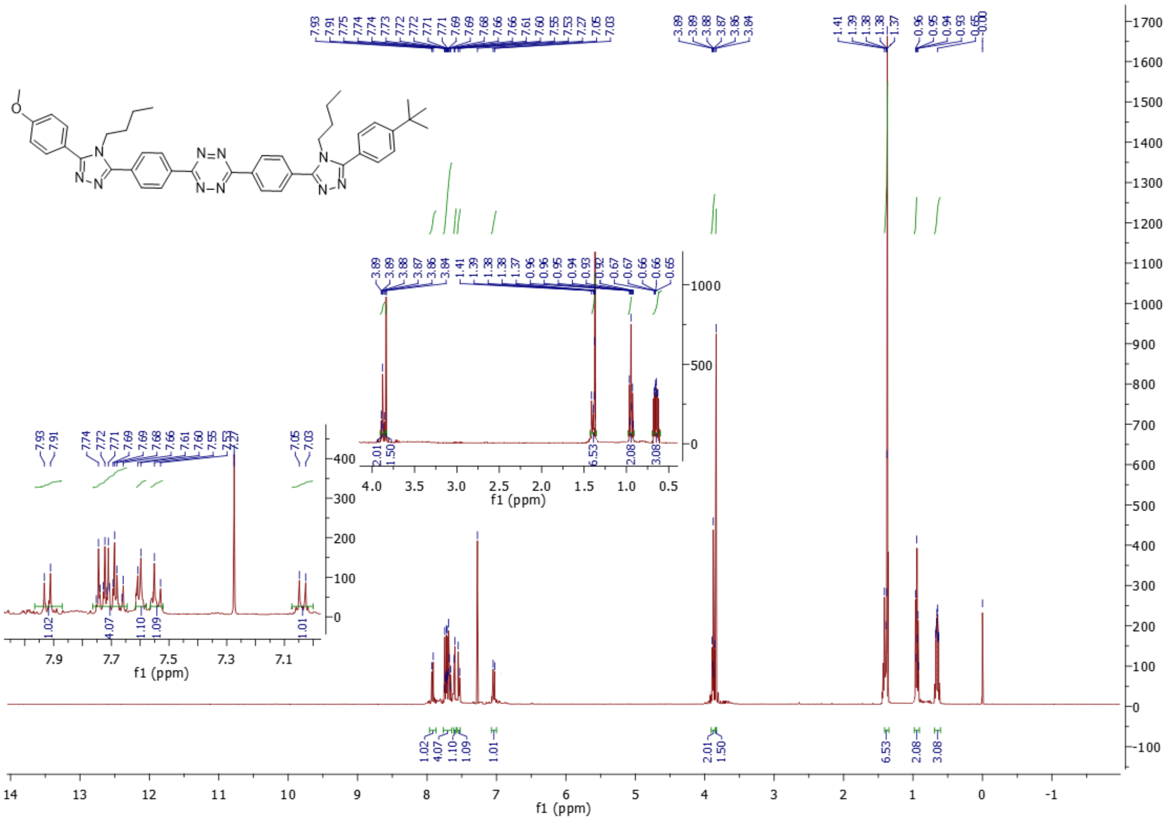

Figure S19. ^{1}H NMR spectra (400 MHz, $CDCl_3$) of 3-(4-(4-butyl-5-(4-(*tert*-butyl)phenyl)-4*H*-1,2,4-triazol-3-yl)phenyl)-6-(4-(4-butyl-5-(4-methoxyphenyl)-4*H*-1,2,4-triazol-3-yl)phenyl)-1,2,4,5-tetrazine (**10j**)

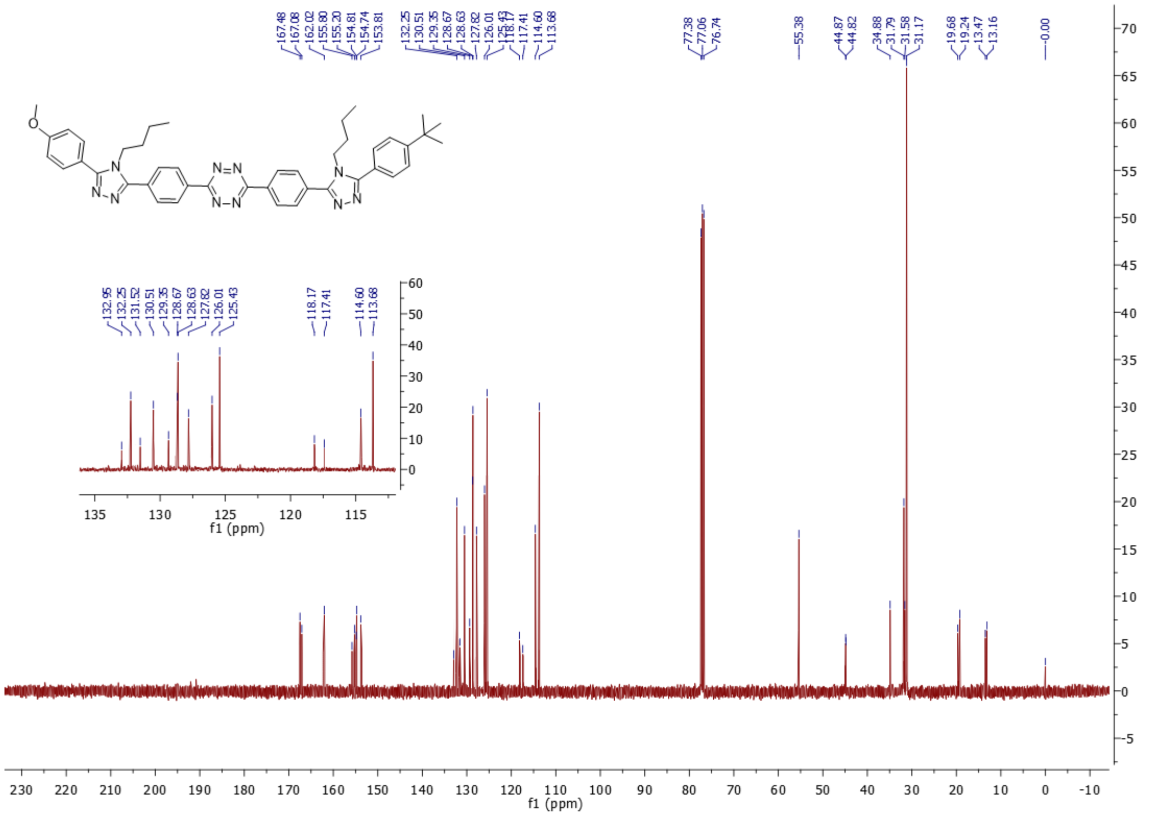

Figure S20. ^{13}C NMR spectra(100 MHz, $CDCl_3$)of 3-(4-(4-butyl-5-(4-(*tert*-butyl)phenyl)-4*H*-1,2,4-triazol-3-yl)phenyl)-6-(4-(4-butyl-5-(4-methoxyphenyl)-4*H*-1,2,4-triazol-3-yl)phenyl)-1,2,4,5-tetrazine (**10j**)

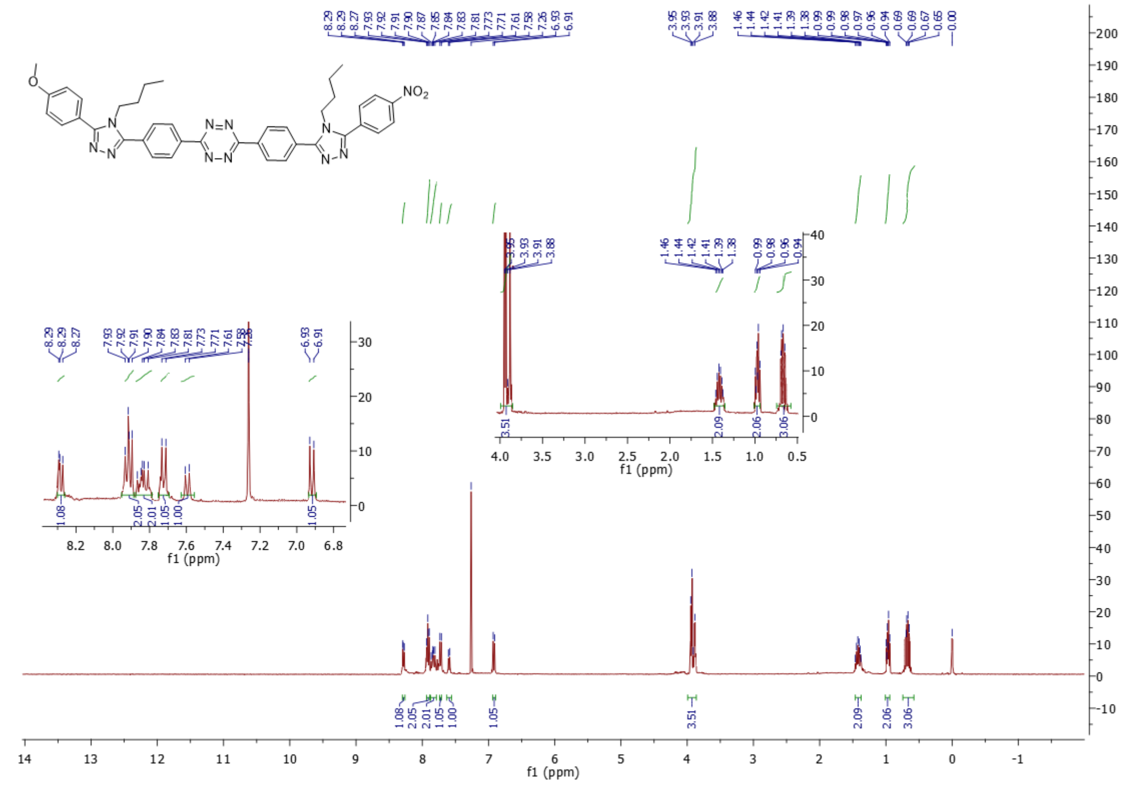

Figure S21. ^{1}H NMR spectra (400 MHz, $CDCl_3$) of 3-(4-(4-butyl-5-(4-methoxyphenyl)-4*H*-1,2,4-triazol-3-yl)phenyl)-6-(4-(4-butyl-5-(4-nitrophenyl)-4*H*-1,2,4-triazol-3-yl)phenyl)-1,2,4,5-tetrazine (**10k**)

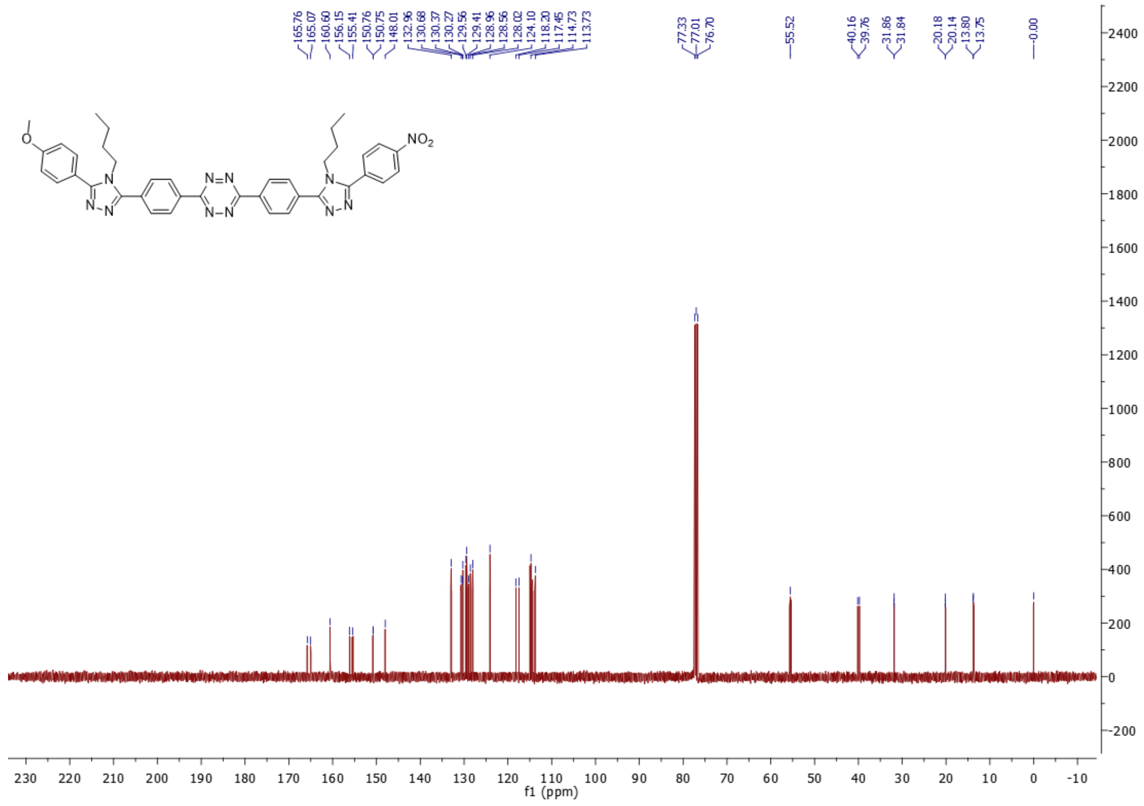

Figure S22. ^{13}C NMR spectra(100 MHz, $CDCl_3$)of 3-(4-(4-butyl-5-(4-methoxyphenyl)-4*H*-1,2,4-triazol-3-yl)phenyl)-6-(4-(4-butyl-5-(4-nitrophenyl)-4*H*-1,2,4-triazol-3-yl)phenyl)-1,2,4,5-tetrazine (**10k**)

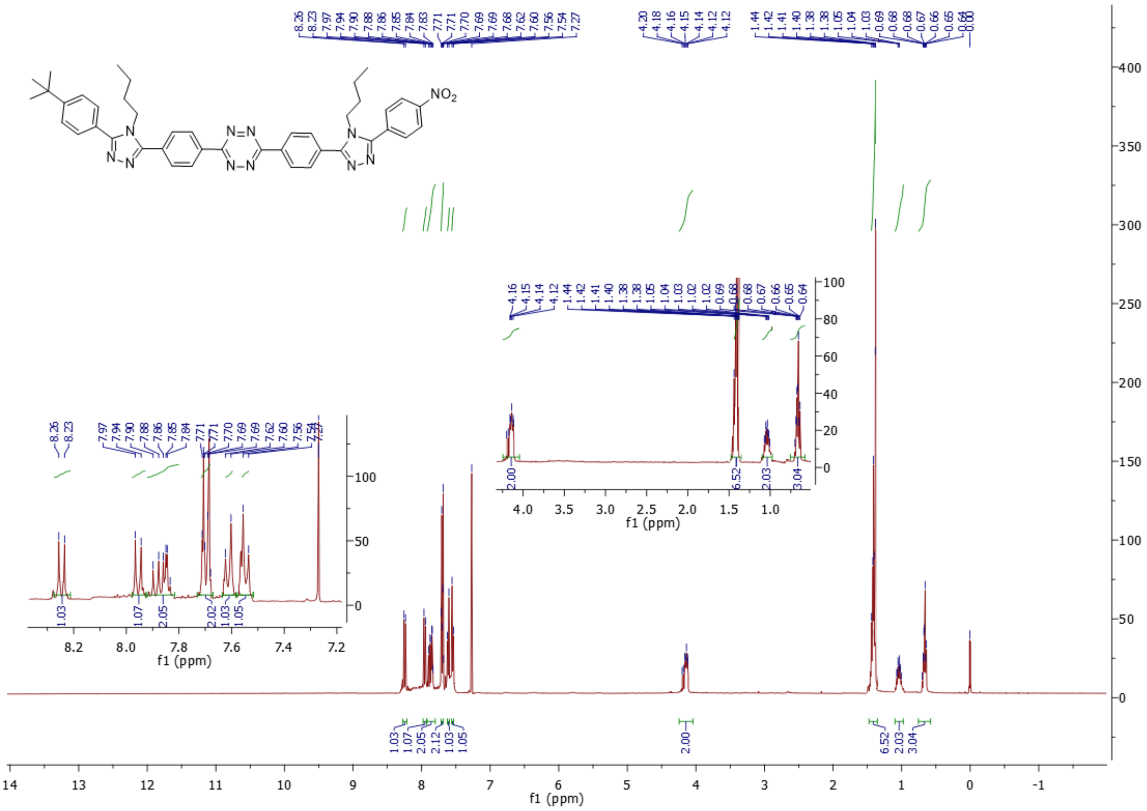

Figure S23. ^{1}H NMR spectra (400 MHz, $CDCl_3$) of 3-(4-(4-butyl-5-(4-(*tert*-butyl)phenyl)-4*H*-1,2,4-triazol-3-yl)phenyl)-6-(4-(4-butyl-5-(4-nitrophenyl)-4*H*-1,2,4-triazol-3-yl)phenyl)-1,2,4,5-tetrazine (**10l**)

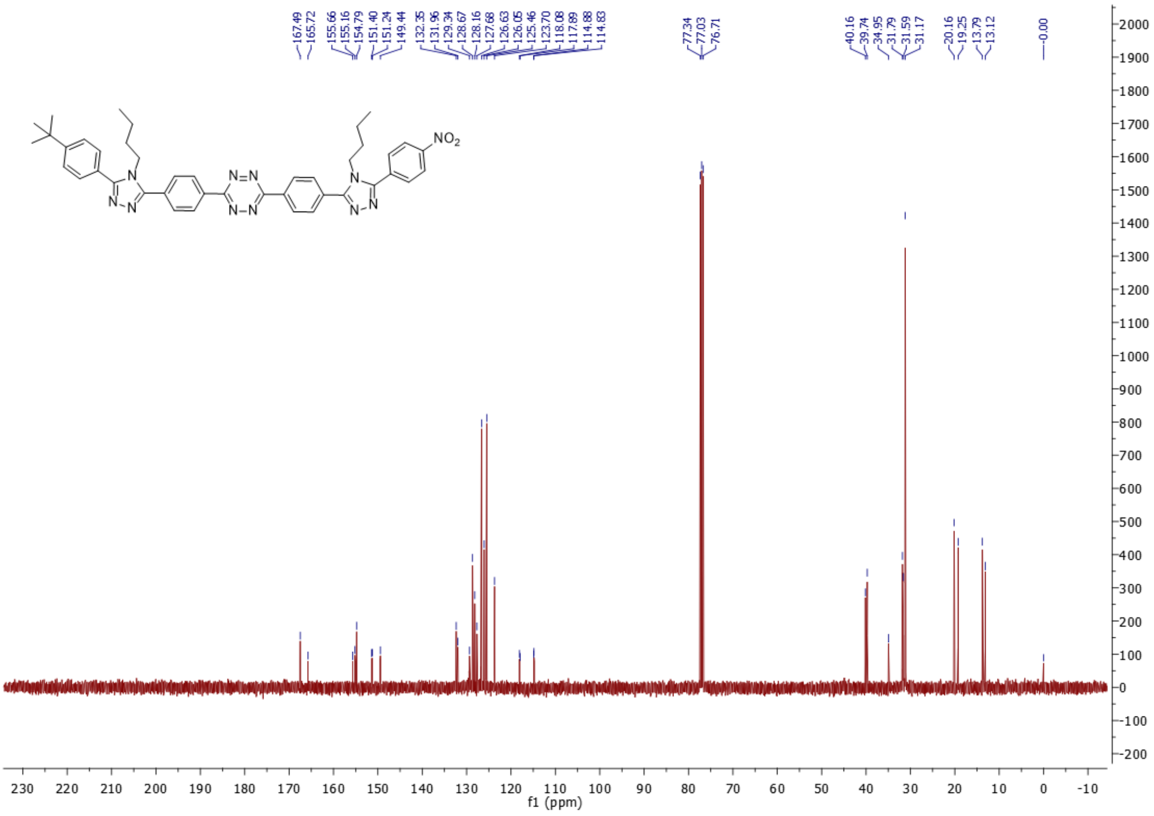

Figure S24. ^{13}C NMR spectra(100 MHz, $CDCl_3$)f 3-(4-(4-butyl-5-(4-(*tert*-butyl)phenyl)-4*H*-1,2,4-triazol-3-yl)phenyl)-6-(4-(4-butyl-5-(4-nitrophenyl)-4*H*-1,2,4-triazol-3-yl)phenyl)-1,2,4,5-tetrazine (**10l**)

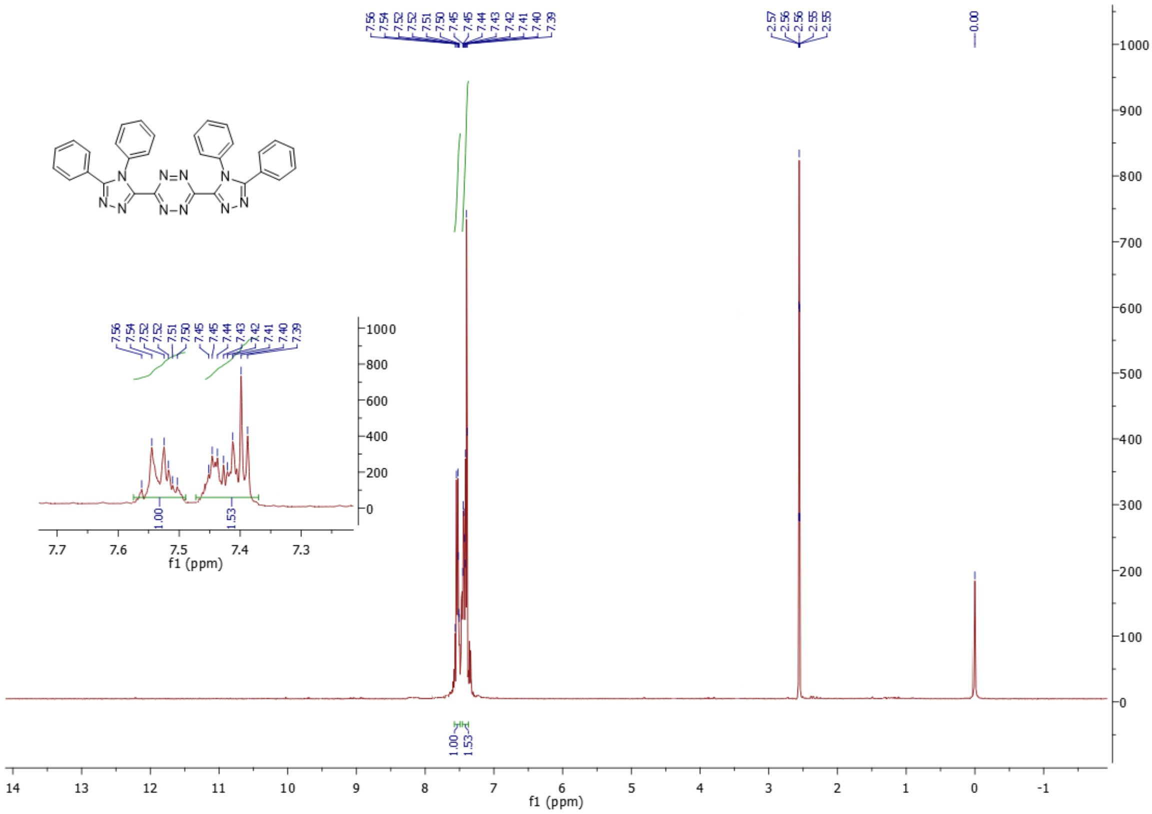

Figure S25. ^{1}H NMR spectra (400 MHz, DMSO-d_6) of 3,6-bis(4,5-diphenyl-4*H*-1,2,4-triazol-3-yl)-1,2,4,5-tetrazine (**15a**)

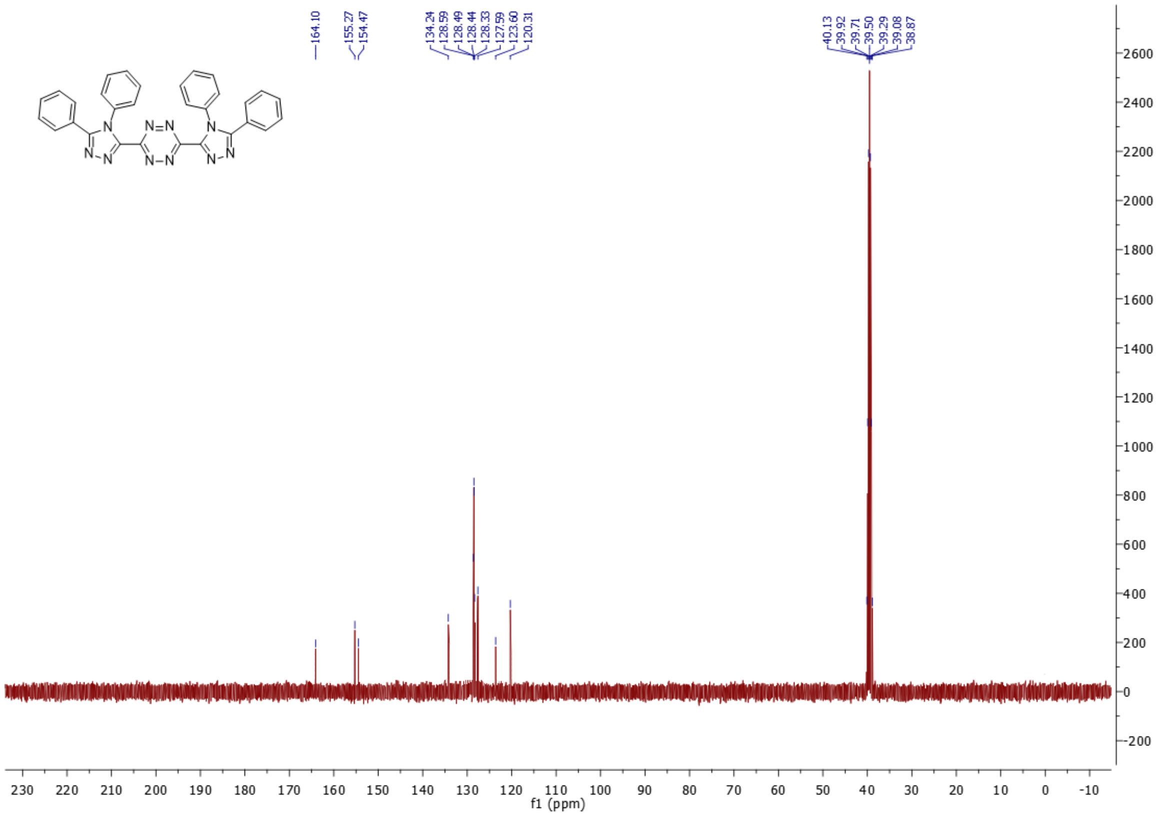

Figure S26. ^{13}C NMR spectra(100 MHz, DMSO-d_6)of 3,6-bis(4,5-diphenyl-4*H*-1,2,4-triazol-3-yl)-1,2,4,5-tetrazine (**15a**)

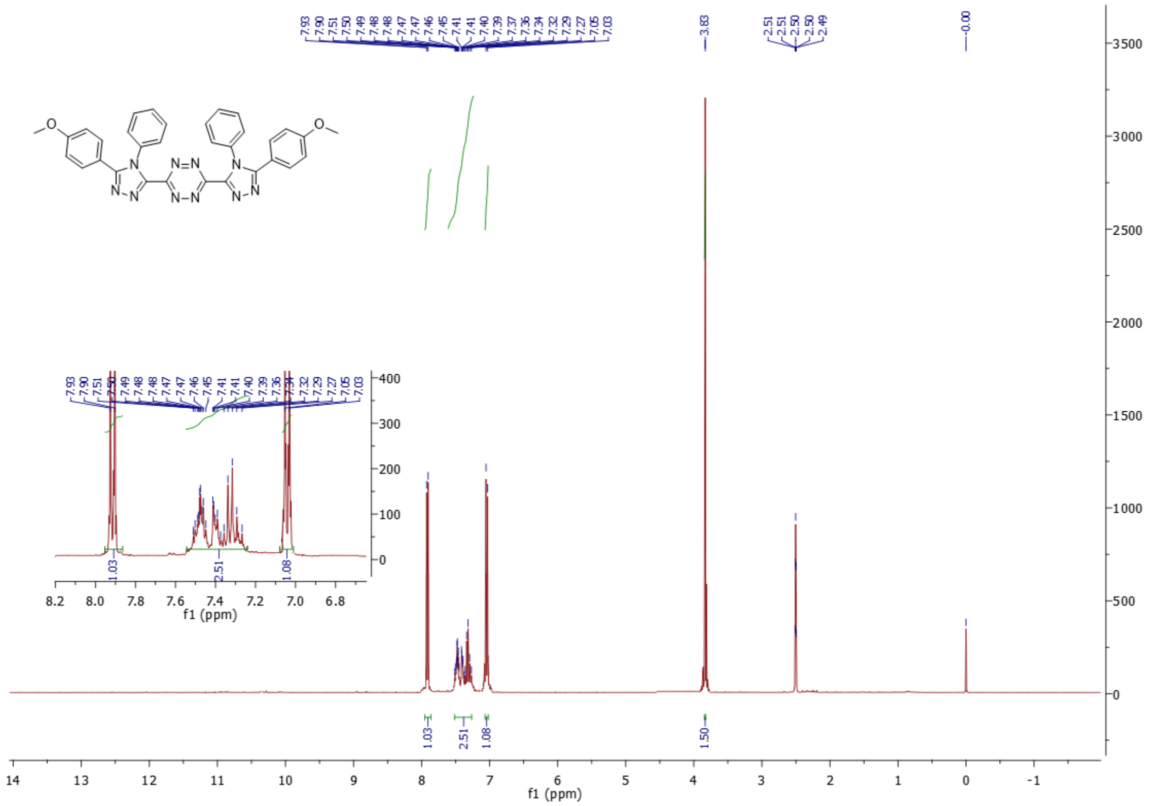

Figure S27. ^{1}H NMR spectra (400 MHz, DMSO-d_6) of 3,6-bis(5-(4-methoxyphenyl)-4-phenyl-4*H*-1,2,4-triazol-3-yl)-1,2,4,5-tetrazine (**15b**)

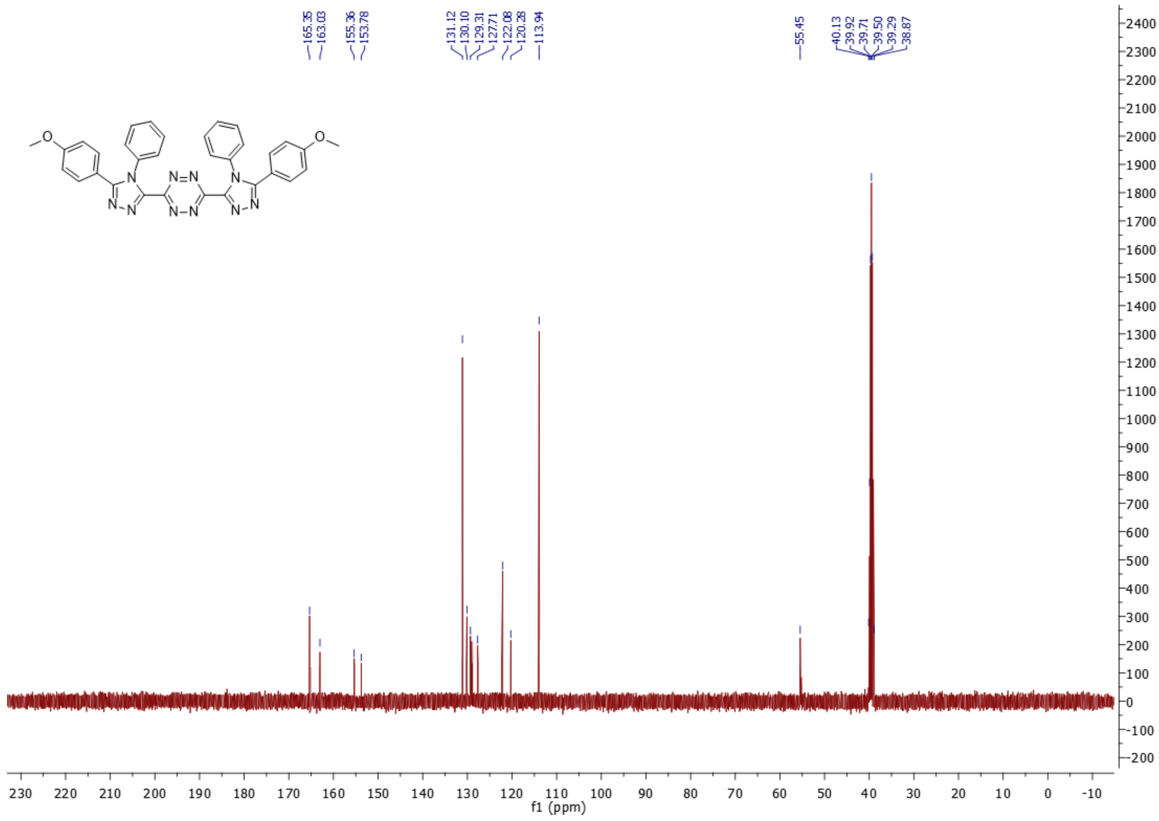

Figure S28. ^{13}C NMR spectra(100 MHz, DMSO-d_6)of 3,6-bis(5-(4-methoxyphenyl)-4-phenyl-4*H*-1,2,4-triazol-3-yl)-1,2,4,5-tetrazine (**15b**)

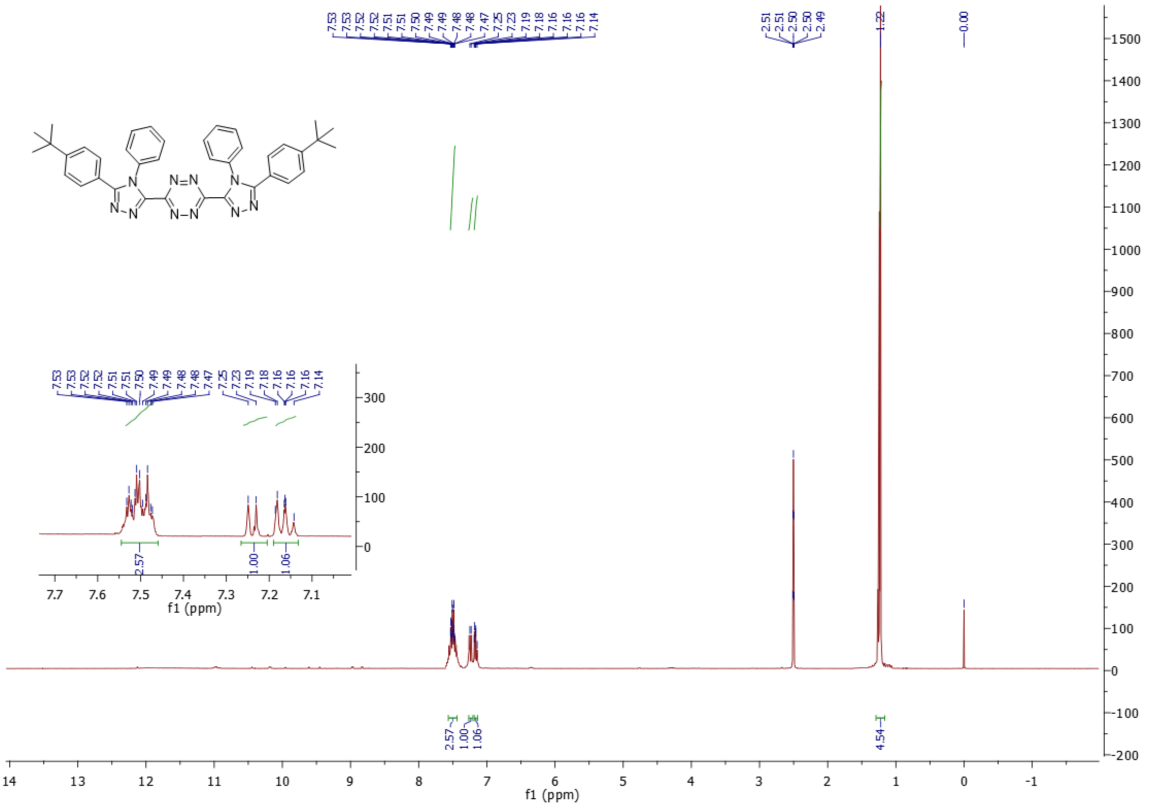

Figure S29. ^{1}H NMR spectra (400 MHz, DMSO-d_6) of 3,6-bis(5-(4-(*tert*-butyl)phenyl)-4-phenyl-4*H*-1,2,4-triazol-3-yl)-1,2,4,5-tetrazine (**15c**)

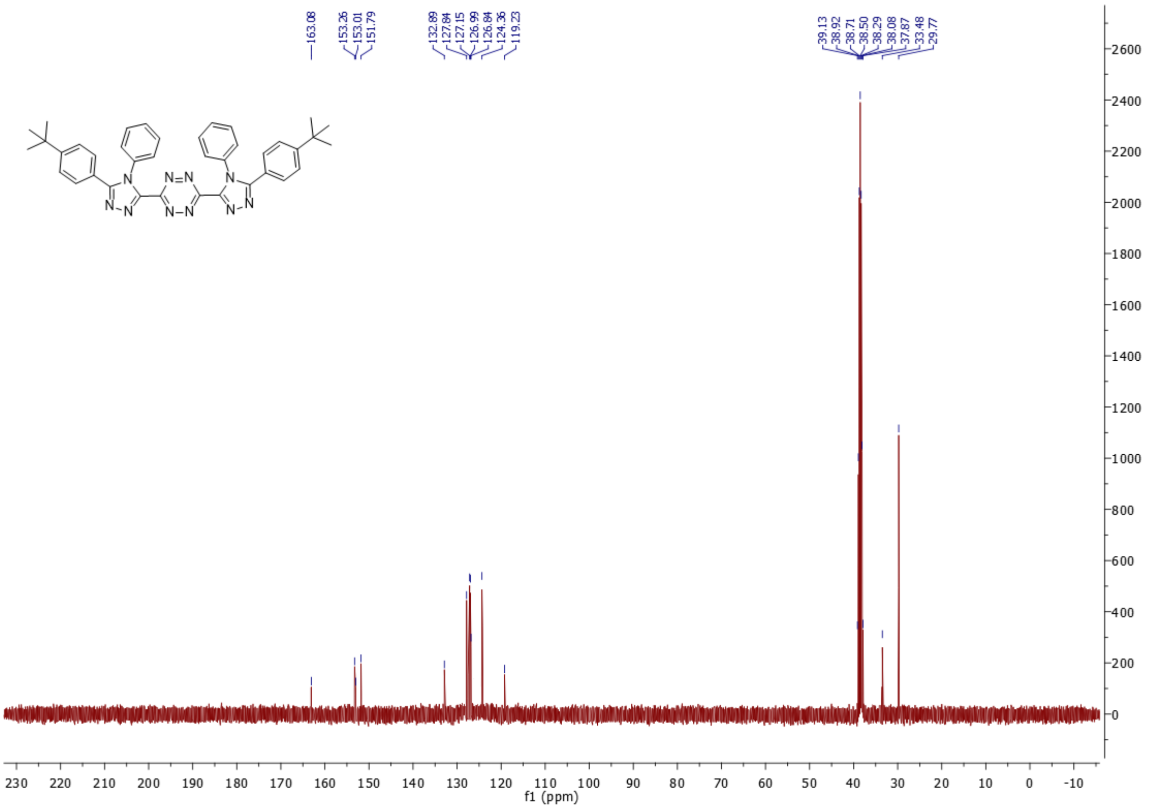

Figure S30. ^{13}C NMR spectra(100 MHz, DMSO-d_6)of 3,6-bis(5-(4-(*tert*-butyl)phenyl)-4-phenyl-4*H*-1,2,4-triazol-3-yl)-1,2,4,5-tetrazine (**15c**)

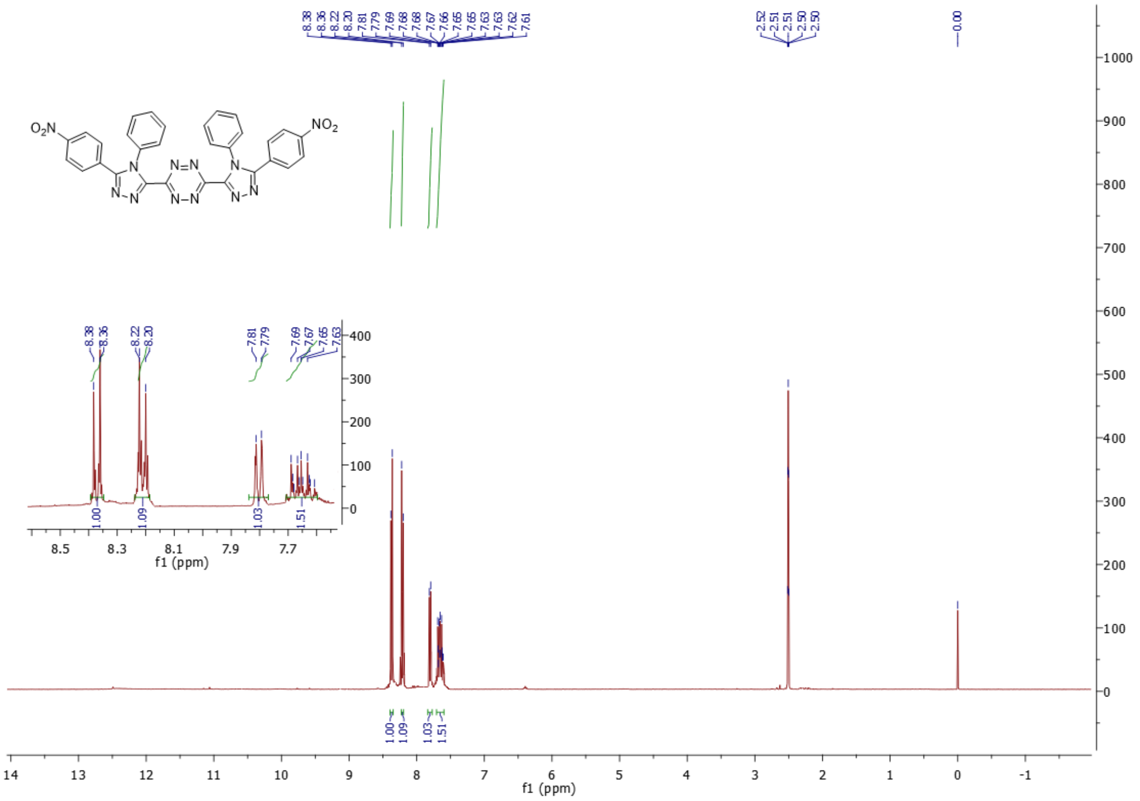

Figure S31. ^{1}H NMR spectra (400 MHz, DMSO-d$_6$) of 3,6-bis(5-(4-nitrophenyl)-4-phenyl-4*H*-1,2,4-triazol-3-yl)-1,2,4,5-tetrazine (**15d**)

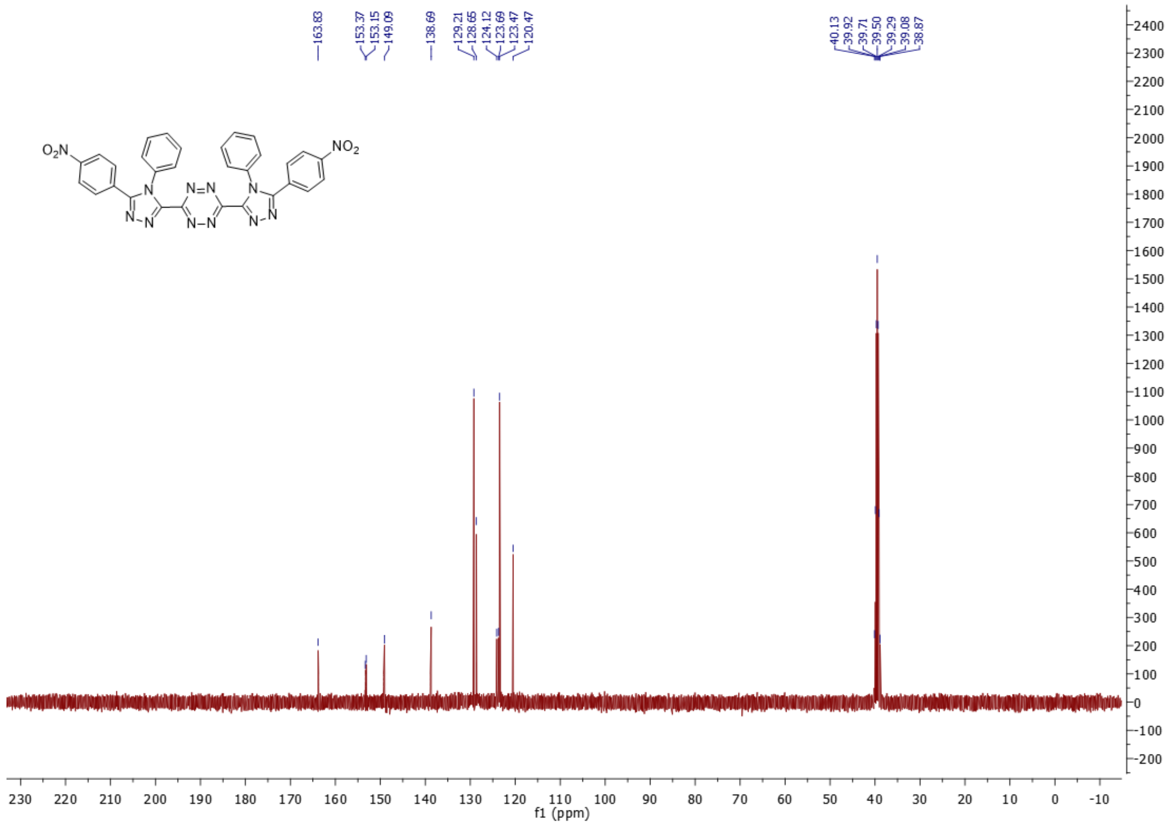

Figure S32. ^{13}C NMR spectra(100 MHz, DMSO-d$_6$)of 3,6-bis(5-(4-nitrophenyl)-4-phenyl-4*H*-1,2,4-triazol-3-yl)-1,2,4,5-tetrazine (**15d**)

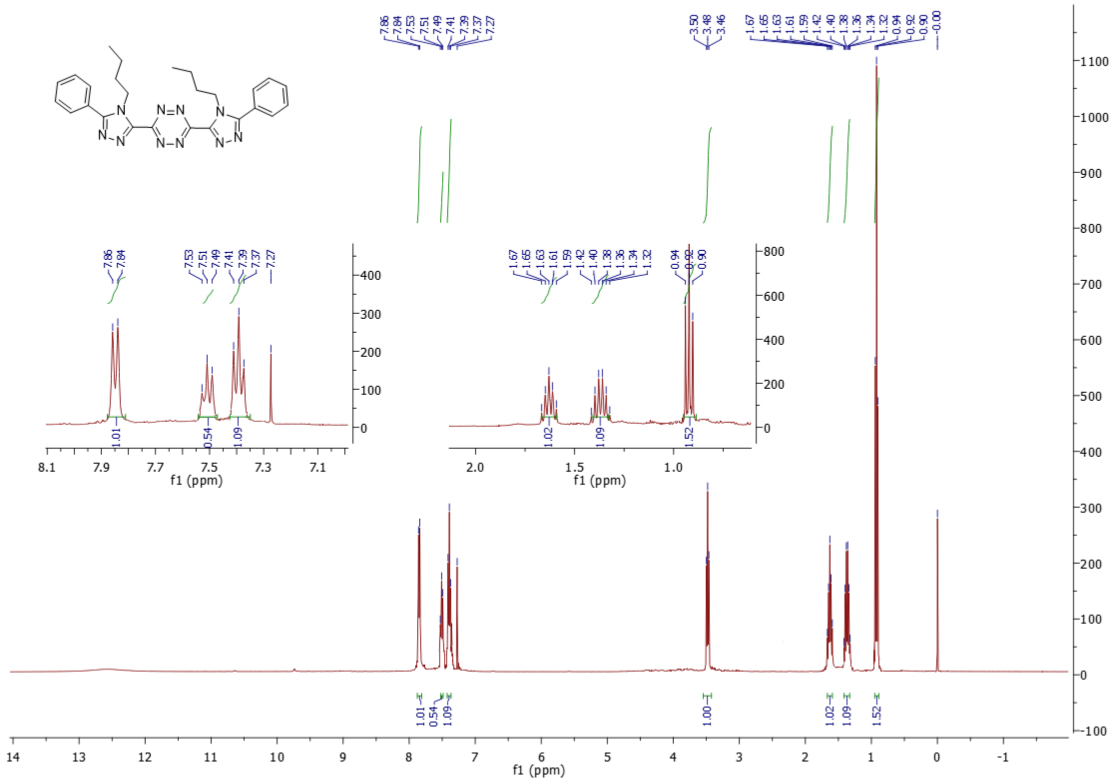

Figure S33. ^{1}H NMR spectra (400 MHz, $CDCl_3$)of 3,6-bis(4-butyl-5-phenyl-4*H*-1,2,4-triazol-3-yl)-1,2,4,5-tetrazine (**15e**)

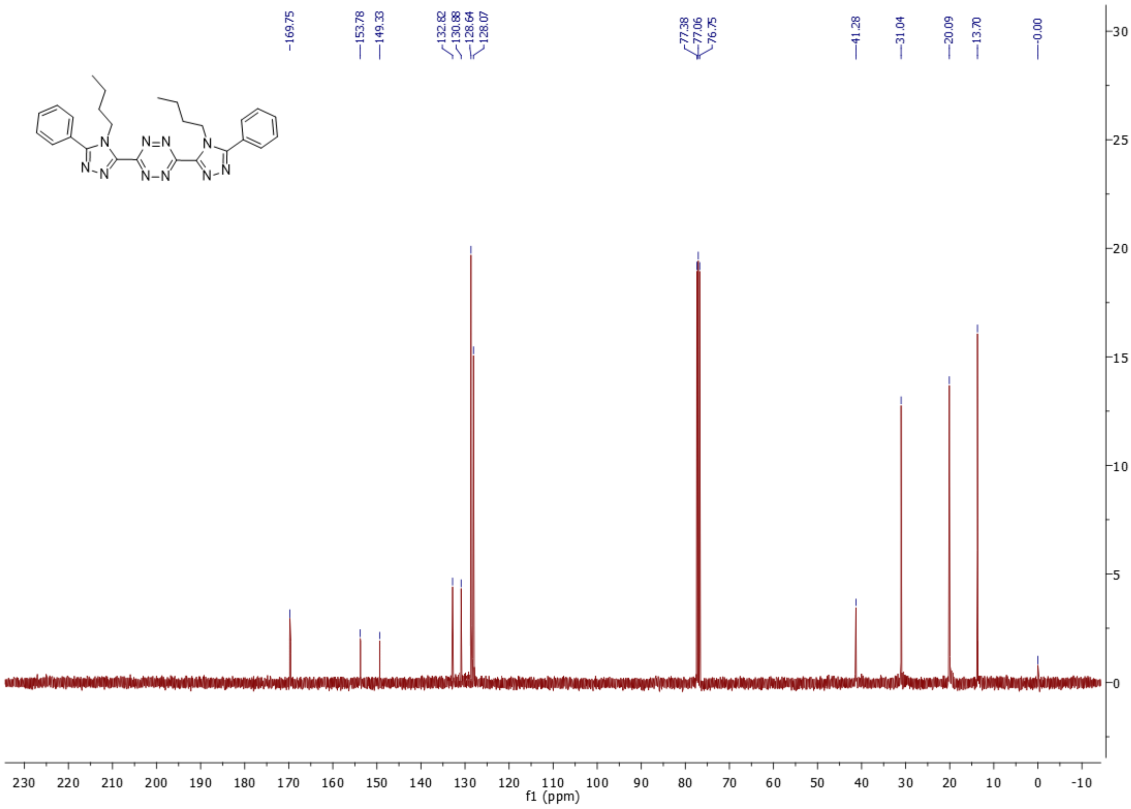

Figure S34. ^{13}C NMR spectra(100 MHz, $CDCl_3$)of 3,6-bis(4-butyl-5-phenyl-4*H*-1,2,4-triazol-3-yl)-1,2,4,5-tetrazine (**15e**)

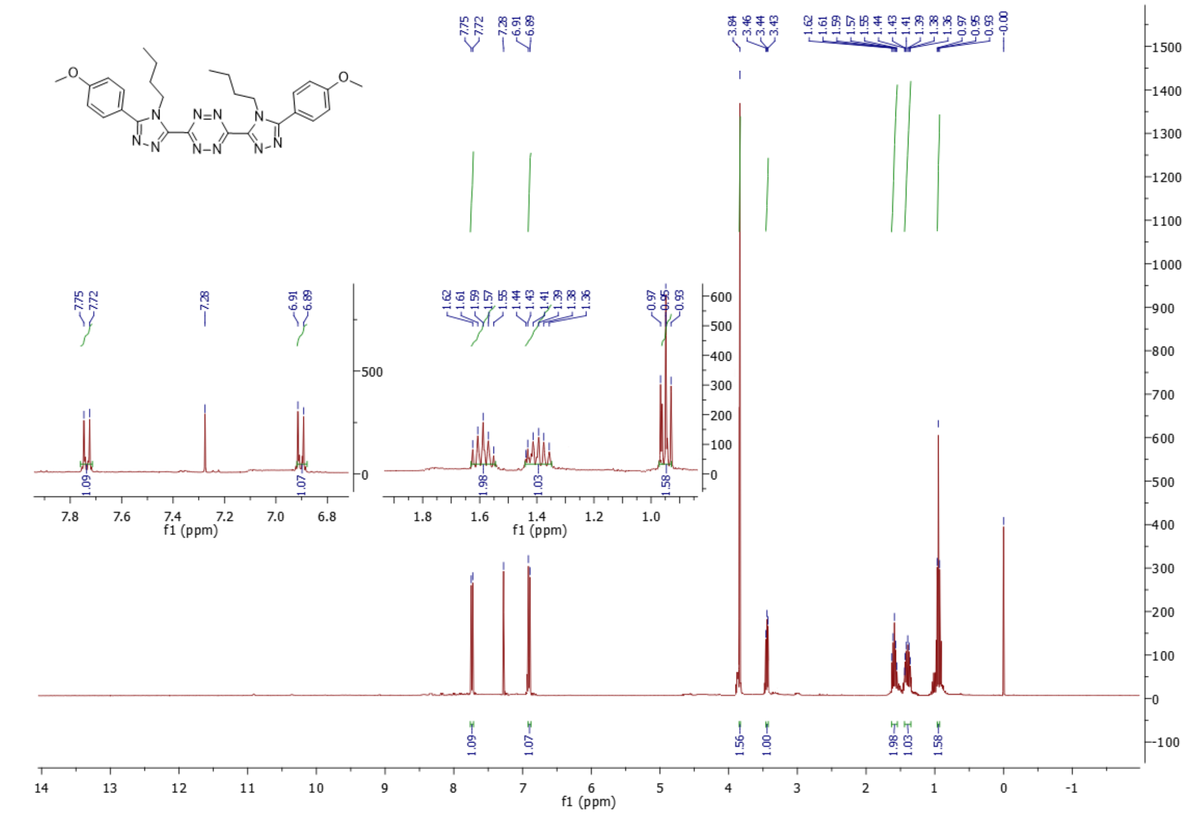

Figure S35. ^{1}H NMR spectra (400 MHz, $CDCl_3$) of 3,6-bis(4-butyl-5-(4-methoxyphenyl)-4*H*-1,2,4-triazol-3-yl)-1,2,4,5-tetrazine (**15f**)

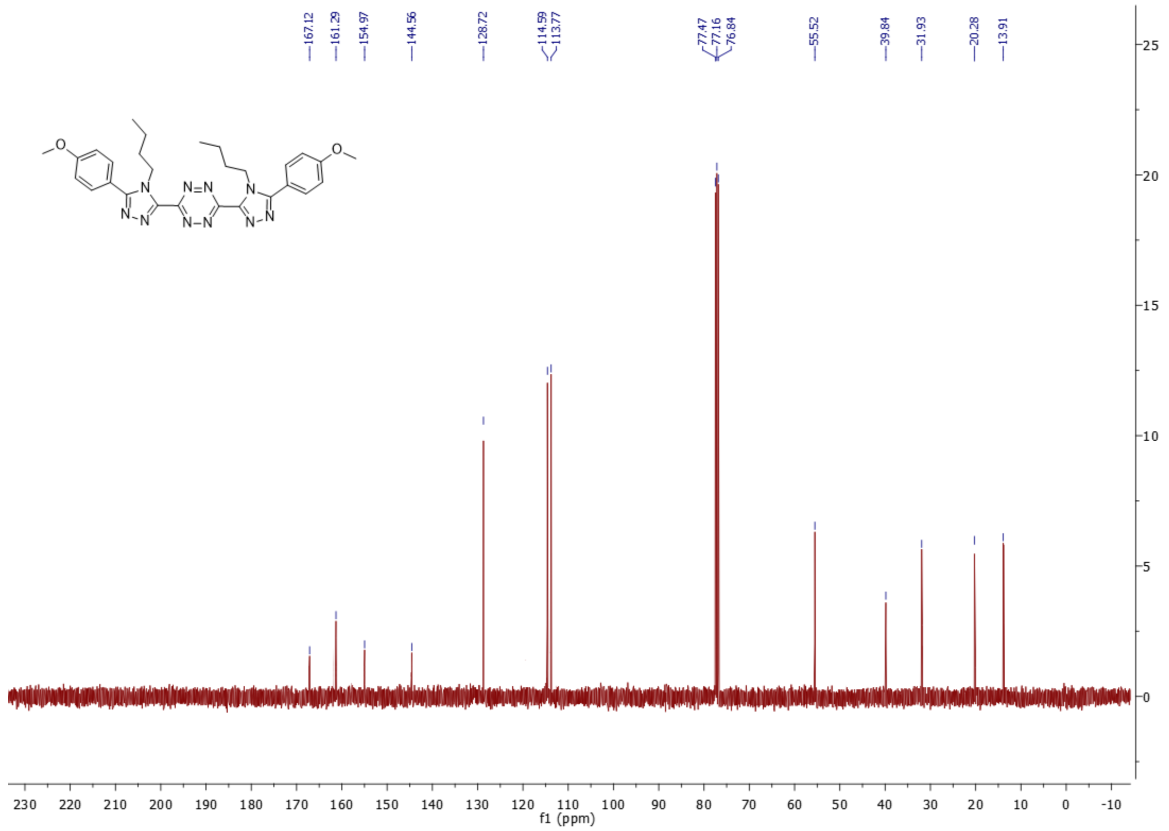

Figure S36. ^{13}C NMR spectra(100 MHz, $CDCl_3$)of 3,6-bis(4-butyl-5-(4-methoxyphenyl)-4*H*-1,2,4-triazol-3-yl)-1,2,4,5-tetrazine (**15f**)

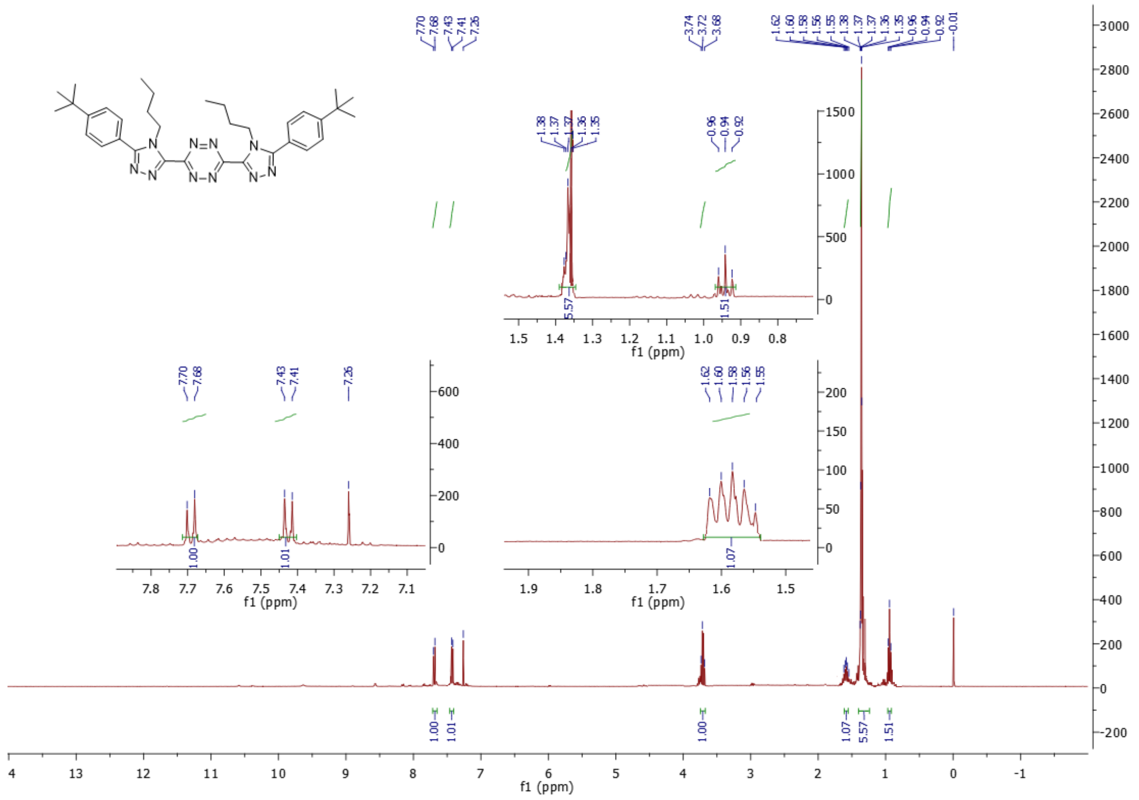

Figure S37. ^{1}H NMR spectra (400 MHz, $CDCl_3$) of 3,6-bis(4-butyl-5-(4-(*tert*-butyl)phenyl)-4*H*-1,2,4-triazol-3-yl)-1,2,4,5-tetrazine (**15g**)

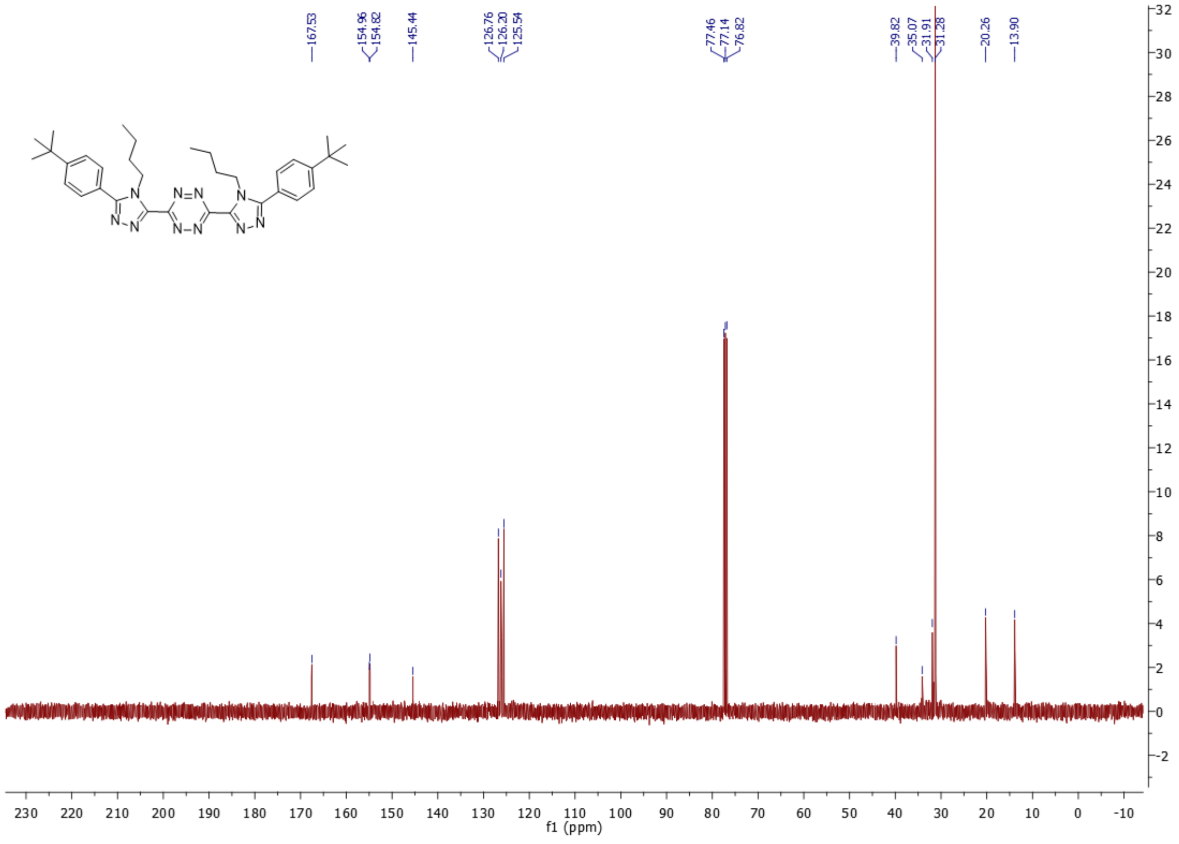

Figure S38. ^{13}C NMR spectra(100 MHz, $CDCl_3$)of 3,6-bis(4-butyl-5-(4-(*tert*-butyl)phenyl)-4*H*-1,2,4-triazol-3-yl)-1,2,4,5-tetrazine (**15g**)

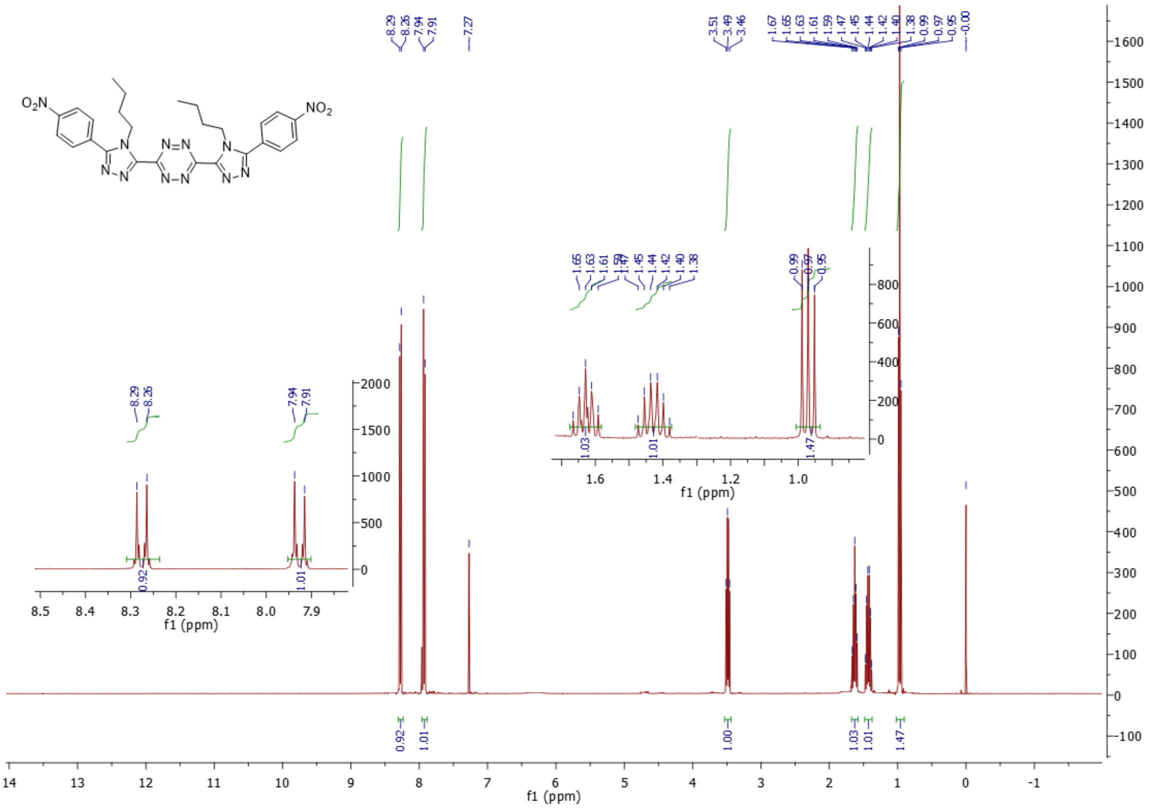

Figure S39. ^{1}H NMR spectra (400 MHz, $CDCl_3$) of 3,6-bis(4-butyl-5-(4-nitrophenyl)-4*H*-1,2,4-triazol-3-yl)-1,2,4,5-tetrazine (**15h**)

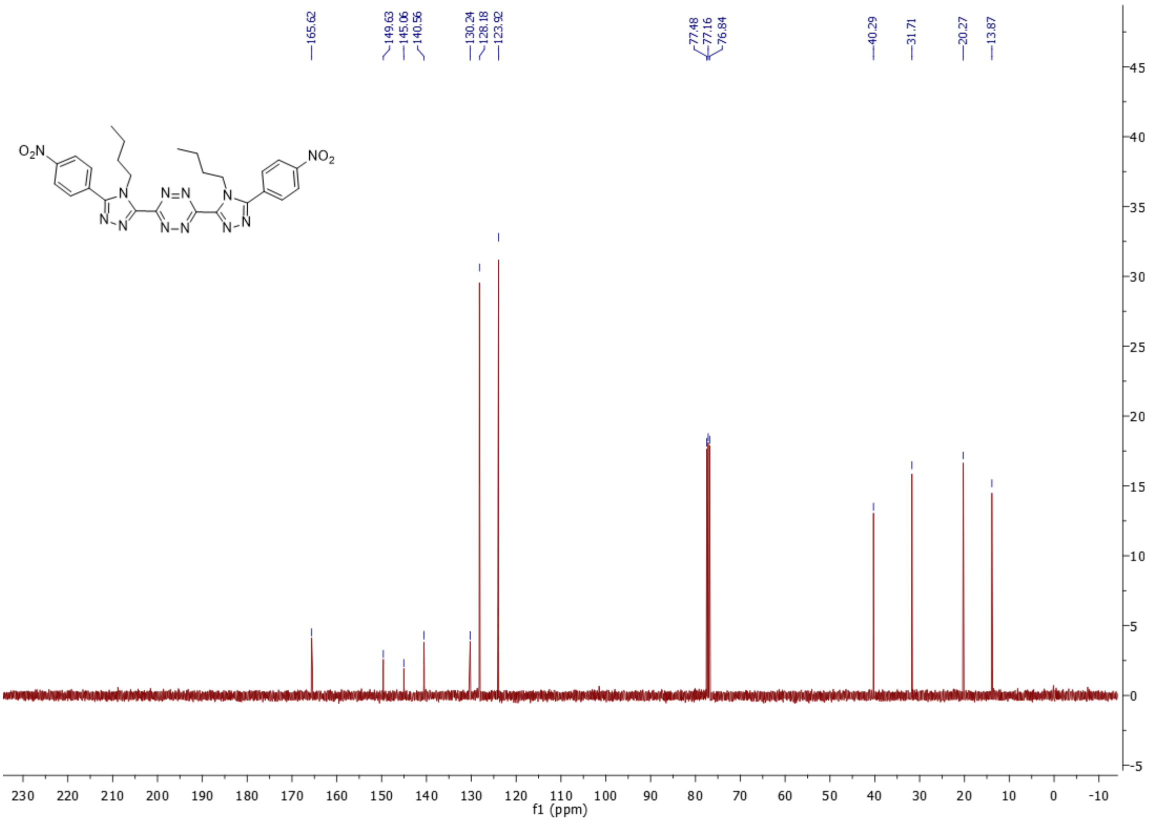

Figure S40. ^{13}C NMR spectra(100 MHz, $CDCl_3$) of 3,6-bis(4-butyl-5-(4-nitrophenyl)-4*H*-1,2,4-triazol-3-yl)-1,2,4,5-tetrazine (**15h**)

2. UV-Vis absorption spectra

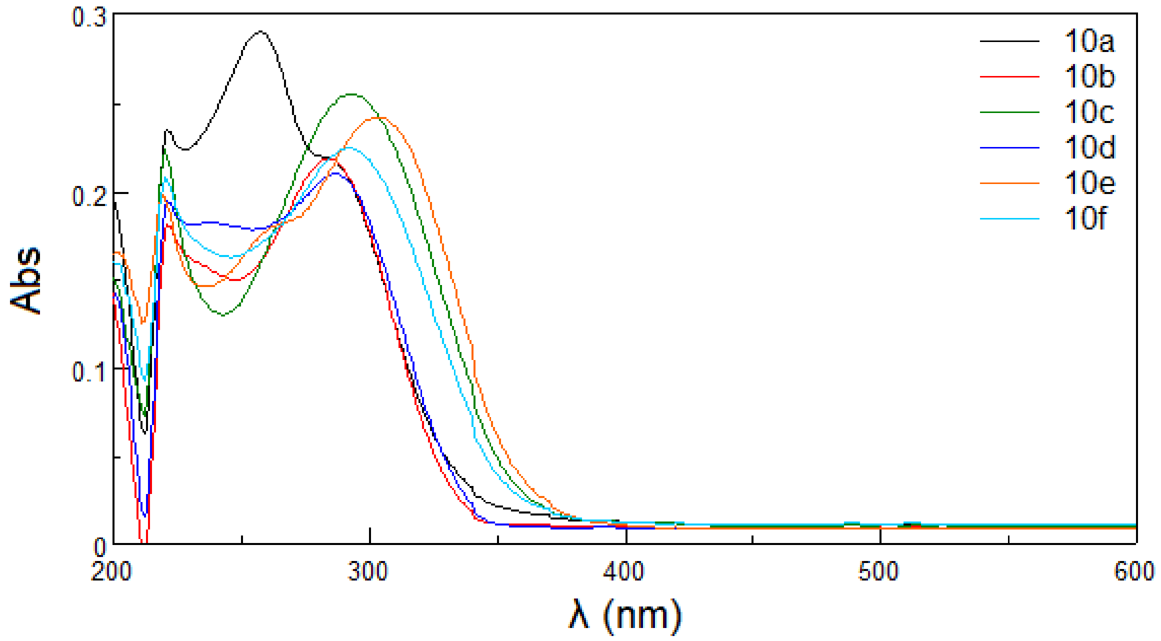

Figure S41. UV-Vis absorption spectra of **10a-f**

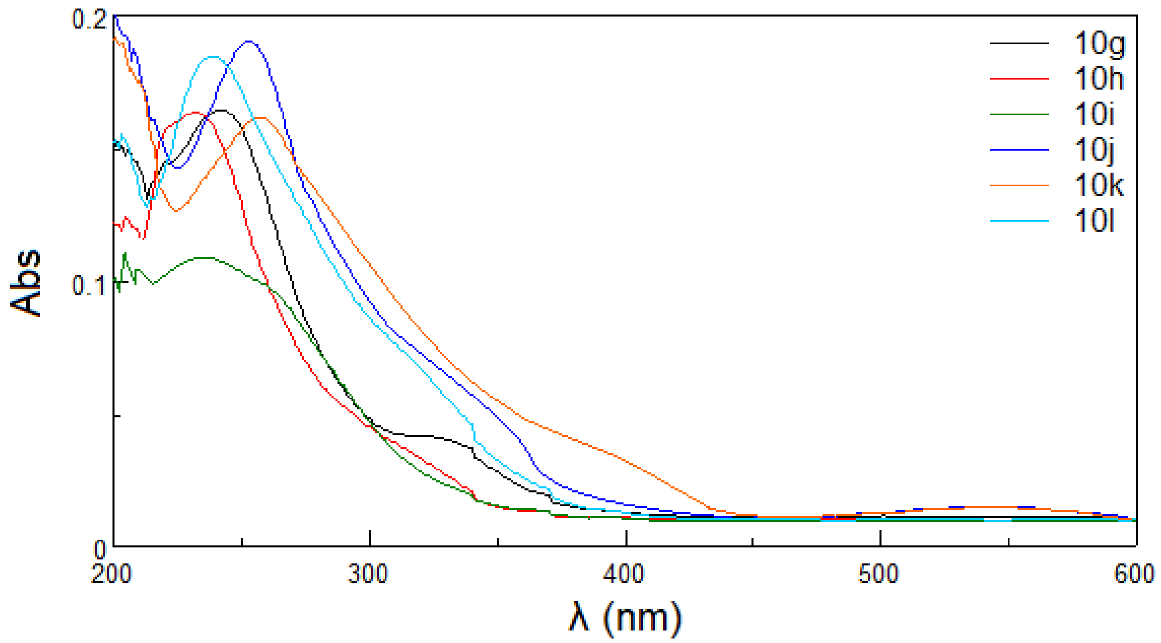

Figure S42. UV-Vis absorption spectra of **10g-l**

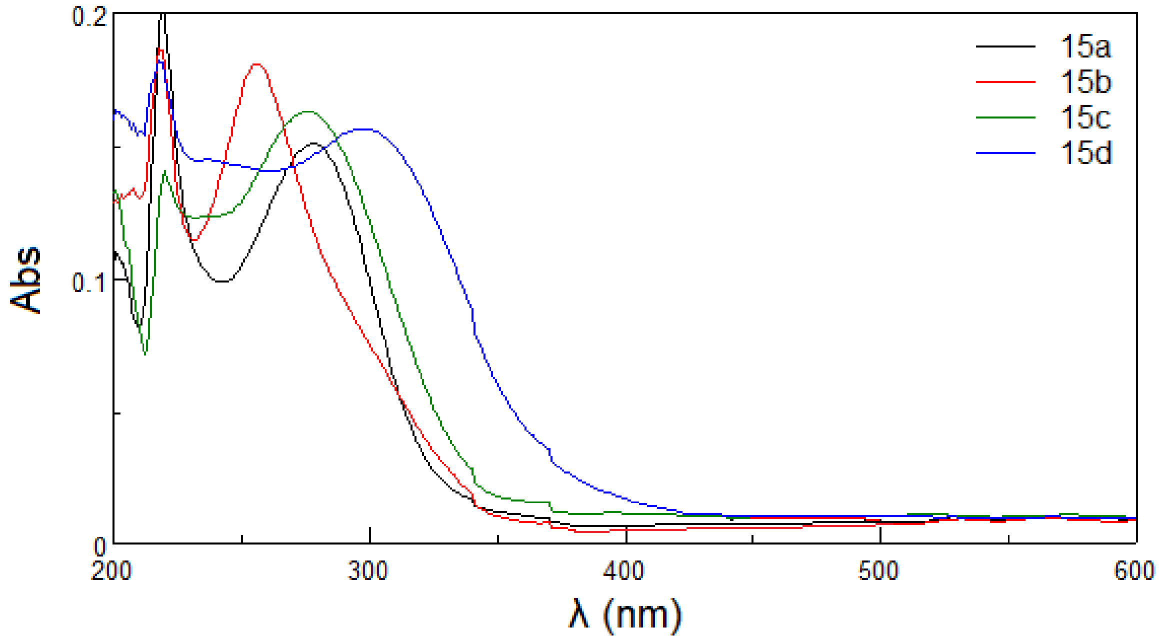

Figure S43. UV-Vis absorption spectra of **15a-d**

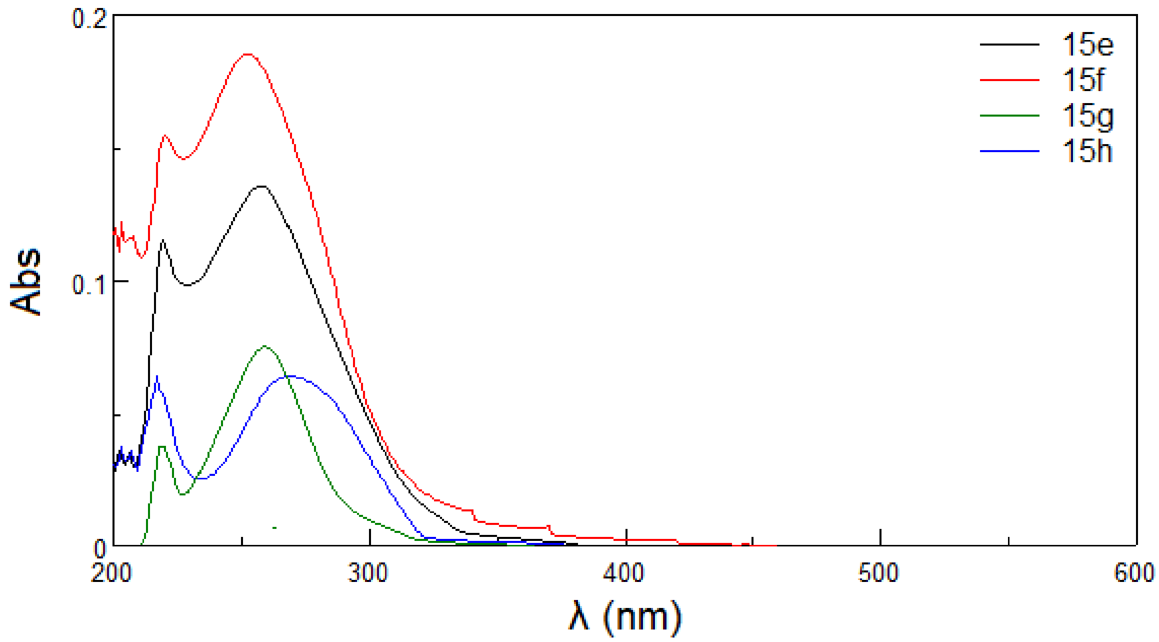

Figure S44. UV-Vis absorption spectra of **15e-f**

3. 3D fluorescence spectra

3D fluorescence spectra of compounds **10a-l** and **15a-h**. The color scale represents a flux of emitted photons. The number above color scales indicates the maximum relative value of emission intensity represented by color scale (and indicated in a respective figure). The unit of measurement in each spectrum represents the same number of emitted photons per second, i.e. fluorescence intensity values in all spectra can be directly compared.

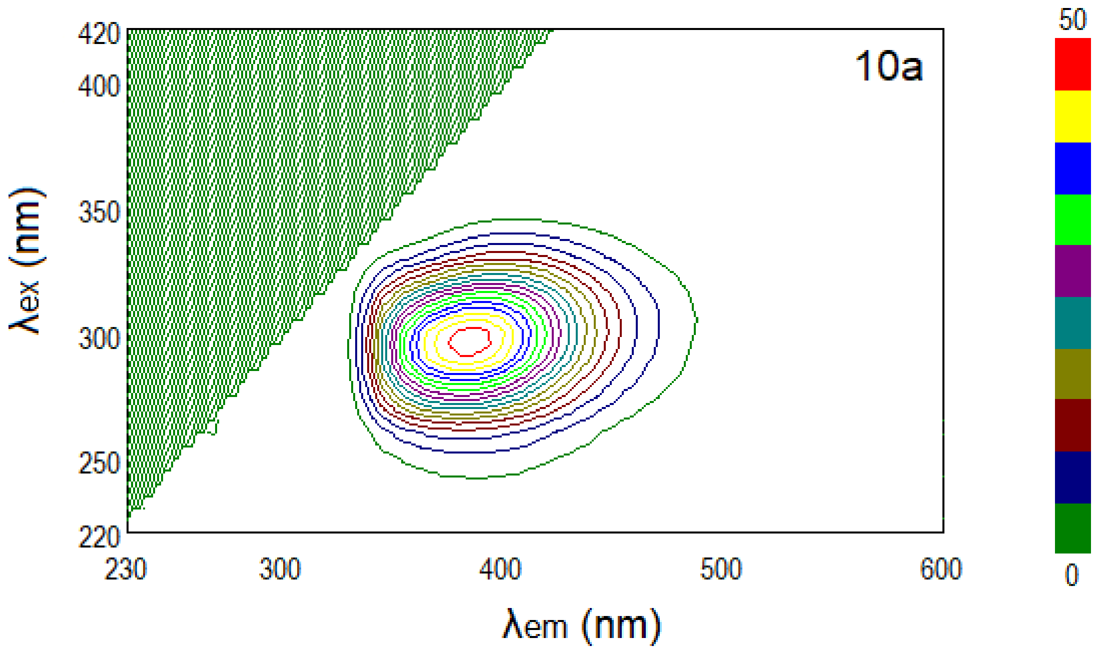

Figure S45. 3D fluorescence spectrum of **10a**

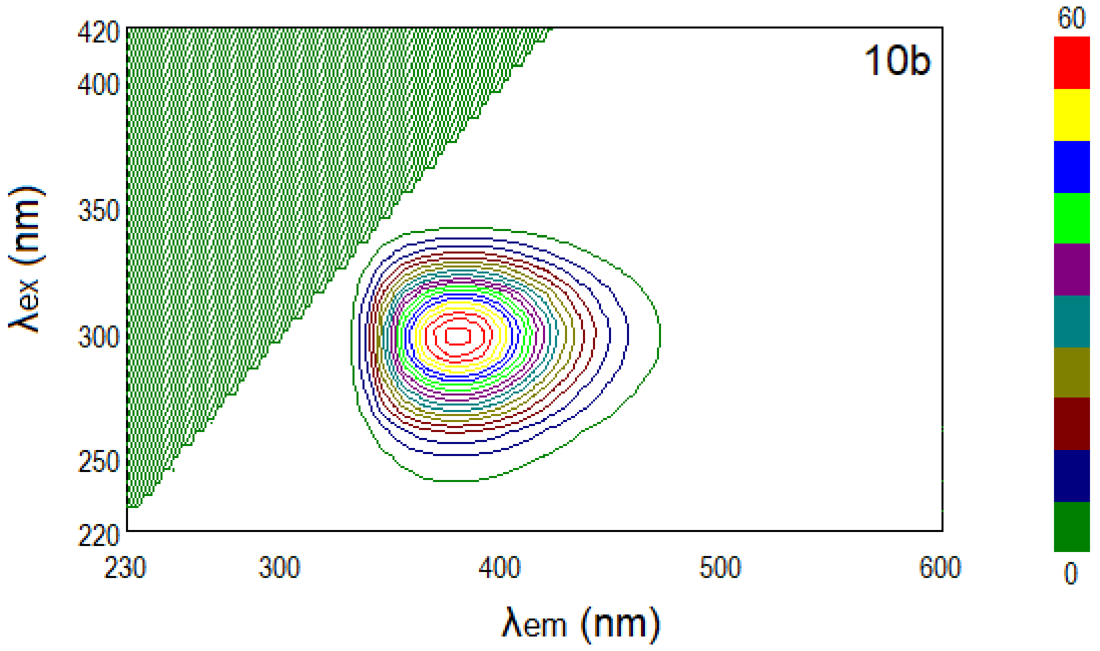

Figure S46. 3D fluorescence spectrum of **10b**

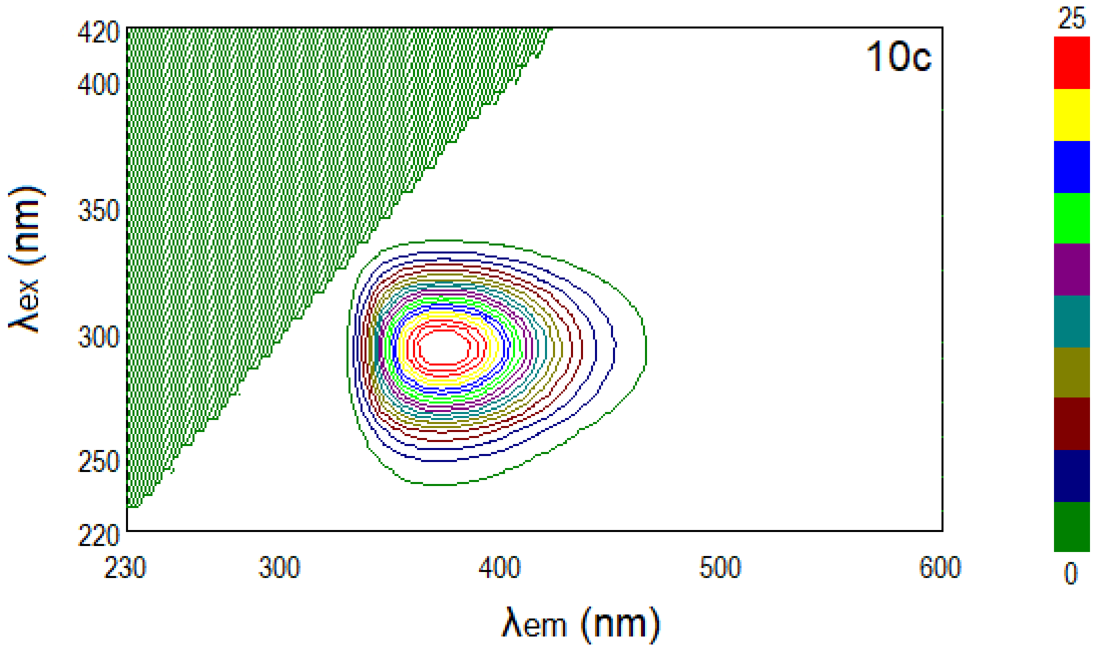

Figure S47. 3D fluorescence spectrum of **10c**

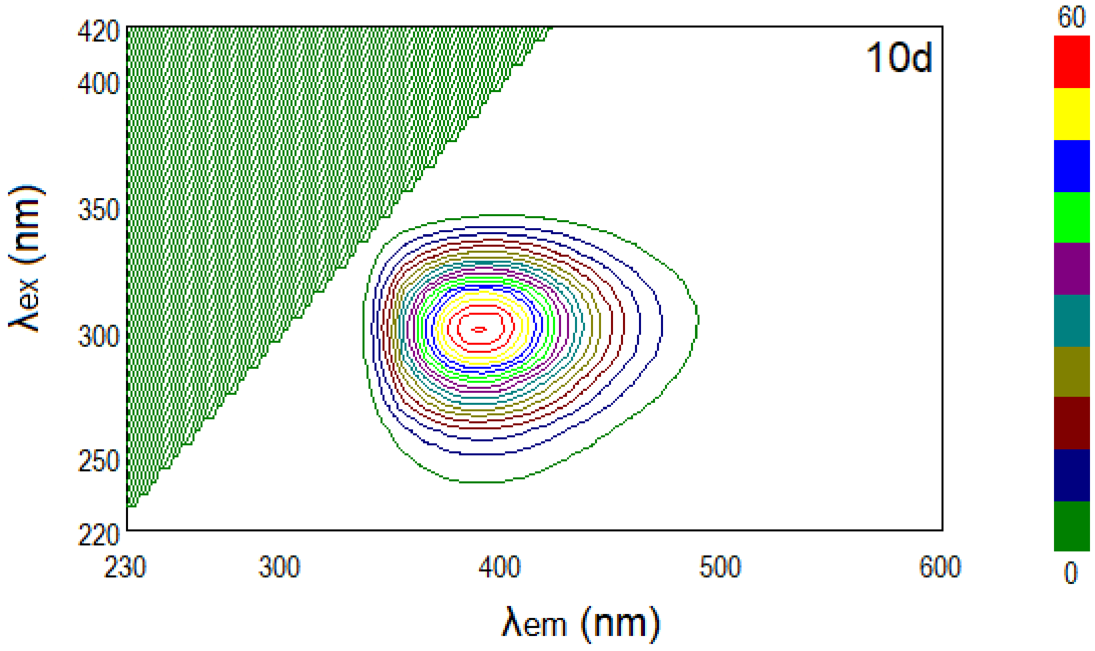

Figure S48. 3D fluorescence spectrum of **10d**

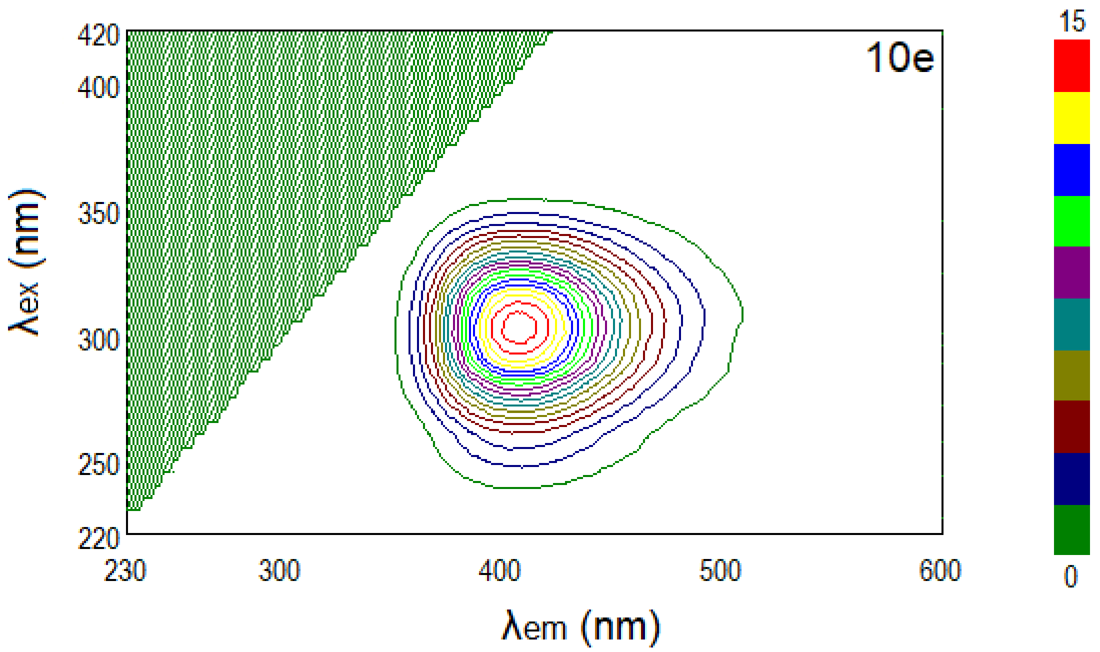

Figure S49. 3D fluorescence spectrum of **10e**

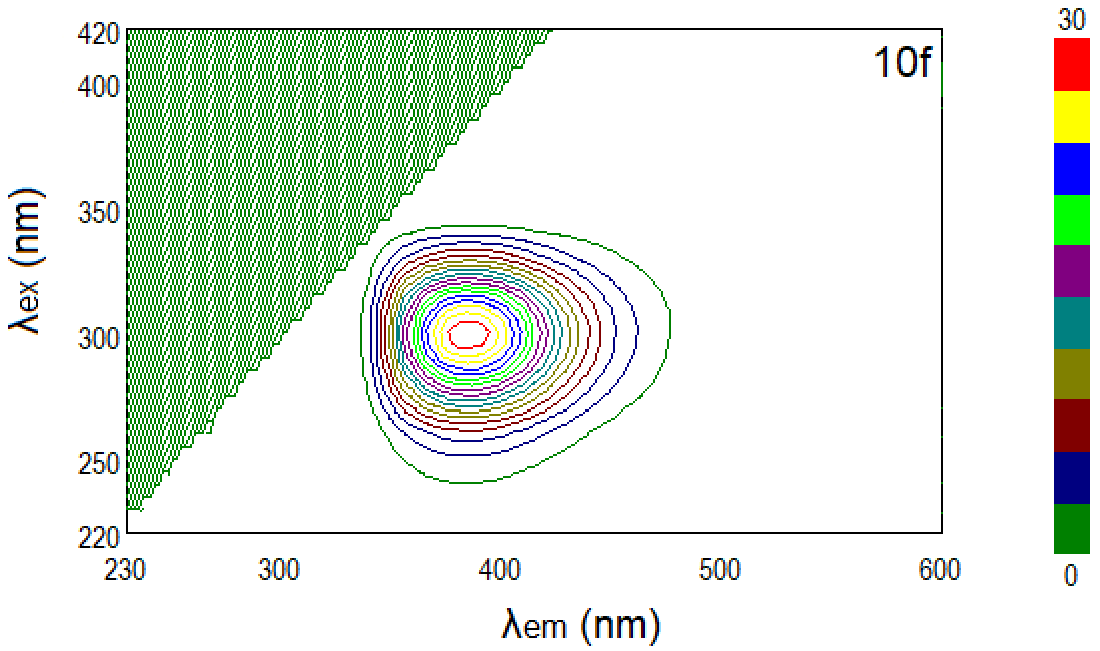

Figure S50. 3D fluorescence spectrum of **10f**

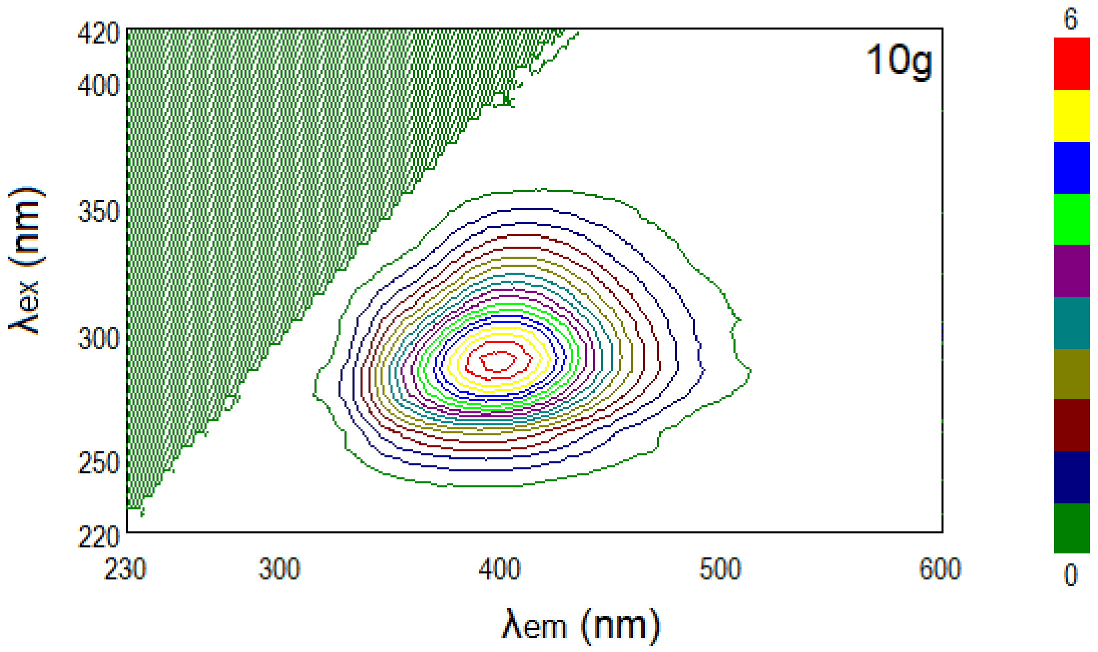

Figure S51. 3D fluorescence spectrum of **10g**

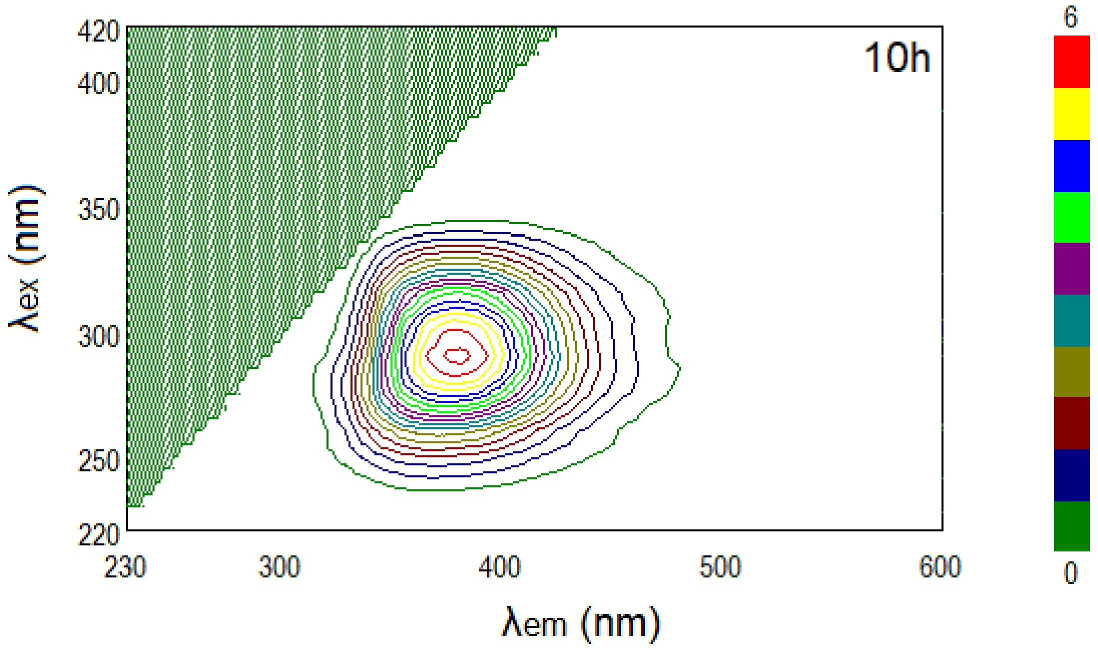

Figure S52. 3D fluorescence spectrum of **10h**

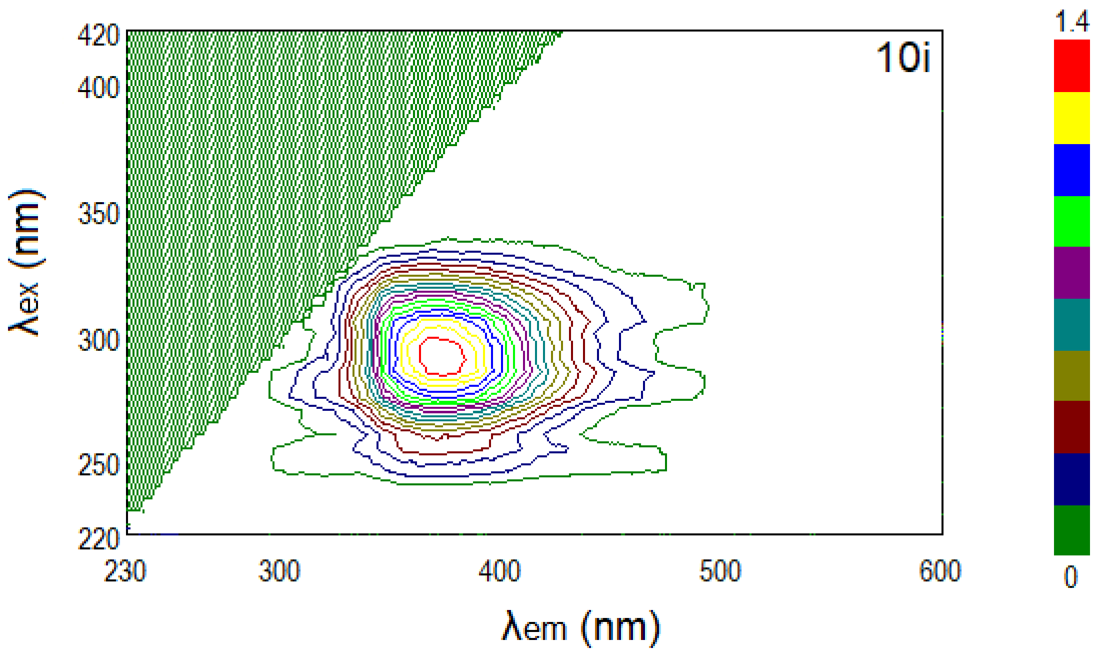

Figure S53. 3D fluorescence spectrum of **10i**

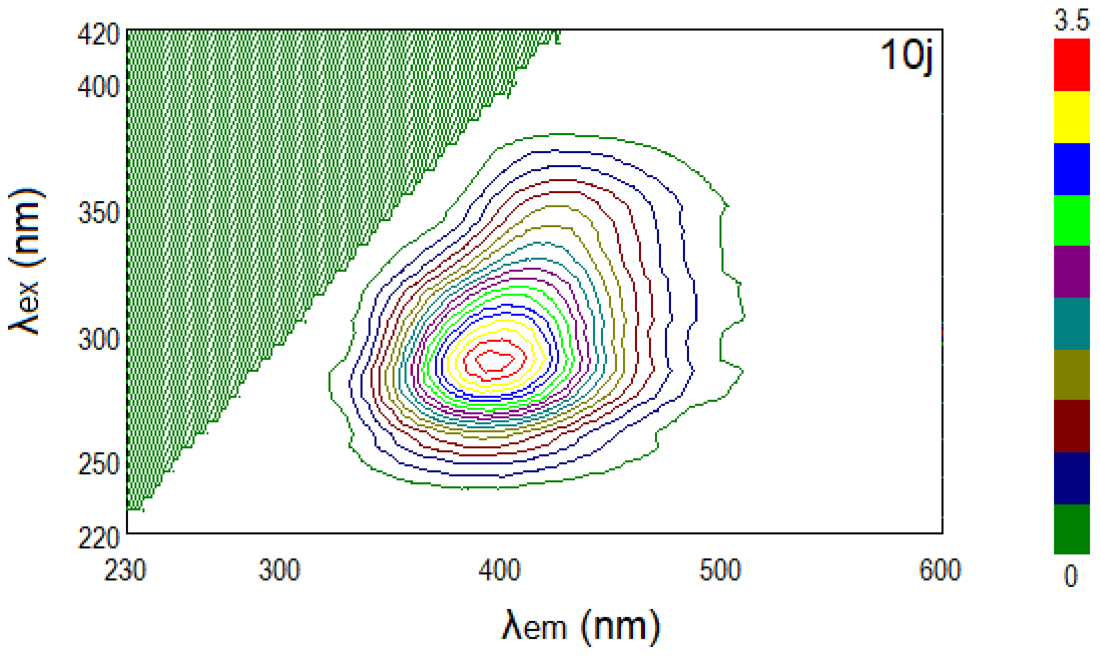

Figure S54. 3D fluorescence spectrum of **10j**

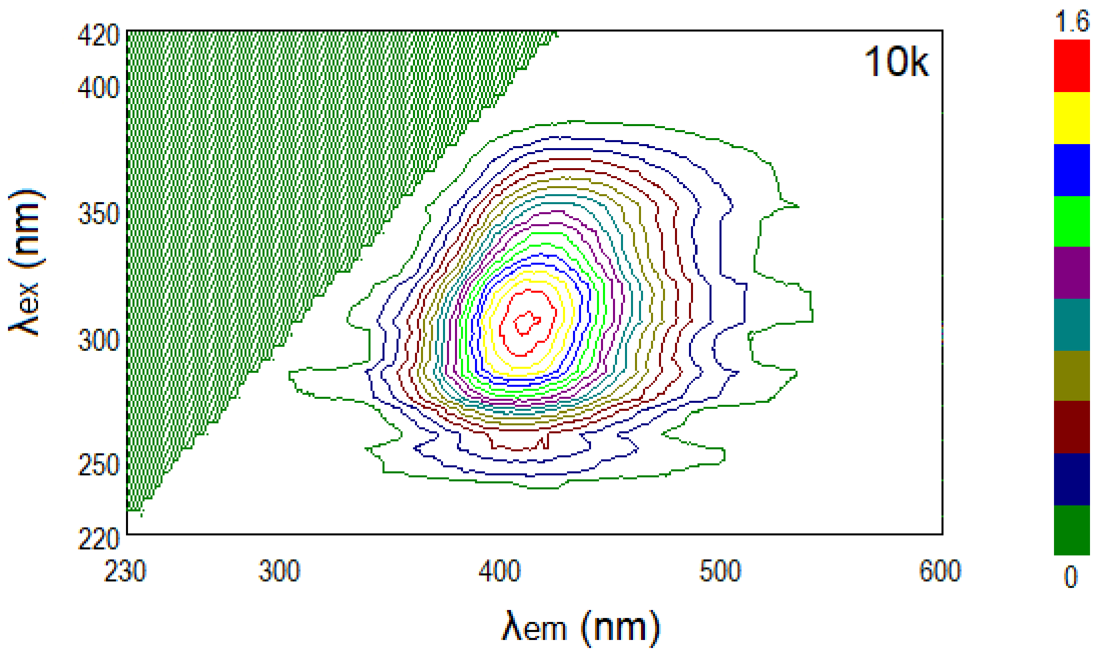

Figure S55. 3D fluorescence spectrum of **10k**

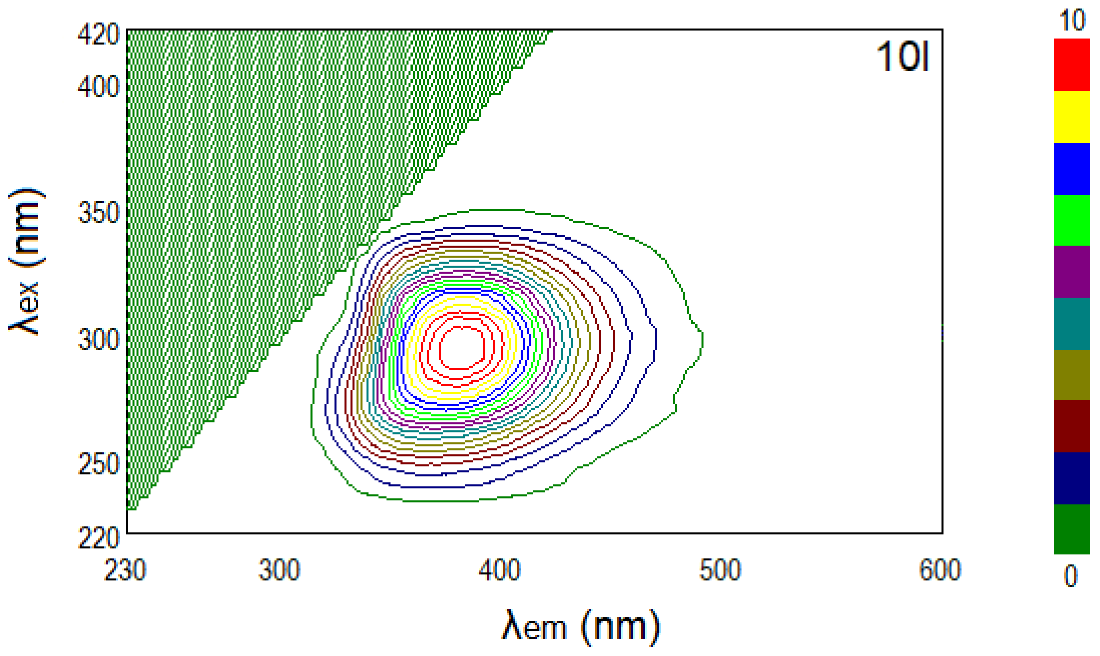

Figure S56. 3D fluorescence spectrum of **10l**

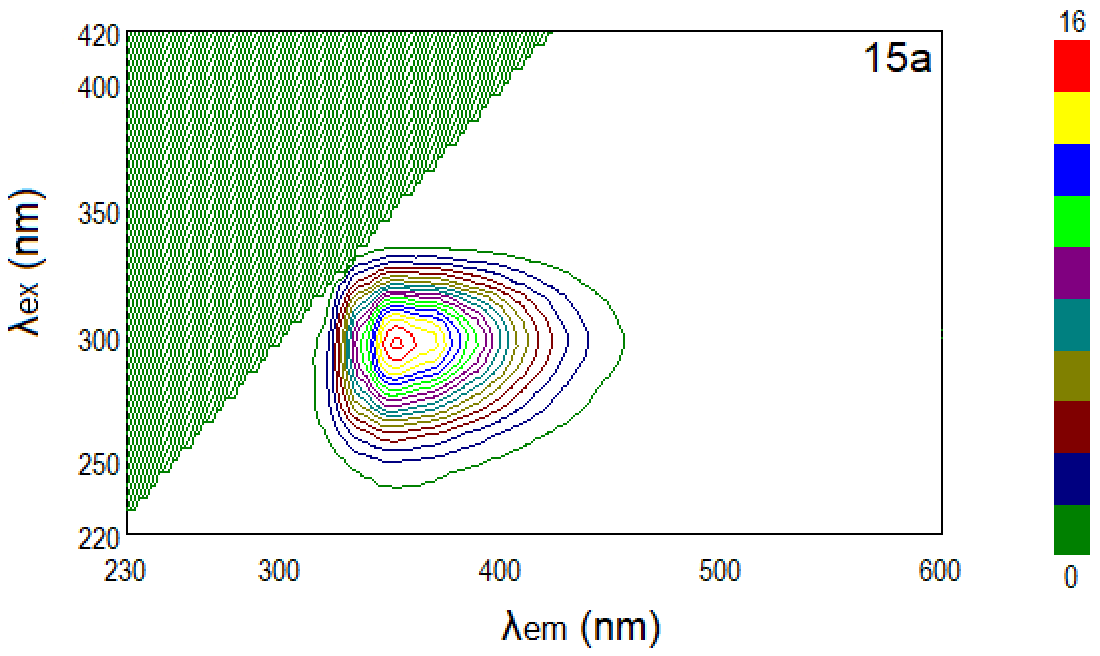

Figure S57. 3D fluorescence spectrum of **15a**

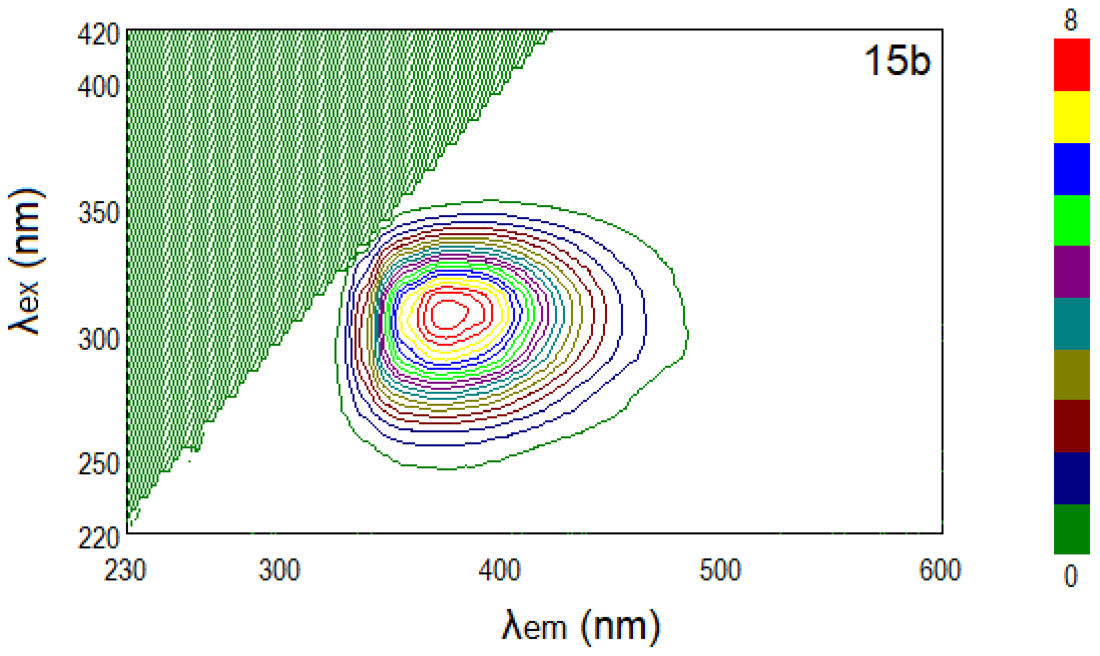

Figure S58. 3D fluorescence spectrum of **15b**

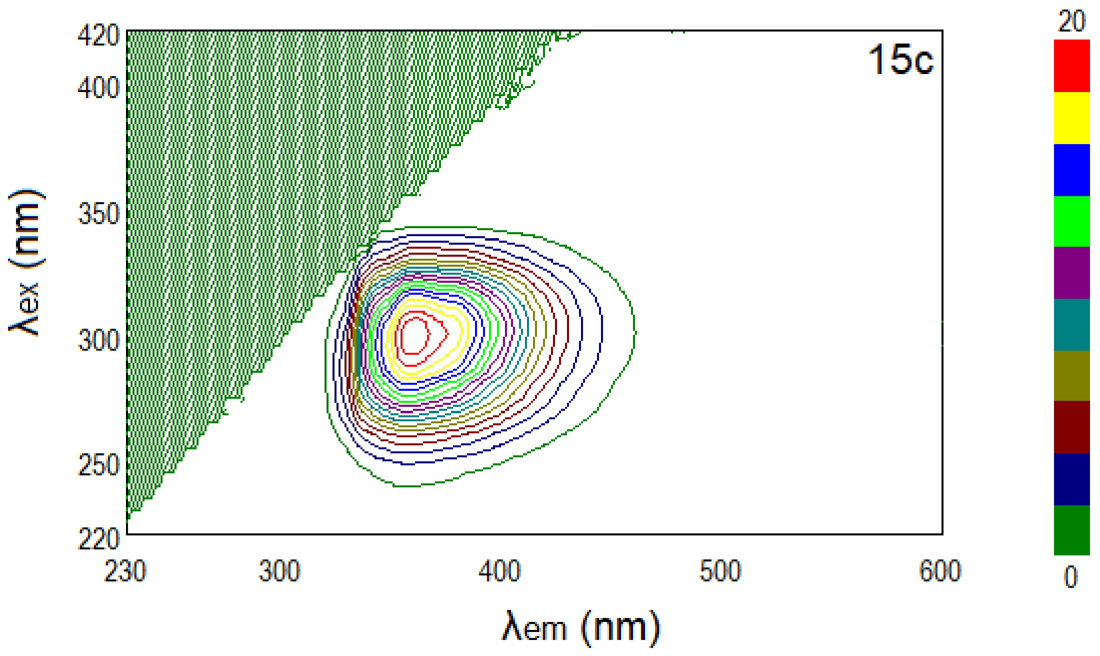

Figure S59. 3D fluorescence spectrum of **15c**

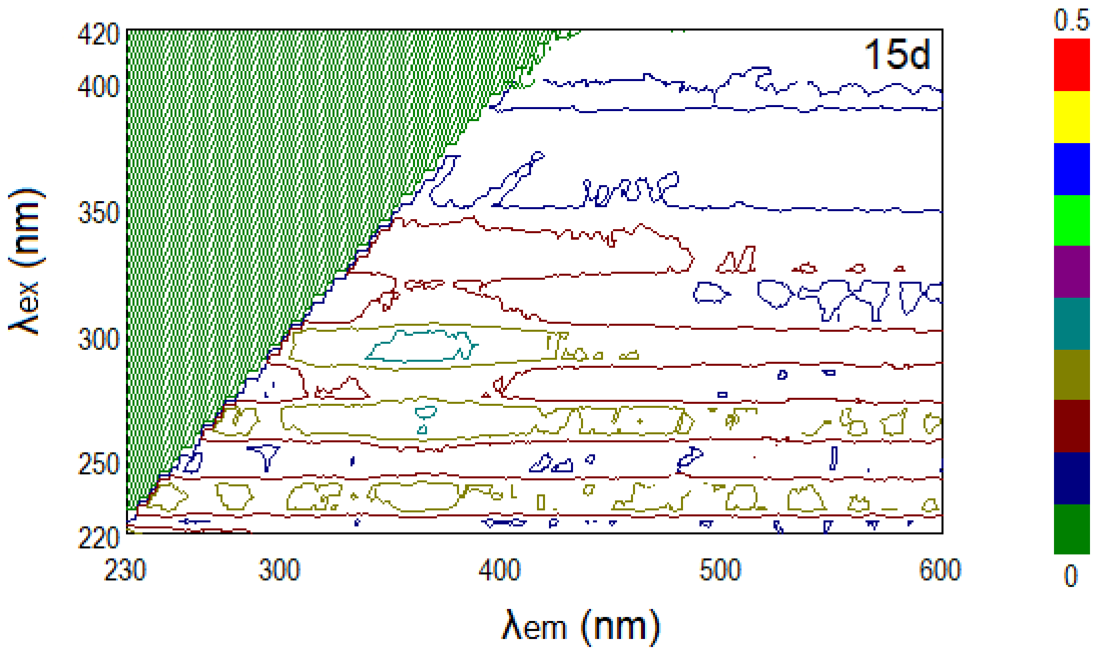

Figure S60. 3D fluorescence spectrum of **15d**

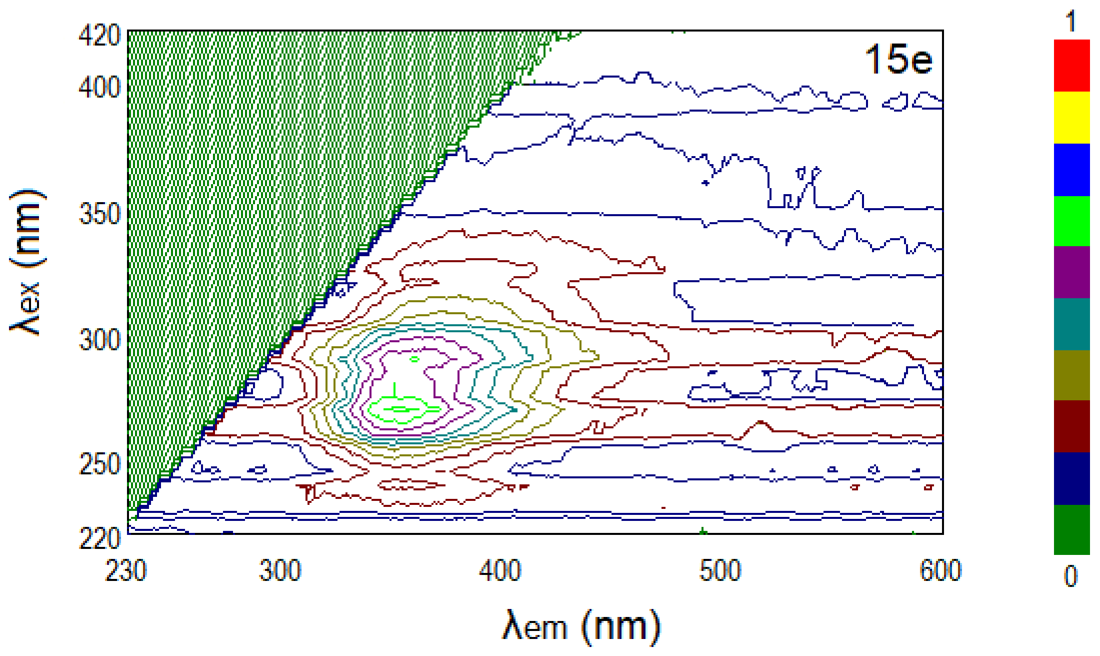

Figure S61. 3D fluorescence spectrum of **15e**

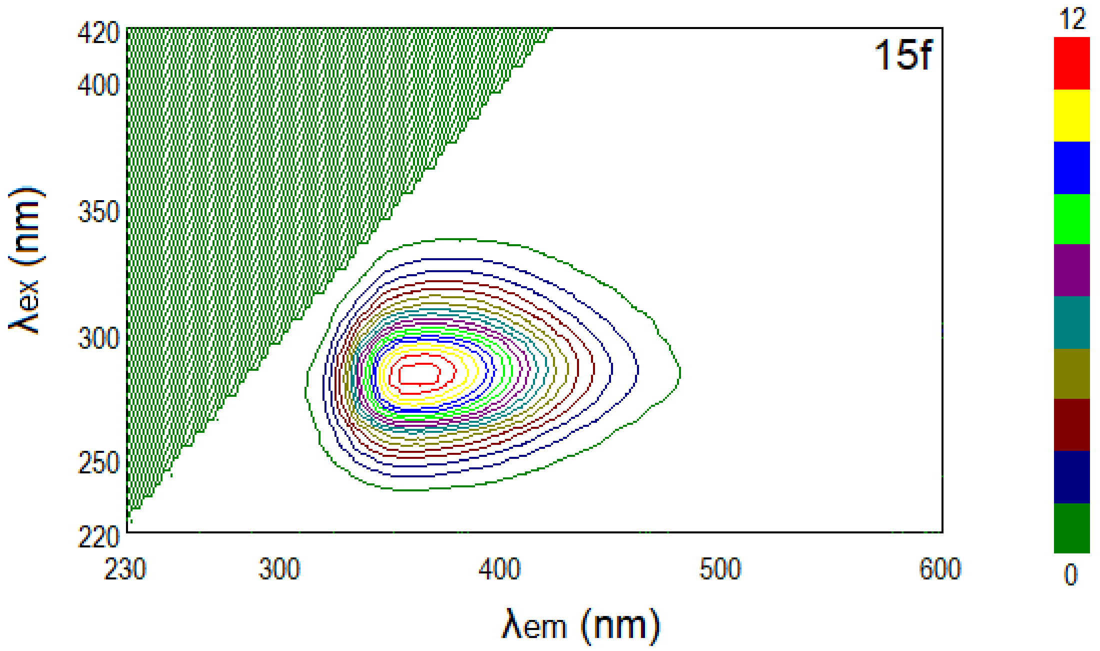

Figure S62. 3D fluorescence spectrum of **15f**

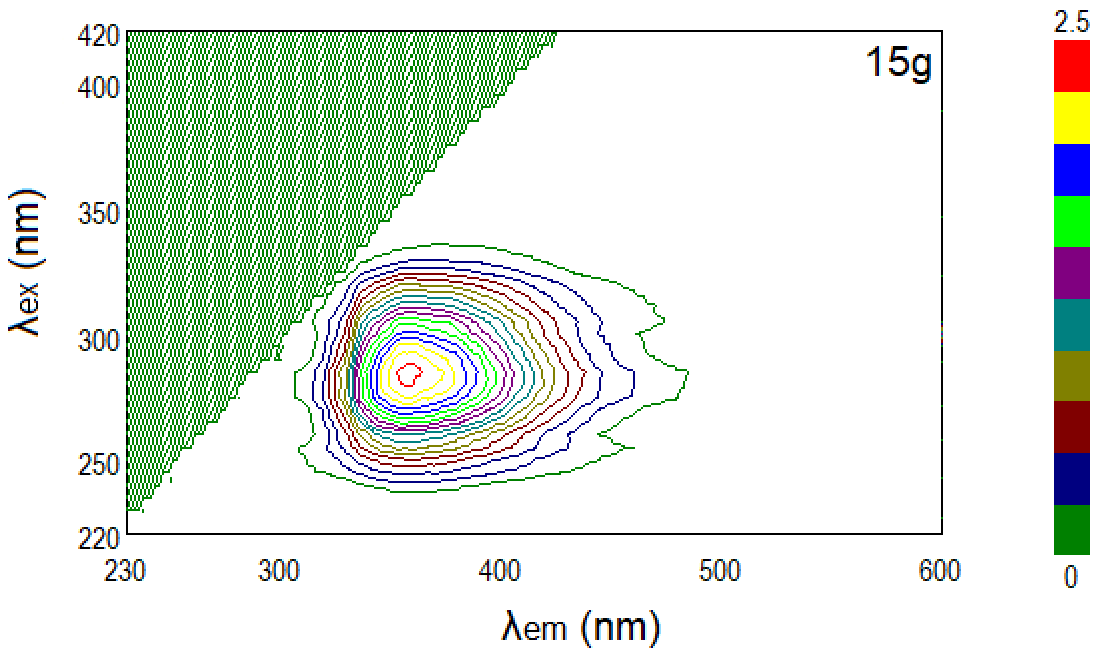

Figure S63. 3D fluorescence spectrum of **15g**

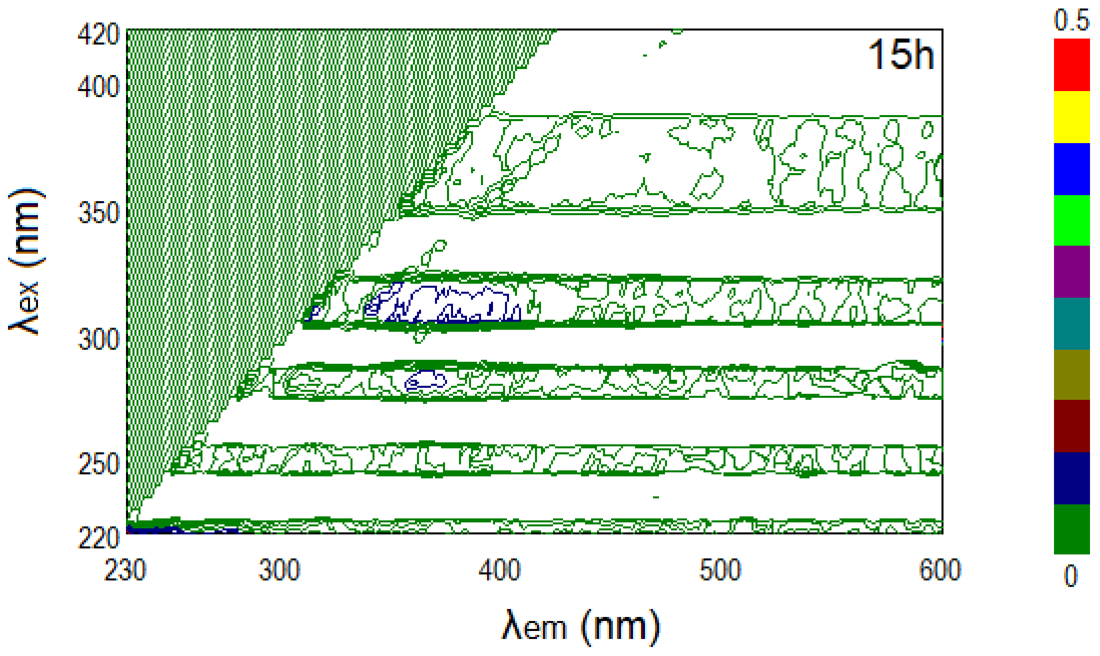

Figure S64. 3D fluorescence spectrum of **15h**

4. Emission data scatter plots

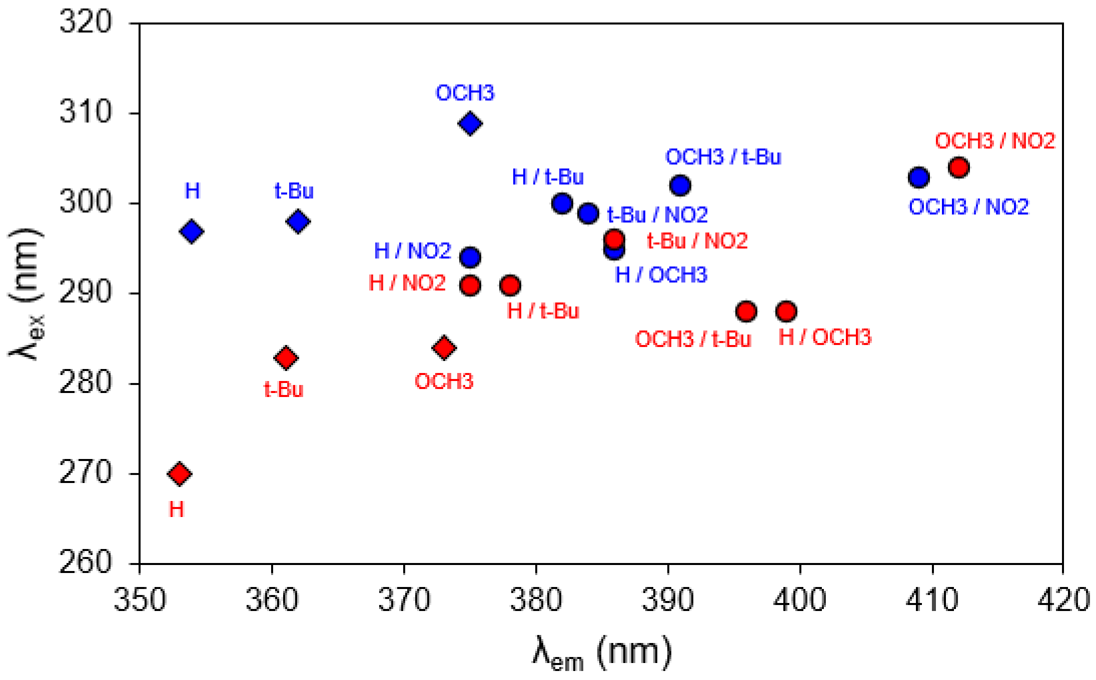

Figure S65. Position of global fluorescence maxima in the studied compounds. Circles and rhombuses indicate compounds of 10 and 15 series, respectively. Blue and red colors correspond to compounds respectively with Ph and *n*-Bu substituents in R^2 positions. Labels indicate R^1/R^3 substituents for 10 series and R^1 substituents for 15 series.

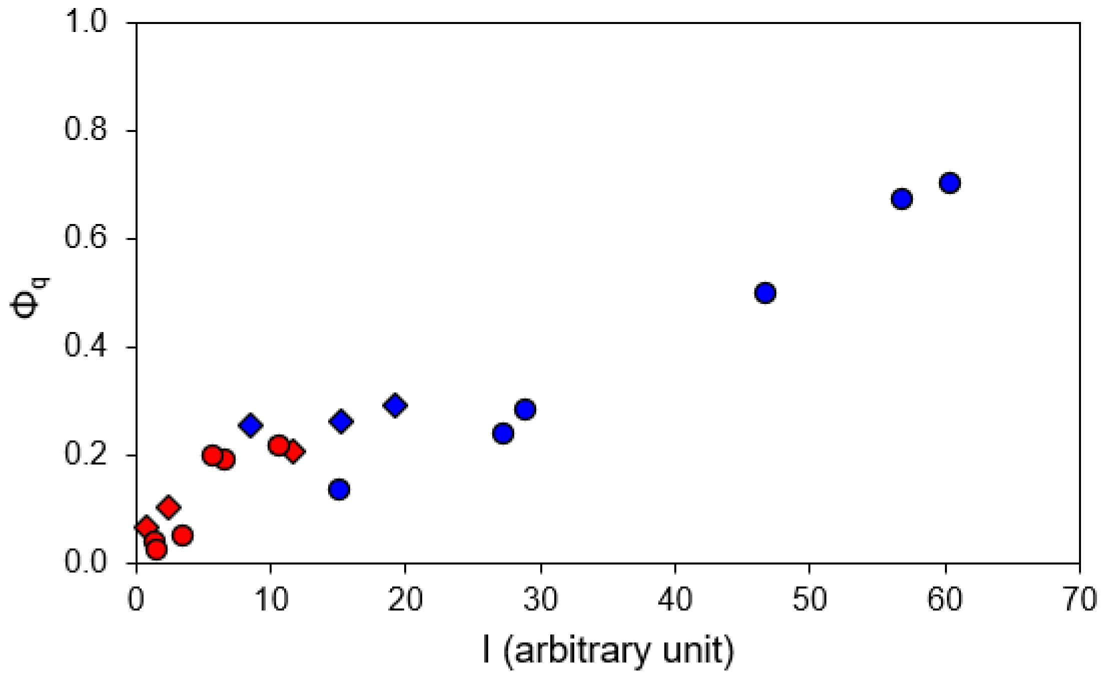

Figure S66. Quantum yields of the studied compounds as a function of the fluorescence intensity at a global maximum.Circles and rhombuses indicate respectively 10 and 15 series. Blue and red colors correspond to compounds respectively with Ph and *n*-Bu substituents in R^2 positions.

5. High-resolution mass spectra (HRMS (ESI)) results

Table S1. HRMS (ESI) results

Compound	M + H$^+$	m/z calcd	m/z found
**10a**	$C_{43}H_{30}N_{10}O$ +H$^+$	703.2682	703.2684
**10b**	$C_{46}H_{36}N_{10}$+H$^+$	729.3203	729.3202
**10c**	$C_{42}H_{27}N_{11}O_2$+H$^+$	718.2427	718.2425
**10d**	$C_{47}H_{38}N_{10}O$+H$^+$	759.3308	759.3309
**10e**	$C_{43}H_{29}N_{11}O_3$+H$^+$	748.2533	748.2531
**10f**	$C_{46}H_{35}N_{11}O_2$+H$^+$	774.3054	774.3056
**10g**	$C_{39}H_{38}N_{10}O$+H$^+$	663.3308	663.3309
**10h**	$C_{42}H_{44}N_{10}$+H$^+$	689.3829	689.3827
**10i**	$C_{38}H_{35}N_{11}O_2$+H$^+$	678.3054	678.3053
**10j**	$C_{43}H_{46}N_{10}O$+H$^+$	719.3934	719.3935
**10k**	$C_{39}H_{37}N_{11}O_3$+H$^+$	708.3159	707.3157
**10l**	$C_{42}H_{43}N_{11}O_2$+H$^+$	734.3679	734.3678
**15a**	$C_{30}H_{20}N_{10}$+H$^+$	521.1951	521.1952
**15b**	$C_{32}H_{24}N_{10}O_2$+H$^+$	581.2162	581.2160
**15c**	$C_{38}H_{36}N_{10}$+H$^+$	633.3203	633.3204
**15d**	$C_{30}H_{18}N_{12}O_4$+H$^+$	611.1652	611.1650
**15e**	$C_{26}H_{28}N_{10}$+H$^+$	481.2577	481.2578
**15f**	$C_{28}H_{32}N_{10}O_2$+H$^+$	541.2788	541.2789
**15g**	$C_{34}H_{44}N_{10}$+H$^+$	593.3829	593.3827
**15h**	$C_{26}H_{26}N_{12}O_4$+H$^+$	571.2278	571.2277

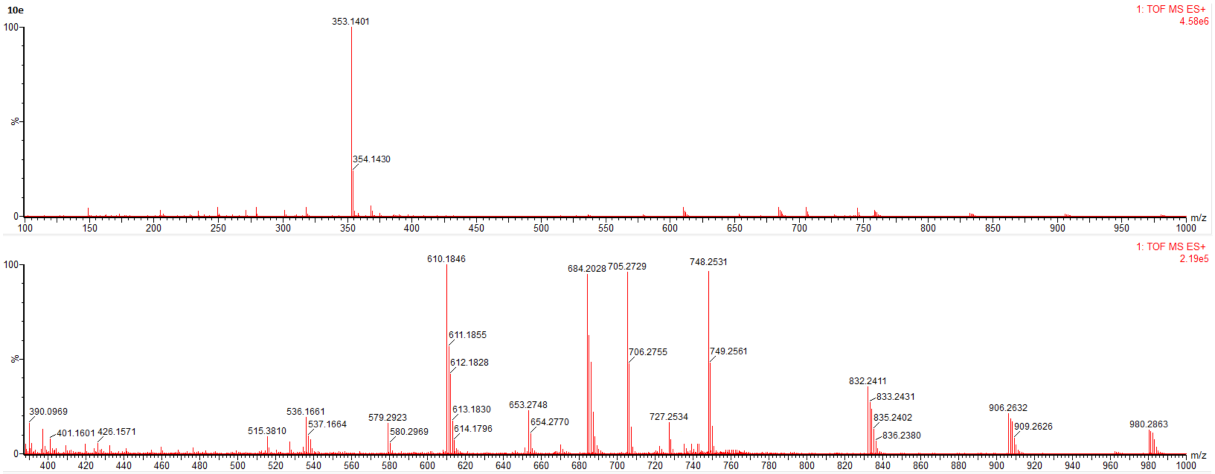

Figure S67. HRMS spectra of of 3-(4-(5-(4-methoxyphenyl)-4-phenyl-4*H*-1,2,4-triazol-3-yl)phenyl)-6-(4-(5-(4-nitrophenyl)-4-phenyl-4*H*-1,2,4-triazol-3-yl)phenyl)-1,2,4,5-tetrazine (**10e**)

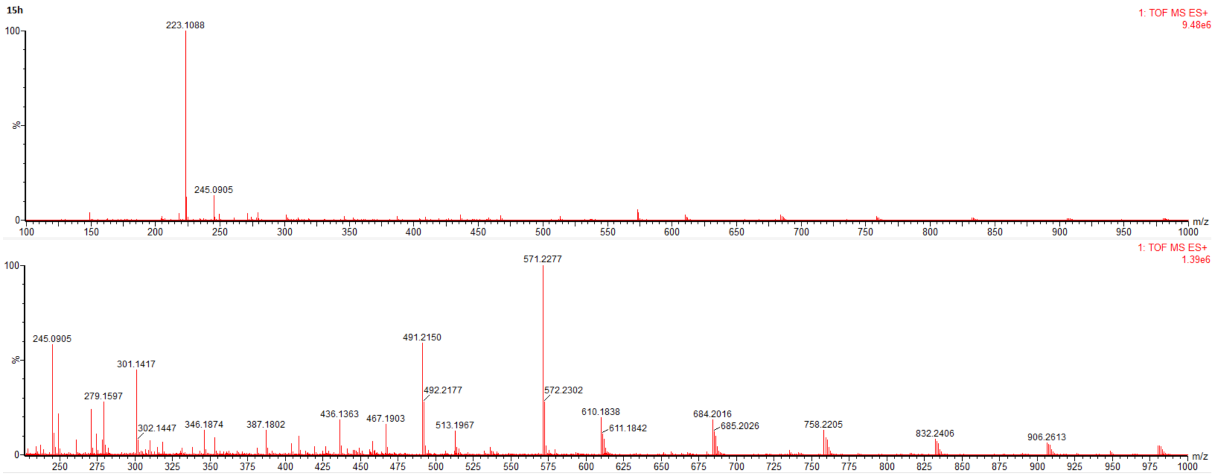

Figure S68. HRMS spectra of of 3,6-bis(4-butyl-5-(4-nitrophenyl)-4*H*-1,2,4-triazol-3-yl)-1,2,4,5-tetrazine (**15h**)
